# A calcium-sensing receptor allelic series and underdiagnosis of genetically driven hypocalcemia

**DOI:** 10.1016/j.ajhg.2025.06.013

**Published:** 2025-07-14

**Authors:** Jeremy B. Chang, Connor P. Barnhill, Alexander M. Apostolov, Marcus M. Soliai, Julian Hecker, Jovia L. Nierenberg, Lyndsay M. Stapleton Smith, Arun S. Mathew, Xue Zeng, Jiayin Diao, C. Dilanka Fernando, Qingwen Chen, Ben W. Dulken, Aleksandr Petukhov, Russ Altman, Tracy M. Josephs, Jessica A. Lasky-Su, Caroline M. Gorvin, Mary Scott Roberts, Scott H. Adler, Jonathan C. Fox, Christoph Lange, Sun-Gou Ji

**Affiliations:** 1 BridgeBio Pharma, 3160 Porter Drive, Suite 250, Palo Alto, CA 94304, USA; 2 Department of Bioengineering, Stanford University, Stanford, CA 94305, USA; 3 Channing Division of Network Medicine, Brigham and Women’s Hospital, Boston, MA 02115, USA; 4 Harvard Medical School, Boston, MA 02115, USA; 5 Drug Discovery Biology Theme, Monash Institute of Pharmaceutical Sciences, Monash University, Parkville, VIC 3052, Australia; 6 Department of Pathology, Stanford School of Medicine, Stanford, CA 94305, USA; 7 Departments of Bioengineering, Genetics, and Medicine, Stanford University, Stanford, CA 94305, USA; 8 Institute of Metabolism and Systems Research (IMSR) and Centre for Diabetes, Endocrinology and Metabolism (CEDAM), University of Birmingham, Birmingham B15 2TT, UK; 9 Centre for Membrane Proteins and Receptors (COMPARE), Universities of Birmingham and Nottingham, Birmingham B15 2TT, UK; 10 Channing Division of Network Medicine, Brigham and Women’s Hospital, Boston, MA 02115, USA; 11 Department of Biostatistics, Harvard T.H. Chan School of Public Health, Boston, MA 02115, USA

**Keywords:** autosomal-dominant hypocalcemia type 1, ADH1, rare disease, incomplete penetrance, variable expressivity, Mendelian disorder, monogenic disorder, hypoparathyroidism, hypocalcemia, symptom burden

## Abstract

The availability of genomic sequencing has revealed that variants in genes that cause rare monogenic disorders are relatively common, which raises the question of variant pathogenicity. Autosomal-dominant hypocalcemia type 1 (ADH1) is a rare genetic form of hypoparathyroidism caused by gain-of-function (GoF) variants in the calcium-sensing receptor (CaSR) encoded by *CASR*. We examined the prevalence, penetrance, and expressivity of GoF *CASR* variants in the UK Biobank (UKB; *n* = 433,793), All of Us (AOU; *n* = 229,987), and Mass General Brigham Biobank (*n* = 39,081). Individuals with previously reported ADH1-associated variants indeed showed ADH1 symptoms, including hypocalcemia (60% in the UKB and 78% in AOU). However, less than half had an ADH1-relevant diagnosis code (17% in the UKB and 44% in AOU), suggesting that individuals with ADH1 are present in these biobanks but may be underdiagnosed. We then developed a scoring algorithm and identified nine low-frequency ADH1-associated variants, which were further validated using genetic sequencing of individuals with nonsurgical hypoparathyroidism (*n* = 169) and an *in vitro* functional assay. These nine variants have an intermediate effect and frequency relative to previously reported ADH1-associated variants, completing an allelic series with respect to serum calcium, and alone are responsible for a symptom burden roughly equivalent to all previously reported ADH1-associated variants. Our work indicates that hypocalcemia due to GoF in *CASR* with ADH1-associated symptoms is underdiagnosed, provides a deeper understanding of the genotype-phenotype relationship of *CASR* variants, and illustrates that variants in genes underlying rare disorders may cause a much greater symptom burden than currently appreciated.

## Graphical abstract

**Figure:**
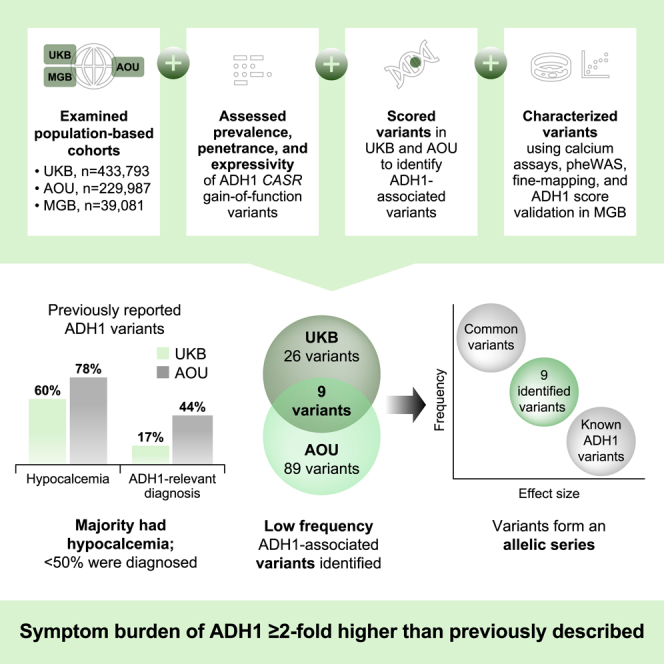

## Teaser

We examined variants of *CASR* associated with autosomal-dominant hypocalcemia type 1 (ADH1) and created a score to identify 9 additional variants. The symptom burden of ADH1 was ≥2-fold higher than previously appreciated. Our approach is applicable to other genetic diseases where symptom burden may also be higher than appreciated.

## Introduction

An estimated 1 in 10 people suffer from a rare disease, and 72% of these diseases are thought to have a genetic basis.^1^^,^^2^ Nevertheless, precise estimation of the symptom burden (i.e., number and severity of symptomatic individuals) of rare genetic disease is challenging given potential ascertainment bias, lack of specific diagnostic criteria, small numbers of affected individuals, and clinical heterogeneity. In addition, incomplete penetrance and variable expressivity of seemingly pathogenic variants complicate the matter further. The development of a framework for examining rare monogenic diseases within large population-based cohorts can enhance patient care by refining prevalence estimates, delineating symptom burden, elucidating variant penetrance and expressivity, and identifying additional variants that contribute to the phenotypic spectrum. A better understanding of disease-causing variants and their impact at a population level can bring resources and awareness to rare disease communities.

Autosomal-dominant hypocalcemia type 1 (ADH1; MIM: 601198) is a rare genetic form of hypoparathyroidism caused by gain-of-function (GoF) variants in the calcium-sensing receptor (CaSR) encoded by *CASR* (MIM: 601199). At the molecular level, the CaSR is a G-protein-coupled receptor (GPCR) that regulates calcium homeostasis, in part by modulating secretion of the parathyroid hormone.^3^ Historically, individuals with ADH1 have primarily been characterized in the context of small familial cohorts,^4^ which can lead to an incomplete understanding of the genotype-phenotype relationship.

So far, at least 121 *CASR* variants have been described in association with ADH1.^4^^,^^5^^,^^6^^,^^7^^,^^8^ Loss-of-function (LoF) variants are also observed in association with familial hypocalciuric hypercalcemia type 1 (FHH1; MIM: 145980).^9^ These LoF variants are more frequent in the general population than GoF variants and can complicate the identification of GoF variants, especially when functional characterization is not performed because both are considered pathogenic.

Phenotypically, ADH1 has variable expressivity where individuals with the same genetic variant can show a wide range of clinical symptoms.^10^ It is primarily characterized by persistent hypocalcemia in the setting of low parathyroid hormone levels. This hypocalcemia can cause paresthesia, tetany, and, in severe cases, cardiac arrhythmias, laryngospasm, and seizures. Individuals can experience hypercalciuria, which can be exacerbated by calcium supplementation, leading to kidney stones and chronic kidney disease. ADH1 has also been described with features of Bartter’s syndrome, including hypomagnesemia, hypokalemia, and metabolic alkalosis.^11^ Therefore, diagnosing ADH1 is not straightforward, and no specific diagnosis code existed until October 2023.

To clarify the genotype-phenotype relationship of *CASR* variants, we characterized variants and associated phenotypes in three population-based biobanks: the UK Biobank (UKB), All of Us (AOU), and the Mass General Brigham (MGB) Biobank. Using the example of ADH1, we provide a framework to identify and characterize pathogenic variants of rare diseases in large biobanks and illustrate how such an approach can complement studies from familial and clinically ascertained cohorts.

## Subjects, material, and methods

### UKB

The UKB is a large-scale biomedical database containing genetic, lifestyle, health, and clinical information from 500,000 participants.^12^^,^^13^ Self-reported ethnicities from the UKB that were included in our analysis are “Black African,” “Caribbean,” “Chinese,” “White British,” “Indian,” and “Pakistani.” Quality control on all qualifying variants within the Matched Annotation from NCBI and ENBL-EBI (MANE) transcript of *CASR* (ENST00000639785.2, GenBank: NM_000388.4)^14^ was performed using PLINK (v.2.00a3.1LM)^15^ and HAIL (v.0.2.78).^16^ Serum calcium levels were uncorrected for albumin. The estimated glomerular filtration rate (eGFR) was calculated using the 2012 CKD-EPI cystatin C equation.^17^ We extracted ICD-10 codes and medications from both in-patient and general practitioner (GP) data. ICD-10 codes were then mapped to a total of 1,518 phenotypes (phecodes) using the Phecode Map 1.2.^18^ Phecodes we considered to be ADH1 associated were 252.2 (hypoparathyroidism), 275.5 (disorders of calcium or phosphorous metabolism), 350.1 (tetany), 687.4 (paresthesia), and 345.0/345.1/345.11/345.12 (various forms of epilepsy). Locus-phenotype associations were tested using Fisher’s exact test, and for locus-phenotype combinations with at least 10 events per variable, we used covariate/PC-adjusted logistic regression with Firth correction. Based on the similarity of the results from logistic regression with Firth correction and Fisher’s exact test for combinations with more than 10 events per variable (Table S1) and the substantial number of combinations with fewer events, we used *p* values from Fisher’s exact test for subsequent analyses. For medications, the count of instances of “calcichew,” “calcichew d3 tablet,” “colecalciferol,” “cholecalciferol,” “calcitriol,” “calcium citrate,” “calcium carbonate,” “alfacalcidol,” “PTH,” “natpar,” “teriparatide,” and “forteo” was determined for each individual, and they were considered ADH1-associated medications. All regression analyses were conducted separately in each self-reported ethnicity group, and the results were meta-analyzed. Further details on the study, exome sequencing, and phenotypes are in the supplemental methods.

### AOU

The AOU Research Program is a longitudinal cohort study led by the National Institutes of Health for the advancement of precision medicine.^19^ Genetically determined ancestry groups in AOU are genetic ancestries similar to the African ancestry (AFR), American ancestry (AMR), East Asian ancestry (EAS), European ancestry (EUR), Middle Eastern ancestry (MID), and South Asian ancestry (SAS) 1000 Genomes population references and will be referred to as AFR-like, AMR-like, EAS-like, EUR-like, MID-like, and SAS-like, respectively. For serum calcium levels, we used the concept “calcium [mass/volume] in serum or plasma,” and for serum phosphate levels, we used “phosphate [mass/volume] in serum or plasma.” For diagnosis codes, we considered the same phecodes that we used in the UKB. To capture phenotype data originally recorded using SNOMED terms in AOU, these phecodes were mapped to ICD-10 codes, which were mapped to SNOMED using the Owlready2 library’s Pymedtermino2 module,^20^ which utilizes Unified Medical Language System (UMLS) data. For ADH1-associated medications, we used concept IDs corresponding to calcitriol, colecalciferol, and teriparatide. Additional information on the study, whole-genome sequencing, and phenotypes are in the supplemental methods.

### Variant scoring in UKB and AOU

To prioritize pathogenic ADH1 variants,^21^^,^^22^^,^^23^^,^^24^^,^^25^^,^^26^^,^^27^^,^^28^^,^^29^^,^^30^ we calculated a variant score as the sum of sub-scores indicating the strength of association with ADH1 phenotypes and other characteristics consistent with a GoF variant. We refer to this score as a variant’s ADH1 score, which was composed of the variant’s association with serum calcium and phosphate levels, the presence of the variant in any individual with an ADH1-associated diagnosis or medication, whether the location of the variant was in a hotspot for a known GoF variant (amino acids 116–136^5^ and 819–837^31^), and an ensemble of *in silico* pathogenicity predictors through Varsome.^32^ The significance of the sub-scores was tested using Fisher’s exact test for binary phenotypes/logistic regression with Firth correction or linear regression for quantitative phenotypes. The weights of the sub-scores were defined as 3 for serum calcium, 1 for serum phosphate (since fewer, ∼50%, individuals are hyperphosphatemic), 1 for each of the ADH1-associated diagnoses or medications, 0.5 for whether it occurred at the same location as a known GoF variant, and 0.2 for Varsome predictions. All variants remaining after hard filtering were scored. Details are described further in the supplemental methods, and the correlation matrix between the sub-scores for each self-reported ethnicity is shown in Table S2. The threshold for a significance of 1.5 was defined such that 98% of the scores from the null distribution (created by resampling sub-scores of synonymous variants) fell below that threshold (Figure S1). This threshold yielded the expected specificity of 0.98 with respect to synonymous variants and a sensitivity of 0.75 in a subset of participants with self-reported White British ethnicity determined to be genetically homogeneous by principal-component analysis (Field 22006) and had similar sensitivity and specificity in all other self-reported ethnicities (Field 21000) in the UKB based on known ADH1 pathogenic variants (Table S3). Self-reported ethnicity is used as a proxy for genetic ancestry because a true genetically homogeneous population is not provided in the UKB for ancestries other than White British. This score and threshold were then applied to all genetic ancestries in AOU, which showed consistent sensitivity and specificity (Table S4). Full summary statistics of variants considered in this study are available in the supplemental information. The code for calculating the ADH1 variant score is available on Zenodo (https://doi.org/10.5281/zenodo.15428300).

### *In vitro* and computational analyses

To generate variant CASR cell lines, wild-type (WT) and cmyc-tagged variant *CASR*s were integrated into FlpIn TREx HEK293 cells (Invitrogen) using isogenic integration at the Flp-recombinase integration site, and cells underwent hygromycin antibiotic selection to ensure a single copy was integrated per cell. cmyc-CaSR_variant_ expression was under the control of tetracycline, thus allowing titration of cmyc-CaSR_variant_. The response of each CaSR variant was then determined using a Ca_i_^2+^ mobilization assay using fluorescence-activated cell sorting (FACS) analysis on a FACS Canto II (Becton Dickinson) as described previously.^33^

The excess burden of disease was calculated using phecode-based phenotypes. A pheWAS of *CASR* variants was conducted using the SKAT-O test via the SKAT() function from the SKAT R package (v.2.2.5).^34^ We also performed a logistic regression using the Python statsmodels package v.0.14.1 to determine the direction of effect for these variants. Age, sex, and the first 10 genetic principal components were used as covariates. We fine mapped the *CASR* region and tested for colocalization with 55 other phenotypes using Coloc-SuSiE.^35^ Experimental details and computational methods are described further in the supplemental methods.

### MGB Biobank

The analysis in the MGB^36^ utilized exome sequencing data from approximately 54,000 participants. Following the removal of related individuals, we retained 39,081 individuals. Calcium levels were averaged across repeated measurements for each participant. Close relative pairs were identified based on inferred kinship coefficients and excluded, with a KING kinship coefficient cutoff of 0.0884. Merging genetic and phenotype data resulted in *n* = 35,509 participants for the calcium analysis. The analysis of calcium levels was performed using a linear regression model with sex and the first 10 genetic principal components as covariates.

### Ethics

Each study was approved by study-specific institutional review boards, and informed consent was obtained from all study participants. The publication of summaries involving fewer than 20 participants was approved by special exemption from the AOU Research Program Resource Access Board due to the rarity of ADH1. To protect participant privacy, demographic details that could potentially lead to re-identification have been excluded.

## Results

### Confirmation that *CASR* variants previously associated with ADH1 were associated with ADH1 phenotypes in the UKB and AOU

Five previously reported ADH1-associated variants^4^^,^^5^^,^^6^^,^^7^^,^^8^ (c.310G>A [p.Val104Ile], c.372C>A [p.Asn124Lys], c.452C>T p.Thr151Met], c.613C>T [p.Arg205Cys], and c.2663C>T [p.Thr888Met]) were found in a heterozygous state in 10 individuals in the UKB (7 females and 3 males; Table 1; a systematic review of the location and structural modeling of the variants is provided in Table S5, and a structural analysis of the variants is shown in Figure S2). The majority of individuals in the UKB had only a single measurement of serum calcium (85%; Figure S3A), and no significant differences in mean serum calcium level were detected across self-reported ethnicities (ANOVA; Figure S3B). Of the 10 heterozygotes with reported values (Figure S3C), six (or 60%) had serum calcium below the lower limit of normal (LLN; 2.2 mM^37^), whereas only 2% of UKB participants, overall, had serum calcium below the LLN (*p* = 1.0E−11, t test; Table 1; Figure S3D). Four of 10 heterozygotes were above the upper limit of normal (ULN) for serum phosphate (1.45 mM^38^; Table 1), which is similar to previous reports.^4^ No significant differences in mean serum phosphate levels were detected across self-reported ethnicities except between the UKB self-reported Black African and Indian, White British and Indian, and Caribbean and Indian cohorts (*p* = 1E10−4, 2.6E−2, and 8.7E−3, respectively; Tukey honestly significant difference [HSD]; Figure S3E). At least one ADH1-related medication or symptom was observed (nominal significance threshold *p* < 0.05) in individuals with c.310G>A (p.Val104Ile), c.372C>A (p.Asn124Lys), and c.452C>T (p.Thr151Met). Three individuals outside of the analysis cohort due to self-reported ethnicity or relatedness exclusions had a previously reported ADH1 variant: c.380A>G (p.Glu127Gly), c.310G>A (p.Val104Ile), or c.613C>T (p.Arg205Cys) (Table S6).

**Table 1:** Characteristics of variants previously associated with ADH1 detected in the UKB

**Variant**	**Self-reported ethnicity**	**No. het**	**No. hom**	**Frequency in biobank**	**MAF in ethnicity**	**Calcium**			**Phosphate**			**eGFR**_**cy**_		**Age**	**Sex**	**Ca**^**2+**^**(mM)**	**Phosphate (mM)**	**Relevant meds**	**eGFR**_**cy**_**(mL/min/1.73 m**^**2**^**)**	**Diagnoses**
						**Mean (mM)**	**Beta (mM)**	***p* value**	**Mean (mM)**	**Beta (mM)**	***p* value**	**Beta (mL/min/1.73 m**^**2**^**)**	***p* value**							
c.310G>A (p.Val104Ile)^a^	White British	1	0	7.4E−06	2.8E−06	2.13	−0.24	9.1E−03	1.46	0.35	2.2E−02	−4.1	6.9E−01	59	M	2.13^b^	1.46^c^	N/A	76.5	N/A
	Indian	3	0	7.4E−06	7.8E−04	2.14	−0.24	1.8E−06	1.56	0.41	1.2E−06	−0.6	9.5E−01	55	M	2.07^b^	1.44	N/A	79.5	N/A
			0											55	F	2.07^b^	1.70^c^	N/A	78.5	N/A
			0											58	M	2.19^b^	1.51^c^	N/A	81.4	tetany (OR 157.6, *p* = 0.01)^d^
c.372C>A (p.Asn124Lys)^a^	White British	2	0	2.1E−06	5.5E−06	2.20	−0.19	3.1E−03	1.25	0.05	6.4E−01	−20.4	4.4E−02	70	F	2.19^b^	1.27	calcitriol	71.8	hypoparathyroidism (OR 836, *p* = 2.4E−03)^d^
			0											53	F	2.20^b^	1.23	N/A	66.0	N/A
c.452C>T (p.Thr151Met)^a^	White British	1	0	1.1E−06	2.8E−06	1.96	−0.43	4.5E−06	1.57	0.38	1.5E−02	10.0	4.9E−01	52	F	1.96^b^	1.57^c^	N/A	109.4	N/A
c.613C>T (p.Arg205Cys)	Chinese	1	0	2.1E−06	7.2E−04	2.39	−0.01	9.0E−01	1.04	−0.21	1.8E−01	10.8	3.4E−01	66	F	2.39	1.04	N/A	103.6	N/A
c.2663C>T (p.Thr888Met)	White British	2	0	2.1E−06	5.5E−06	2.29	−0.10	1.3E−01	1.33	0.14	2.1E−01	5.4	6.0E−01	60	F	2.22	1.37	N/A	98.8	N/A
			0											59	F	2.36	1.29	N/A	95.1	N/A

eGFR_cy_, estimated glomerular filtration rate calculated using the CKD-EPI cystatin C equation (2012).^17^ Bonferroni significance thresholds were 7.1E−3 for calcium, phosphate, and eGFR_cy_ and 6.2E−3 for medications and diagnoses. Lower limit of normal (LLN) calcium is 2.2 mM, and upper limit of normal (ULN) phosphate is 1.45 mM. OR and *p* values in the “relevant meds” and “diagnoses” columns are for the medication or diagnosis for the variant in each self-reported ethnicity. F, female; het, heterozygous; hom, homozygous; Inf, infinite; M, male; MAF, minor-allele frequency; meds, medications; OR, odds ratio.

a At least one nominally significant association.

b Below LLN.

c Above ULN.

d *p* < 0.05 (nominal significance threshold).

In AOU, eight previously established ADH1 variants were detected across 16 individuals^21^^,^^23^^,^^39^^,^^40^^,^^41^^,^^42^ (Table 2; a systematic review of the location and structural modeling of the variants is provided in Table S5, and structural analysis of the variants is shown in Figure S2). We detected at least one nominal ADH1-related association (nominal significance threshold *p* ≤ 0.05) in six of eight variants. All six had at least a nominally significant association with reduced serum calcium levels in at least one genetically determined ancestry, including two of the three variants also found in the UKB (c.310G>A [p.Val104Ile] and c.452C>T [p.Thr151Met]). Three of these variants were also associated with calcium-increasing medications (c.310G>A [p.Val104Ile], c.2431A>G [p.Met811Val], and c.2503G>A [p.Ala835Thr]). At least nominally significant associations between three out of eight variants with ADH1-related diagnoses were detected (Table 2).

**Table 2:** Characteristics of variants previously associated with ADH1 detected in AOU

**Variant**	**Ancestry**	**No. het**	**No. hom**	**Frequency in biobank**	**MAF in ancestry**	**Calcium**			**Phosphate**			**Medications**				**Diagnoses**
						**Mean (mM)**	**Beta (mM)**	***p* value**	**Mean (mM)**	**Beta (mM)**	***p* value**	**No. users**	**Beta**	***p* value**	**Medications used**	
c.310G>A (p.Val104Ile)^a^	AMR-like	1	0	8.70E−06	1.20E−05	2.14	−0.18	0.09	1.26	0.15	0.52	1	3.64	1.40E−10^b^	calcitriol, calcium carbonate, calcium citrate, cholecalciferol	epilepsy: inf, *0.02*^b^; hypoparathyroidism: inf, 2.6E−03^b^
	EUR-like	1	0	8.70E−06	3.90E−06	2.07	−0.23	*0.02*	N/A	N/A	N/A	0	N/A	N/A	N/A	hypocalcemia: inf, 0.01^b^
c.452C>T (p.Thr151Met)^a^	AMR-like	1	0	4.30E−06	1.20E−05	1.66	−0.65	3.20E−09^b^	1.42	0.29	0.21	1	0.55	0.34	calcium carbonate	N/A
c.613C>T (p.Arg205Cys)	AFR-like	4	0	3.00E−05	3.90E−05	2.45	0.13	0.09	1.02	−0.1	0.49	1	0.05	0.85	cholecalciferol	N/A
	EAS-like	3	0	3.00E−05	2.70E−04	2.4	0.08	0.46	N/A	N/A	N/A	1	0.23	0.42	cholecalciferol	N/A
c.1810G>A (p.Glu604Lys)	EUR-like	1	0	4.30E−06	3.90E−06	2.45	0.12	0.23	N/A	N/A	N/A	0	N/A	N/A	N/A	N/A
c.2431A>G (p.Met811Val)^a^	EAS-like	1	0	4.30E−06	9.00E−05	2.07	−0.23	0.03^b^	1.35	0.22	0.32	1	1.58	1.10E−03^b^	calcium carbonate, cholecalciferol	hypocalcemia: inf, 8.1e−03^b^
c.2443_2445del (p.Phe815del)^a^	EUR-like	2	0	8.70E−06	7.80E−06	2.03	−0.29	4.70E−03^b^	N/A	N/A	N/A	0	N/A	N/A	N/A	N/A
c.2503G>A (p.Ala835Thr)^a^	EUR-like	1	0	4.30E−06	3.90E−06	1.97	−0.34	1.10E−03^b^	1.37	0.28	0.15	1	4.49	1.90E−11^b^	calcitriol, calcium carbonate, calcium citrate, cholecalciferol, teriparatide	abnormal involuntary movements: inf, 0.03^b^; disorders of calcium/phosphorus metabolism: inf, 7.5e−03^b^; epilepsy: inf, 0.03^b^; hypocalcemia: inf, 0.01^b^; hypoparathyroidism: inf, 3.7e−03^b^
c.2800C>T (p.Gln934Ter)^a^	AFR-like	1	0	4.30E−06	9.80E−06	1.97	−0.36	7.20E−04^b^	N/A	N/A	N/A	0	N/A	N/A	N/A	N/A

Bonferroni significance threshold was 5.0E−3 for calcium and diagnoses, 1E−2 for phosphate, and 8.3E−3 for medications. Lower limit of normal (LLN) calcium is 2.2 mM, and upper limit of normal (ULN) phosphate is 1.45 mM. AFR, African ancestry; AMR, American ancestry; EAS, East Asian ancestry; EUR, European ancestry; het, heterozygous; hom, homozygous; MAF, minor-allele frequency.

a At least one nominally significant association.

b *p* < 0.05.

We detected no associations with ADH1 phenotypes in individuals with Arg205 in either the UKB or AOU. Interestingly, c.613C>T (p.Arg205Cys) is reported in ClinVar,^43^ as observed in both individuals with FHH1^44^ and ADH1^29^ with no functional analyses listed. Therefore, this variant was excluded from the known ADH1 variants list used for the following prevalence and penetrance estimates, as well as for developing the ADH1 score.

### Frequency and symptom burden due to *CASR* variants previously associated with ADH1 in the UKB, AOU, TOPMed, and gnomAD

In the UKB, the frequency of previously established ADH1-associated variants was 2.4:100,000. In AOU, the frequency was 3.9:100,000. Similarly, five previously established variants were observed (c.372C>A [p.Asn124Lys], c.452C>T [p.Thr151Met], c.1767C>G [p.Phe589Leu], c.2530G>A [p.Ala844Thr], and c.2647G>A [p.Val883Met]) in TOPMed^45^ with a frequency of 3.8:100,000, and five (c.310G>A [p.Val104Ile], c.1767C>G [p.Phe589Leu], c.2330T>C [p.Ile777Thr], c.2530G>A [p.Ala844Thr], and c.2647G>A [p.Val883Met]) were observed in gnomAD^46^ with a frequency of 4.6:100,000. These frequencies were similar to a previously reported^9^ frequency of 3.9:100,000 within the Geisinger DiscovEHR cohort.

Although these variants are pathogenic for ADH1, their symptom burden can be variable. Clinically, ADH1 is heterogeneous, with only 73% of diagnosed individuals experiencing hypocalcemia-related symptoms.^4^ In the UKB, among individuals with both in-patient and primary care records, we found that 1/6 (17%) heterozygotes had an ICD-10 code directly suggestive of diagnosis of ADH1 (hypocalcemia, disorder of calcium/phosphorous metabolism, or hypoparathyroidism), despite the high frequency of hypocalcemia observed based on calcium measurements, with 6/10 (60%) heterozygotes at or below the serum calcium LLN. Within AOU, 4/9 (44%) had a diagnosis directly suggestive of ADH1. The lower rate of diagnoses in both cohorts could suggest underdiagnosis or incomplete coverage of medical history.

Although the UKB and AOU may trend healthier than the general population, we observed that individuals with ADH1 are well captured in both cohorts. This gave us confidence that these two cohorts could be mined to identify additional ADH1 variants.

### Development of a variant score to identify hypocalcemia-associated variants in the UKB and AOU

**Figure 1:**
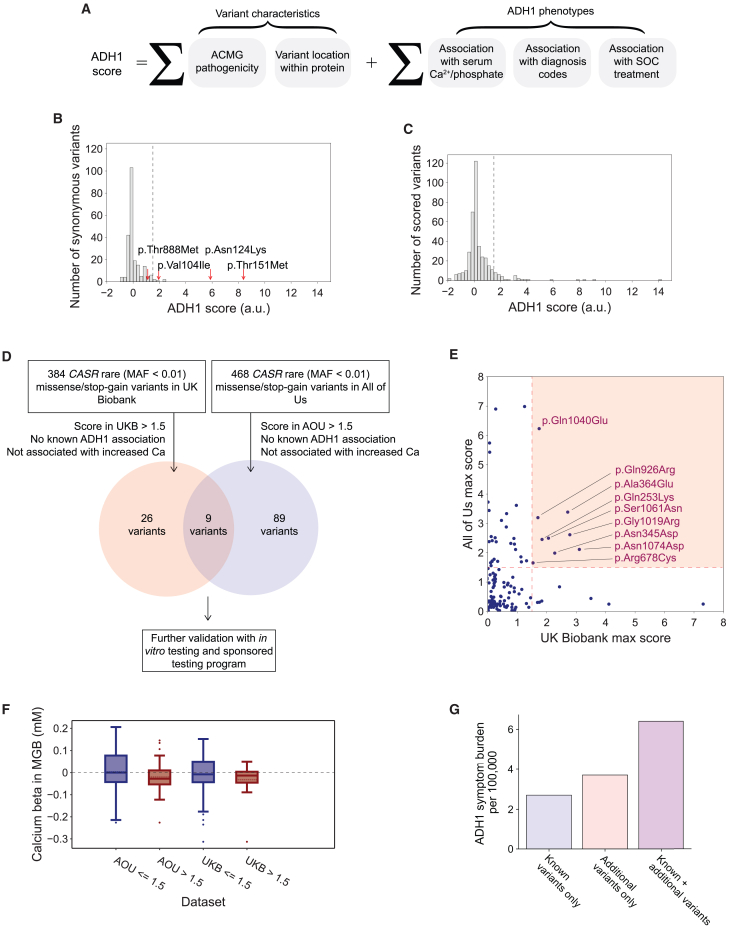
A scoring method to identify variants associated with ADH1 (A) The ADH1 score for each variant and each self-reported ethnicity in the UKB or genetically determined ancestry in AOU was calculated as the weighted sum of sub-scores corresponding to strength of association with ADH1 phenotypes and other variant characteristics. Variant location had a positive sub-score when variants were within known hotspots of GoF variants (aa positions 116–136 and 819–837). SOC treatment, standard-of-care treatment, e.g., calcium supplementation or parathyroid hormone. (B) Comparison of synonymous variants (bars) and previously described ADH1-associated variants (red arrows) shows separation of their scores. (C) Distribution of all scored variants (missense, frameshift, or nonsense). (D) Summary of biobank scoring analysis. (E) Comparison of ADH1 scores of variants found in both the UKB and AOU. Highlighted region indicates variants that scored higher than the 1.5 threshold in both the UKB and AOU. (F) Boxplot comparing effects on serum calcium, estimated in the MGB Biobank, of variants that scored above/below the threshold of 1.5 in AOU and the UKB. The boxes represent the interquartile range (IQR), the whiskers extend to 1.5 times the IQR, and the dots are outside of this range. The dashed line indicates the mean. (G) Bar chart showing the symptom burden of ADH1 (per 100,000 individuals in the UKB) from known variants, additional variants, and the combined total. ADH1, autosomal dominant hypocalcemia type 1; AOU, All of Us; a.u., arbitrary units; MGB, Mass General Brigham; SOC, standard of care; UKB, UK Biobank.

Because ADH1 was only recently assigned a specific ICD-10 code (E20.810) and due to the incomplete penetrance and variable expressivity we observed across known ADH1-associated variants, it was unlikely that a single phenotype could identify individuals with ADH1. Therefore, we undertook a holistic evaluation of known ADH1 phenotypes in addition to other variant characteristics that indicate GoFs. We developed a variant score, referred to as the ADH1 score, within the UKB cohort that corresponded to the strength of a variant’s association with ADH1 phenotypes and relevant variant characteristics (Figures 1A and S1).

The ADH1 score effectively distinguished synonymous variants from ADH1-associated variants in the UKB self-reported White British cohort (0.02 vs. 5.14, *p* = 2.2E−33; Figure 1B). Based on an empirical distribution generated by resampling sub-scores, a threshold of 1.5 was selected, yielding a specificity of 0.98 and a sensitivity of 0.75 in the same cohort, with similar performance observed across other self-reported ethnicities (Tables S3 and S4).

The ADH1 score was applied to 384 rare (minor-allele frequency [MAF] < 0.01) missense/nonsense/frameshift *CASR* variants across the UKB and 468 such variants in AOU.^4^^,^^5^^,^^6^ A summary of our analysis is shown in Figure 1D, and a complete table of variants and their scores is provided in Tables S7 and S8. Excluding previously established ADH1- and FHH1-associated variants, 35 variants had a score above 1.5 (Figure 1C) in the UKB. Within AOU, 98 variants had a score above 1.5. Overall, we found that 124 variants scored above the threshold in either the UKB or AOU (Figures 1D, 1E, and S4). There was relatively limited overlap since only 151 rare missense/frameshift/stop gains were detected in both biobanks.

### Validating the ADH1 score in the MGB Biobank

898 *CASR* variants were identified in the MGB Biobank (*n* = 39,081). 101 and 112 were rare (MAF < 0.01) missense/stop-gain variants that were also found in the UKB and AOU, respectively. For each of these variants, we estimated the effect on serum calcium and stratified the variants based on the ADH1 score computed in the UKB and AOU, respectively. The median effect on serum calcium was more negative for variants with ADH1 scores >1.5 than those with ADH1 scores ≤1.5, which was statistically significant for the AOU-based score and directionally consistent for the UKB-based score, providing independent evidence for the utility of the ADH1 score (t test; AOU: *p* = 4.6E−2 and UKB: *p* = 1.3E−1; Figure 1F). Additionally, the majority of variants had the same effect direction when present in the MGB Biobank (Figure S5; Table S9).

### Identification of nine additional variants with ADH1 associations in both the UKB and AOU

There were nine variants that scored >1.5 in both biobanks (Tables 3 and S10; a systematic review of the location and structural modeling of the variants is provided in Table S5, and a structural analysis of the variants is shown in Figure S2). c.757C>A (p.Gln253Lys) was associated with relevant medications in the UKB meta-analysis (*p* = 6.70E−06), nominally associated with partial epilepsy (*p* = 0.02), and suggestively associated with disturbance of skin sensation (*p* = 0.08) in the AOU AMR-like cohort. c.1033A>G (p.Asn345Asp) was associated with relevant medications in the UKB meta-analysis (*p* = 1.40E−67) and tetany in the UKB self-reported Black African cohort (*p* = 3.5E−03). It was also nominally associated with partial epilepsy in the AOU AFR-like cohort (*p* = 0.04). c.1091C>A (p.Ala364Glu) was associated with mildly reduced serum calcium levels in the UKB self-reported White British cohort (*p* = 1.20E−04) and nominally associated with hypoparathyroidism (*p* = 0.05) and suggestively associated with disorders of calcium/phosphorus metabolism (*p* = 0.10) within the AOU EUR-like cohort.

**Table 3:** Variants that scored above threshold in both UKB and AOU

**Variant**	**Biobank**	**Ethnicity or Ancestry**	**No. het**	**No. hom**	**MAF in ethnicity or ancestry**	**ADH1 score**	**Calcium**			**Phosphate**			**Meds**	
							**Mean (mM)**	**Beta (mM)**	***p* value**	**Mean (mM)**	**Beta (mM)**	***p* value**	**Beta**	***p* value**
c.757C>A (p.Gln253Lys)	UKB	Black African	3	0	1.80E−05	−0.02	2.38	−8.10E−03	0.91	1.18	0.03	0.81	−0.01	0.85
	UKB	Caribbean	10	0	1.80E−05	1.84	2.33	−0.06	0.18	1.23	0.07	0.31	−0.02	0.6
	AOU	AFR-like	40	0	3.90E−04	−0.15	2.3	−0.02	0.51	1.08	−0.03	0.7	0.05	0.58
	AOU	AMR-like	8	0	9.80E−05	2.45	2.39	0.07	0.1	0.9	−0.21	0.37	0.14	0.48
c.1033A>G (p.Asn345Asp)	UKB	Black African	1	0	1.10E−06	2.27	2.53	0.14	0.16	1.17	−9.30E−04	1	−0.03	0.78
	AOU	AFR-like	8	0	7.80E−05	0.95	2.27	−0.04	0.35	1.06	−0.05	0.64	−0.07	0.72
	AOU	EUR-like	4	0	1.60E−05	1.99	2.18^a^	−0.13	0.09	1.05	−0.01	0.92	−0.2	0.55
c.1091C>A (p.Ala364Glu)	UKB	White British	114	0	1.30E−04	2.71	2.34	−0.04	1.20E−04^b^	1.18	0.02	0.16	−0.02	0.18
	AOU	EUR-like	14	0	5.50E−05	3.39	2.33	0.02	0.53	1.28	0.19	0.16	0.05	0.76
c.2032C>T (p.Arg678Cys)	UKB	White British	3	0	5.30E−06	1.53	2.46	0.08	0.14	1.19	0.06	0.51	−0.02	0.84
	AOU	AFR-like	3	0	2.90E−05	1.65	2.32	0.02	0.88	1.19	0.04	0.83	N/A	N/A
	AOU	EUR-like	3	0	1.20E−05	0.19	2.19^a^	−0.12	0.09	0.82	−0.24	0.22	0.2	0.61
c.2777A>G (p.Gln926Arg)	UKB	White British	76	0	9.20E−05	1.7	2.35	−0.03	0.03^b^	1.2	0.06	2.40E−03^b^	−2.20E−03	0.89
	UKB	Indian	2	0	9.20E−05	0.11	2.4	N/A	N/A	1.08	N/A	N/A	−2.50E−16	5.40E−04^b^
	AOU	AFR-like	1	0	9.80E−06	2.23	2.38	0.06	0.58	1.44	0.34	0.11	1.62	3.00E−03^b^
	AOU	AMR-like	5	0	6.10E−05	3.18	2.17^a^	−0.12	0.06	1.41	0.32	0.17	−0.04	0.88
	AOU	EUR-like	58	0	2.30E−04	3.2	2.28	−0.03	0.12	1.13	0.05	0.25	0.25	4.50E−03^b^
c.3055G>A (p.Gly1019Arg)	UKB	Caribbean	3	0	1.70E−05	2.78	2.27	−0.13	0.08	0.93	−0.21	0.07	−0.02	0.8
	AOU	White British	12	0	1.70E−05	0.85	2.4	0.01	0.65	1.14	−0.04	0.41	−0.02	0.6
	AOU	AFR-like	7	0	6.80E−05	2.61	2.32	0.01	0.84	1.57^c^	0.46	0.03^b^	N/A	N/A
	AOU	EUR-like	3	0	1.20E−05	1.23	2.27	−0.05	0.51	1.09	0.05	0.72	0.33	0.39
c.3118C>G (p.Gln1040Glu)	UKB	Caribbean	2	0	2.10E−06	1.74	2.46	0.07	0.48	1.35	0.23	0.15	−9.00E−03	0.92
	AOU	AFR-like	2	0	2.00E−05	6.23	2.31	1.00E−03	0.99	1.3	0.2	0.34	N/A	N/A
	AOU	AMR-like	1	0	1.20E−05	−0.02	2.38	0.07	0.55	N/A	N/A	N/A	N/A	N/A
c.3182G>A (p.Ser1061Asn)	UKB	White British	1	0	1.10E−06	2.06	N/A	N/A	N/A	N/A	N/A	N/A	−0.03	0.8
	AOU	SAS-like	1	0	1.60E−04	2.5	2.32	0.02	0.8	N/A	N/A	N/A	1.71	4.40E−04^b^
c.3220A>G (p.Asn1074Asp)	UKB	Black African	3	0	7.80E−05	3.11	2.32	−0.02	0.8	1.26	0.08	0.51	−0.03	0.63
	UKB	White British	58	0	7.80E−05	3.01	2.36	−0.02	0.12	1.17	0.01	0.62	1.80E−03	0.92
	AOU	AFR-like	3	0	2.90E−05	2.11	2.13^a^	−0.16	0.15	1.23	0.1	0.63	N/A	N/A
	AOU	AMR-like	1	0	1.20E−05	0.57	2.32	0.04	0.75	N/A	N/A	N/A	N/A	N/A
	AOU	EUR-like	7	0	2.70E−05	1.93	2.24	−0.07	0.24	0.84	−0.21	0.28	−0.09	0.72
	AOU	MID-like	1	0	5.50E−04	1.33	2.16^a^	−0.14	0.19	1.21	0.2	0.32	0.43	0.47

In the UKB, Bonferroni significance thresholds were 7.1E−3 for calcium and phosphate and 6.2E−3 for medications. In AOU, Bonferroni significance threshold was 5.0E−3 for calcium and diagnoses, 1E−2 for phosphate, and 8.3E−3 for medications. Lower limit of normal (LLN) calcium is 2.2 mM, and upper limit of normal (ULN) phosphate is 1.45 mM. c.1091C>A (p.Ala364Glu) had an *in vitro* assay *p* < 5E−2 and c.3220A>G (p.Asn1074Asp) had an *in vitro* assay *p* = 7E−2, and both variants were detected in the sponsored testing program. The ethnicity or ancestry column contains self-reported ethnicities for the UKB and genetically determined ancestry for AOU. ADH1, autosomal dominant hypocalcemia; AFR, African ancestry; AMR, American ancestry; EAS, East Asian ancestry; EUR, European ancestry; het, heterozygous; hom, homozygous; MAF, minor-allele frequency; meds, medications; MID, Middle Eastern ancestry.

a Below LLN.

b *p* < 0.05.

c Above ULN

c.2032C>T (p.Arg678Cys) was nominally associated with paresthesia in the UKB self-reported White British cohort (*p* = 0.03) and abnormal involuntary movements in the AOU AFR-like cohort (*p* = 0.04). c.2777A>G (p.Gln926Arg) was associated with a mild decrease of serum calcium (*p* = 0.03) and a mild increase of serum phosphate (2.40E−03) in the UKB self-reported White British cohort. It was also associated with relevant medications across the UKB self-reported Indian cohort (*p* = 5.40E−04) and two ancestries in AOU (AFR-like, *p* = 3.00E−03, and EUR-like, *p* = 4.50E−03) and was associated with paresthesia in the UKB self-reported White British cohort (*p* = 0.05). It was suggestively associated with hypocalcemia in the AOU AFR-like cohort (*p* = 0.07) and with abnormal involuntary movements (*p* = 0.05) and generalized convulsive epilepsy (*p* = 0.09) in the AOU EUR-like cohort. c.3055G>A (p.Gly1019Arg) was nominally associated with disorders of calcium or phosphorous metabolism (*p* = 0.03) in the UKB self-reported Caribbean cohort as well as with chronic kidney disease in the UKB self-reported White British cohort (*p* = 0.01). In AOU, it was nominally associated with increased serum phosphate in the AOU AFR-like cohort (*p* = 0.03) and suggestively associated with abnormal involuntary movements (*p* = 0.10).

c.3118C>G (p.Gln1040Glu) was nominally associated with diagnosis of disorder of calcium or phosphorous metabolism in the UKB self-reported Caribbean cohort (*p* = 0.02), and it was also nominally associated with abnormal involuntary movements (*p* = 0.03) and disorders of calcium/phosphorus metabolism (*p* = 6.4E−03) and suggestively associated with epilepsy (*p* = 0.06) and epilepsy, recurrent seizures, and convulsions (*p* = 0.05) in the AOU AFR-like cohort. c.3182G>A (p.Ser1061Asn) was nominally associated with disorders of calcium or phosphorous in the UKB White British cohort (*p* = 9.6E−03) and was associated with relevant medications (*p* = 4.40E−04) and nominally associated with disturbance of skin sensation (*p* = 0.04) in the AOU SAS-like cohort.

c.3220A>G (p.Asn1074Asp) was associated with hypoparathyroidism in the UKB self-reported Black African cohort (*p* = 2.6E−03), nominally associated with hypoparathyroidism in the AOU EUR-like cohort (*p* = 0.03), and suggestively associated with epilepsy (*p* = 0.08) and epilepsy, recurrent seizures, and convulsions (*p* = 0.07) in the AOU AFR-like cohort (Tables 3 and S10).

**Figure 2:**
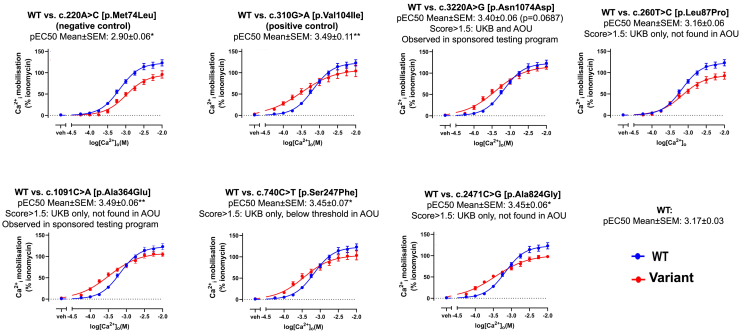
*In vitro* functional assay to characterize variant sensitivity to extracellular calcium HEK293 cells expressing each *CASR* variant were exposed to various concentrations of extracellular calcium, and calcium signaling activity was determined using Fluo-8 AM. c.220A>C (p.Met74Leu) was a negative control, and c.310G>A (p.Val104Ile) was a positive control. Each experiment was run 5 times. ^∗^*p* < 0.05 and ^∗∗^*p* < 0.01. *p* values are not reported for tests that are not statistically significant or near significance.

Validation of a subset of these variants was conducted through *in vitro* experiments testing for extracellular sensitivity to calcium (Figure 2; Table S11; supplemental methods). We exogenously expressed seven *CASR* variants (including a known GoF variant, c.310G>A [p.Val104Ile], and a LoF [FHH1] variant, c.220A>C [p.Met74Leu]) in HEK293 cells to determine their response to extracellular calcium. Of the five variants tested, two had ADH1 scores above the threshold in both the UKB and AOU (c.1091C>A [p.Ala364Glu] and c.3220A>G [p.Asn1074Asp]), and two only had ADH1 scores in the UKB (and were not found in AOU, c.2471C>G [p.Ala824Gly] and c.260T>C [p.Leu87Pro]), or did not score above the threshold in AOU, c.740C>T (p.Ser247Phe). Three of the five variants showed nominally significantly increased sensitivity to extracellular calcium (c.740C>T [p.Ser247Phe], c.1091C>A [p.Ala364Glu], and c.2471C>G [p.Ala824Gly], *p* < 0.05), and a fourth variant showed a suggestive increase in sensitivity (c.3220A>G [p.Asn1074Asp], *p* = 0.07). c.260T>C (p.Leu87Pro) showed no significant changes. Interestingly, c.3220A>G (p.Asn1074Asp) and c.1091C>A (p.Ala364Glu) were also observed in individuals enrolled in a genetic testing program for genetic hypoparathyroidism. Moreover, a pathogenic variant, likely pathogenic variant, or variant of uncertain significance was identified in *CASR* for more than half (56.3%) of individuals with suspected genetic hypoparathyroidism, comprising the largest genetic subset of the genes tested (see Table S12 and supplemental methods for a full list of variants).

### Frequency and disease burden of ADH1-associated variants

Interestingly, the nine variants identified using the ADH1 score were found at 12 times higher frequency than all previously established ADH1-associated variants combined (8.3E−5 vs. 6.9E−6; Tables 3, S10, and S13). These variants also had lower odds ratios for ADH1 phenotypes than previously established ADH1-associated variants (Tables 1 and 2).

To more accurately estimate the symptom burden due to these variants, we determined the excess number of individuals with any diagnosed ADH1 phenotype among individuals with an ADH1 variant in the UKB (Tables 3, S10, and S14). Across the nine variants, we found an excess of 14 individuals over the baseline with any ADH1 phenotype (3.7:100,000 in the UKB), which was similar to the frequency of previously reported variants in the UKB (2.7:100,000) (Figure 1G).

### Identification of putative phenotypes associated with GoF *CASR* variants

Because individuals with ADH1 have largely been ascertained based on the clinical phenotypes of individuals suspected to have genetic hypoparathyroidism, we sought to take a genetics-first approach to expanding the phenotypic spectrum of ADH1. A phenome-wide association study (pheWAS) through SKAT-O^34^ was conducted using both previously reported (*n* = 4) and additionally identified (*n* = 9) ADH1-associated variants in the White British cohort of the UKB across 1,518 phenotypes (Figure S6; Tables S15 and S16).

We recovered associations with abnormal reflex (*p* = 3.94E−27) and cardiac conduction disorder (*p* = 1.48E−18), both of which are linked to known symptoms of ADH1. Abnormal reflex may be related to tetany, and a prolonged QT interval has been reported in ADH1.^4^ Calcium plays an essential role in the heart,^47^ and hypocalcemia has generally been linked to QT prolongation^48^ and arrhythmia.^49^ Associations with coagulation defects and bone and tooth disorders were also detected, all of which have plausible connections to calcium homeostasis, as many of the proteins in the coagulation cascade are calcium dependent.^50^ Furthermore, other associations included rickets or osteomalacia (*p* = 7.34E−10), as well as hereditary disturbances in tooth structure (*p* = 6.26E−17). This was consistent with reported associations with mutations in the vitamin D receptor and hypocalcemic rickets^51^ and dental symptoms in persons with idiopathic hypoparathyroidism.^52^

### *CASR* variants constitute an allelic series on serum calcium

**Figure 3:**
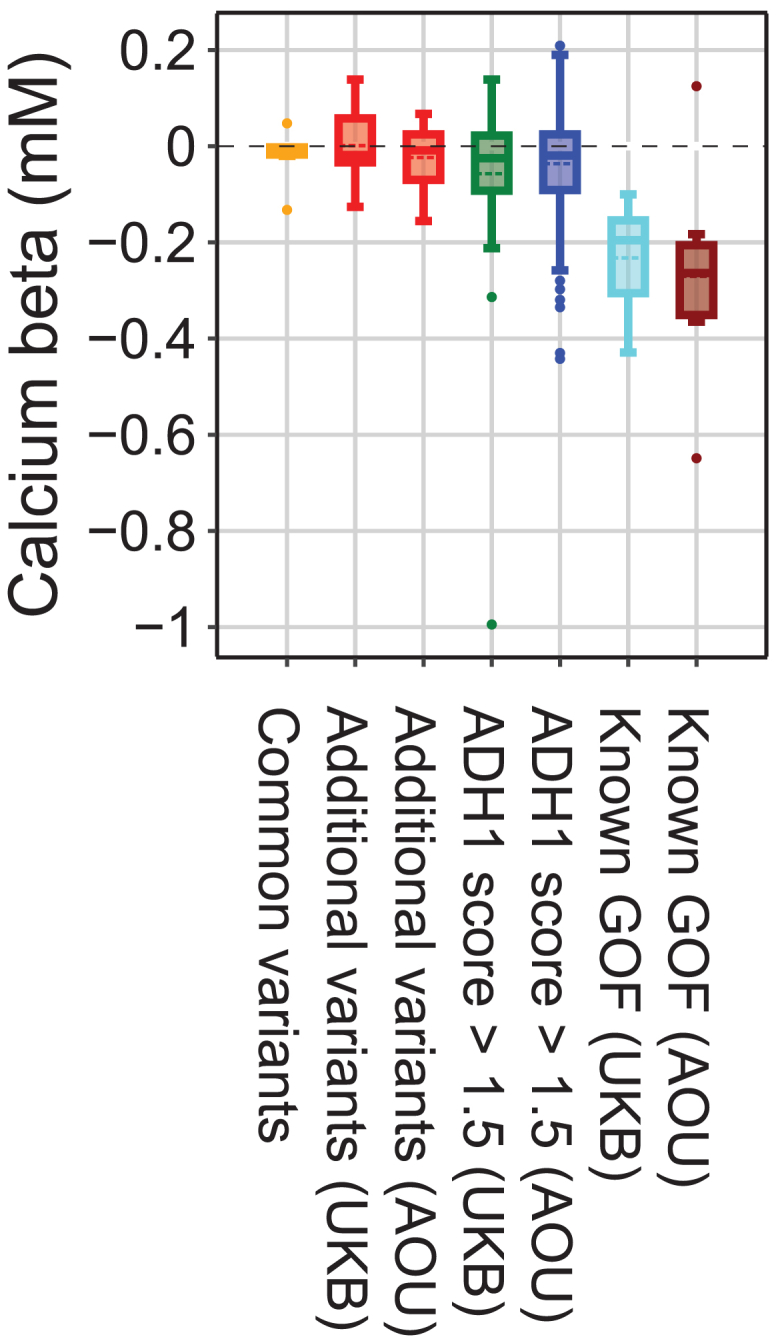
Genetic architecture of serum calcium with respect to *CASR* variation Boxplot of calcium effects for sets of variants. The boxes represent the interquartile range (IQR), the whiskers extend to 1.5 times the IQR, and the dots are outside of this range. Additional variants are those that scored >1.5 in both the UKB and AOU (as presented in Tables 3 and S10). The ADH1 score >1.5 categories include the additional variants as well as all others that met the score criteria for each biobank. ADH1, autosomal dominant hypocalcemia type 1; AOU, All of Us; IQR, interquartile range; UKB, UK Biobank.

Multiple independent common variants in *CASR* are also associated with serum calcium levels.^53^ We conducted fine mapping of these common variants using SuSiE^54^ and identified 10 independent causal sets that were distinct from ADH1-associated variants (Table S17). Additionally, we compared their frequency and effect size against the nine variants identified using the ADH1 score, as well as variants previously associated with ADH1 (Figures 3 and S7). As expected, we show that the impact on serum calcium grew more negative as the variant frequency decreased, suggesting *CASR* function as a central lever for calcium homeostasis in humans.

## Discussion

Through our analysis of two large-scale biobanks, we found that the symptom burden of ADH1 is at least 2-fold greater than previously believed.^9^^,^^55^ We developed an ADH1 variant score that identified five ADH1-associated variants with intermediate effect and frequency. The identified variants show a statistically significant but smaller effect on serum calcium, which can be explained by a combination of lower penetrance and variable expressivity. In other words, a larger proportion of individuals with these variants have sub-clinical phenotypes than traditional ADH1 variants, but individuals in the clinical range show overt clinical symptoms, making these variants more difficult to identify as ADH1 associated. These variants complete an allelic series of *CASR* by bridging the gap between common genome-wide association study (GWAS) variants and rare familial variants (a GoF causing hypocalcemia and a LoF causing hypercalcemia). This allelic series suggests that there is a dose-responsive relationship between *CASR* activity and serum calcium levels. However, we note that the pEC50 (negative logarithm of the EC50, which stands for half-maximal effective concentration) observed in *in vitro* assays did not correlate directly with the penetrance of each variant. The assay only captures one dimension of calcium receptor activity that can be modulated in different ways, and pEC50 is just one dimension of the assay itself, as shown in the complex shape of calcium mobilization. This effect would need to be considered in the context of other factors to quantitatively measure the biochemical activity of CaSR.

Individuals with previously reported variants were hypocalcemic (63% in the UKB and 89% in AOU), but less than half had a diagnosis code consistent with ADH1 (17% in the UKB and 44% in AOU), which are rates lower than previously described in clinically ascertained cohorts.^4^ In principle, our approach provides a complementary data point to the classical phenotype-first penetrance of ADH1 variants. Nevertheless, even based on this lower-bound penetrance, we suggest a symptomatic prevalence of 0.3:100,000–2.0:100,000, purely due to previously reported ADH1 variants, and the nine additional variants alone contribute to an additional symptom burden of 3.7:100,000. This figure only accounts for symptom burden due to variants observed in both biobanks and is likely still an underestimate of the true ADH1 symptom burden.

The higher-than-expected genetics-based prevalence of monogenic disorders is consistent with recent reports in *NOTCH3*-associated monogenic stroke (MIM: 125310),^56^ monogenic developmental disorders,^57^ and other dominant disorders,^58^^,^^59^ highlighting an important but underrecognized role of genetic variants underlying disease variability in the general population. These studies consistently showed an increased prevalence of dominant pathogenic variants but significantly smaller numbers of diagnosed individuals than expected, suggesting lower penetrance and greater variability in expressivity that may be modified by other genetic factors, environmental factors, or simply stochasticity in these seemingly monogenic disorders.^56^^,^^60^^,^^61^ Future work could consider environmental factors (e.g., diet) or a genome-wide or pathway-based polygenic score (e.g., incorporating variants in other genes involved in calcium homeostasis such as *VDR* [MIM: 601769], *CYP24A1* [MIM: 126065], *CLDN14* [MIM: 605608], *GNA11* [MIM: 139313], and *PTH1R* [MIM: 168468]^62^) to further understand modifiers of *CASR* variant penetrance.

The framework that we developed to identify novel ADH1 associations may serve as or could be developed further into a blueprint to use for other monogenic diseases. Our work highlights the potentially underrecognized symptom burden driven by these genes. However, even with a sample size of ∼750,000, the number of individuals with known ADH1 variants was limited (*n* < 30); thus, we were underpowered to build a hypothesis-free model. Through ever-growing cohorts with diverse inclusion criteria, we anticipate that further improvements to this framework will deepen our understanding of the true burden and variable expressivity of variants in genes associated with rare monogenic disorders.

## Acknowledgments

We thank all participants of the UKB, AOU, and MGB Biobank, as this study would not have been possible without their contributions. We also thank the NIH’s AOU Research Program, the UKB resource (under application number 62375), and the MGB team for making available the participant data examined in this study. J.B.C. thanks K. Shen for her support and feedback. T.M.J. is a 10.13039/501100000925National Health and Medical Research Council Fellow (2008341). This work was funded by BridgeBio Pharma.

## Author contributions

Conceptualization, J.B.C. and S.-G.J.; formal analysis, J.B.C., M.M.S., A.P., C.P.B., A.M.A., B.W.D., J.H., Q.C., J.L.N., X.Z., and C.M.G.; investigation, J.B.C., M.M.S., A.P., C.P.B., A.M.A., B.W.D., J.H., L.M.S.S., A.S.M., J.L.N., J.D., C.D.F., Q.C., T.M.J., and C.M.G.; resources, L.M.S.S., A.S.M., and S.-G.J.; data curation, J.B.C., M.M.S., A.P., X.Z., and C.P.B.; writing – original draft, J.B.C. and S.-G.J.; writing – review & editing, C.L., R.A., T.M.J., J.A.L.-S., C.M.G., M.S.R., S.H.A., J.C.F., J.L.N., X.Z., J.B.C., and S.-G.J.; visualization, J.B.C.; supervision, J.B.C. and S.-G.J.; project administration, J.B.C. and S.-G.J.

## Declaration of interests

J.B.C., M.M.S., X.Z., C.P.B., J.L.N., L.M.S.S., A.S.M., A.P., M.S.R., S.H.A., J.C.F., and S.-G.J. are current or former employees and shareholders of BridgeBio Pharma. B.W.D., C.L., and R.A. are consultants of BridgeBio Pharma.

## Web resources

Coloc SuSiE, https://chr1swallace.github.io/coloc/articles/a06_SuSiE.html
GenBank, https://www.ncbi.nlm.nih.gov/genbank/
Genotype-Tissue Expression (GTEx) Project, https://www.gtexportal.org/home/
HAIL v.0.2.78, https://github.com/hail-is/hail
OMIM, https://www.omim.org
Owlready2, https://owlready2.readthedocs.io/en/latest/index.html#
Phecode Map 1.2, https://phewascatalog.org/phewas/#home
PLINK v.2.00a3.1LM, www.cog-genomics.org/plink/2.0/
Python statsmodels package v.0.14.1, https://www.statsmodels.org/stable/install.html
SKAT R package v.2.2.5, https://cran.r-project.org/web/packages/SKAT/vignettes/SKAT.pdf
Varsome, https://varsome.com/

## Supplemental information

Data S1. Tables S1, S2, S5, S7–S10, S15, and S16

Document S2. Article plus supplemental information

## Footnotes

Supplemental information can be found online at https://doi.org/10.1016/j.ajhg.2025.06.013.
