# Supplementary material for: A calcium-sensing receptor allelic series and underdiagnosis of genetically driven hypocalcemia
Source: Am J Hum Genet. 2025 Jul 14;112(8):1818–32. doi: 10.1016/j.ajhg.2025.06.013 (PMC12414667; doi:10.1016/j.ajhg.2025.06.013)
Supplement: Document S1. Figures S1–S7, Tables S3, S4, S6, S11–S14 and S17, and supplemental methods [file mmc1.pdf]

The American Journal of Human Genetics, Volume *112*

Supplemental information

A calcium-sensing receptor allelic series and underdiagnosis of genetically driven hypocalcemia

Jeremy B. Chang, Connor P. Barnhill, Alexander M. Apostolov, Marcus M. Soliai, Julian Hecker, Jovia L. Nierenberg, Lyndsay M. Stapleton Smith, Arun S. Mathew, Xue Zeng, Jiayin Diao, C. Dilanka Fernando, Qingwen Chen, Ben W. Dulken, Aleksandr Petukhov, Russ Altman, Tracy M. Josephs, Jessica A. Lasky-Su, Caroline M. Gorvin, Mary Scott Roberts, Scott H. Adler, Jonathan C. Fox, Christoph Lange, and Sun-Gou Ji

Figure S1: Distribution of resampled total scores

Figure S2: Structural analysis of CaSR variants

Figure S3: Serum calcium and phosphate levels in the UK Biobank

Figure S4: Maximum scores in the UKB and AOU

Figure S5: Comparison of serum calcium effects in MGB, AOU, and UKB

Figure S6 Phenome-wide association study using both known and novel ADH1-associated variants

Figure S7: Genetic architecture of serum calcium with respect to CASR variation

Table S3: Sensitivity and specificity in the UK Biobank

Table S4: Sensitivity and specificity in All of Us

Table S6: UKB known ADH1 variants outside of analysis cohort

Table S11: In vitro testing

Table S12: Sponsored testing variants

Table S13: Variant frequencies

Table S14: Symptomatic odds ratios

Table S17: Common variants

Supplemental Methods

References

Figure S1: Distribution of resampled total scores

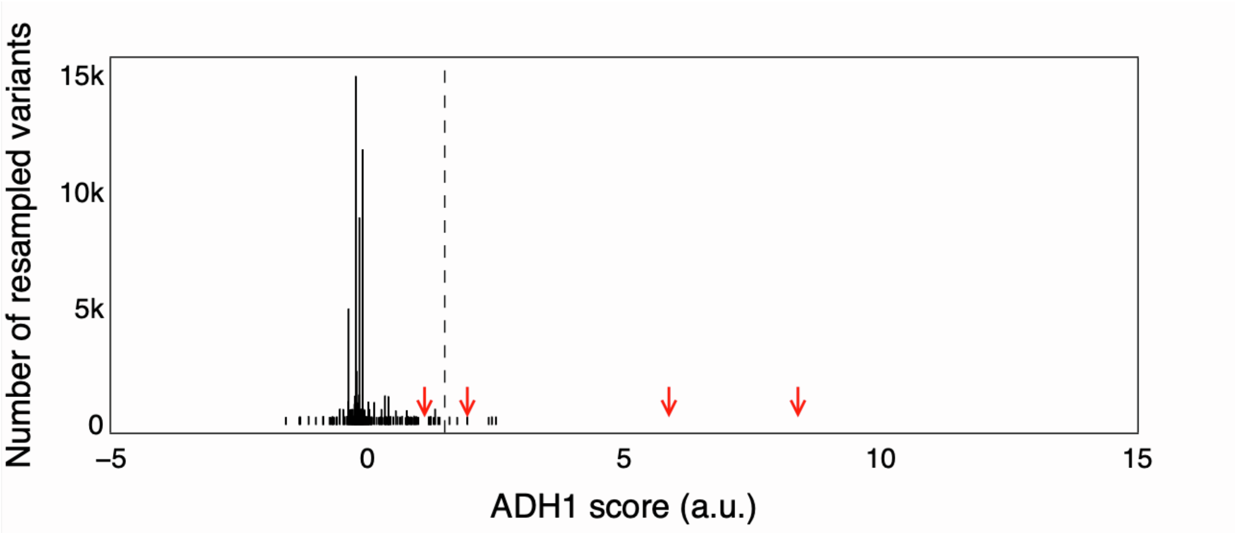

Figure caption: **Distribution of resampled total scores.** Histogram of total scores from the null distribution, which was created by re-sampling scores from synonymous variants. Red arrows indicate values of previously described gain-of-function variants. ADH1=autosomal dominant hypocalcemia type 1; a.u.=arbitrary units.

Figure S2: Structural analysis of CaSR variants

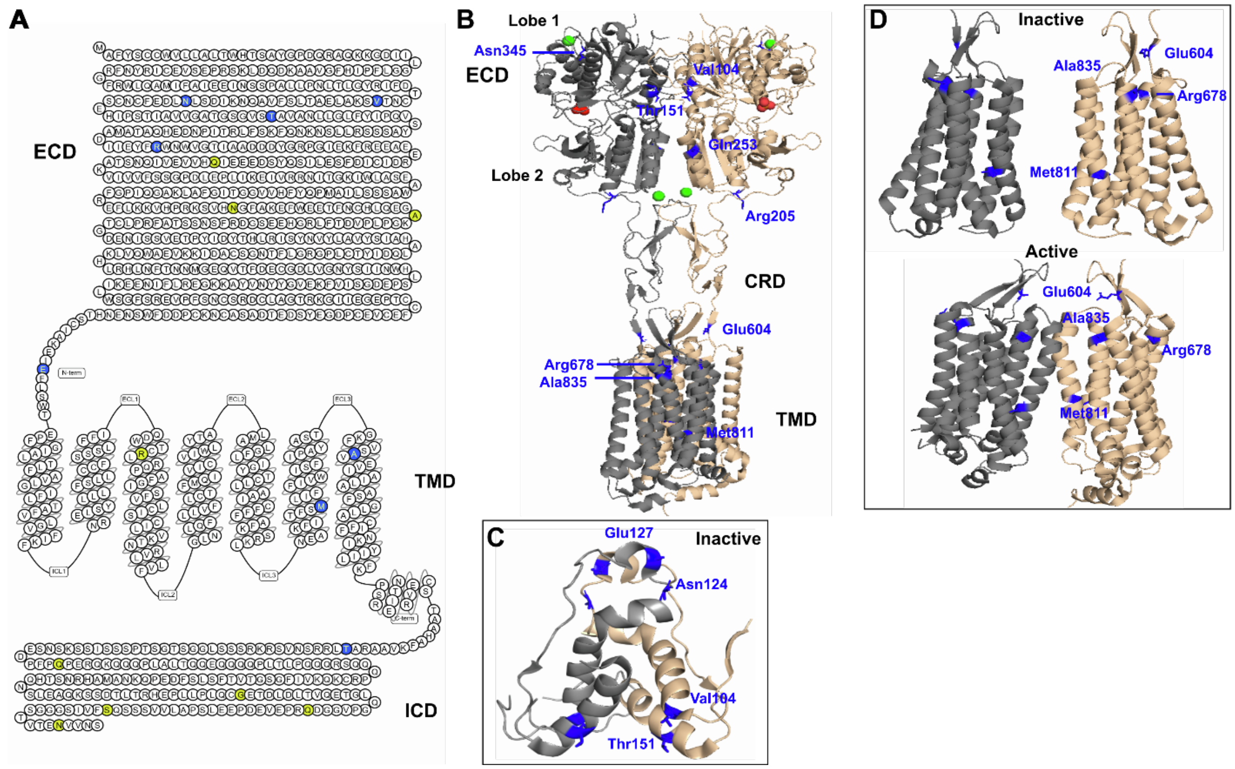

Figure caption. **Structural analysis of CaSR variants.** (A) Snake plot showing the locations of the 14 missense variants identified in Tables 1-3. Tables 1–2 missense variants (blue), Table 3 variants (yellow). ECD, extracellular domain, TMD, transmembrane domain, ICD, intracellular domain. (B) Model of homodimeric CaSR ECD, cysteine-rich domain (CRD) and TMD highlighting the locations of residues where variants were identified. Protomer 1 shown in grey and protomer 2 in light brown. Ca^{2+} shown in green. (C) Zoomed image of the ECD variants in the dimer interface. Several residues project into the dimer interface. (D) Location of the TMD variant residues in the inactive and active state.

Figure S3: Serum calcium and phosphate levels in the UK Biobank

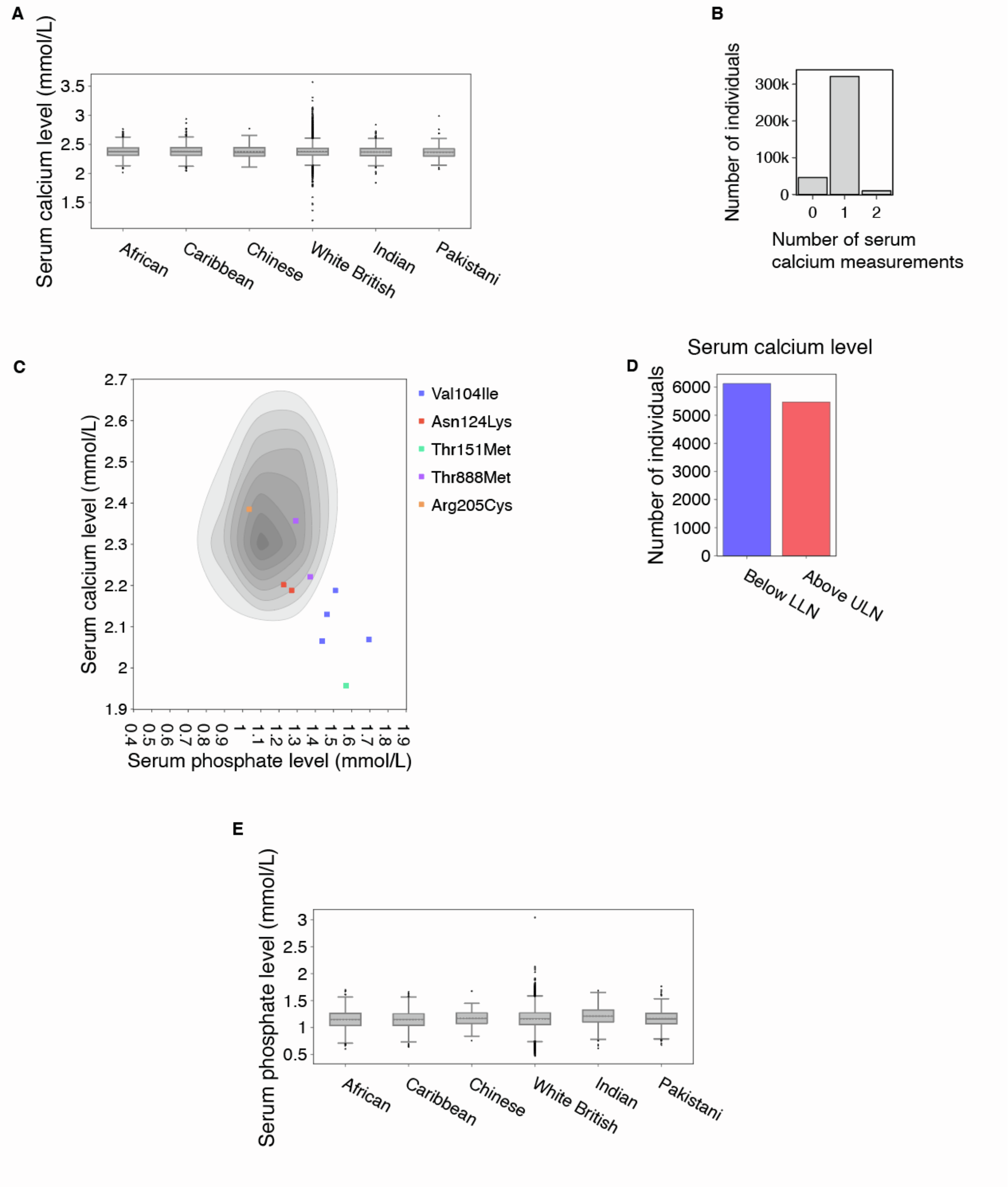

Figure caption. **Serum calcium and phosphate levels in the UK Biobank**. (A) Box plot showing serum calcium levels across self-reported ethnicities. *The boxes represent the interquartile range (IQR), the whiskers extend to 1.5 times the IQR. and the dots are outside of this range.* (B) Bar chart depicting the number of serum calcium measurements per individual in the UK Biobank, stratified by self-reported ethnicity. (C) Scatter plot showing the levels of serum calcium and serum phosphate for individuals with each variant. (D) Bar chart depicting the number of individuals with serum calcium below the lower limit of normal (LLN) or above the

upper limit of normal (ULN). (E) Box plot showing serum phosphate levels across self-reported ethnicities. *The boxes represent the interquartile range (IQR), the whiskers extend to 1.5 times the IQR. and the dots are outside of this range.*

Figure S4: Maximum scores in the UKB and AOU

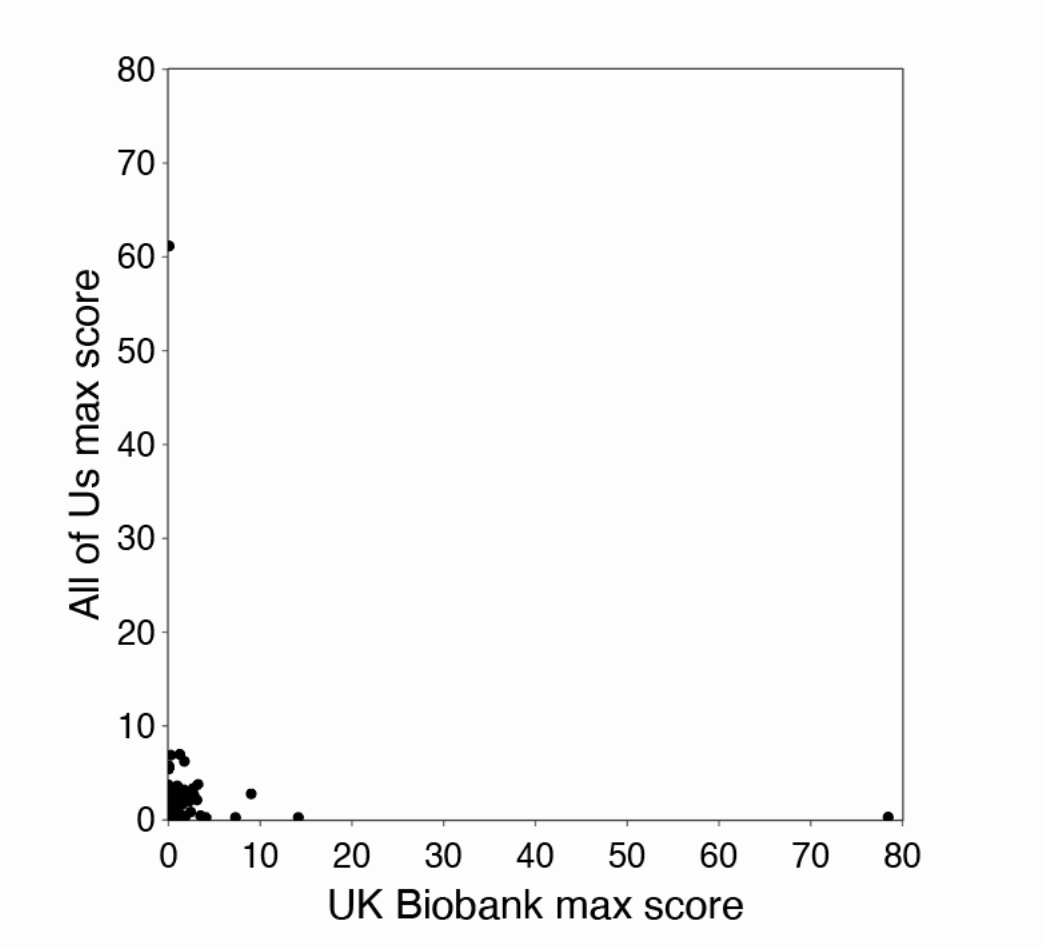

Figure caption: **Maximum scores in the UKB and AOU.** Scatter plot showing maximum scores in the UKB (on the x-axis) and AOU (on the y-axis). AOU=All of Us; UKB=UK Biobank.

Figure S5: Comparison of serum calcium effects in MGB, AOU, and UKB

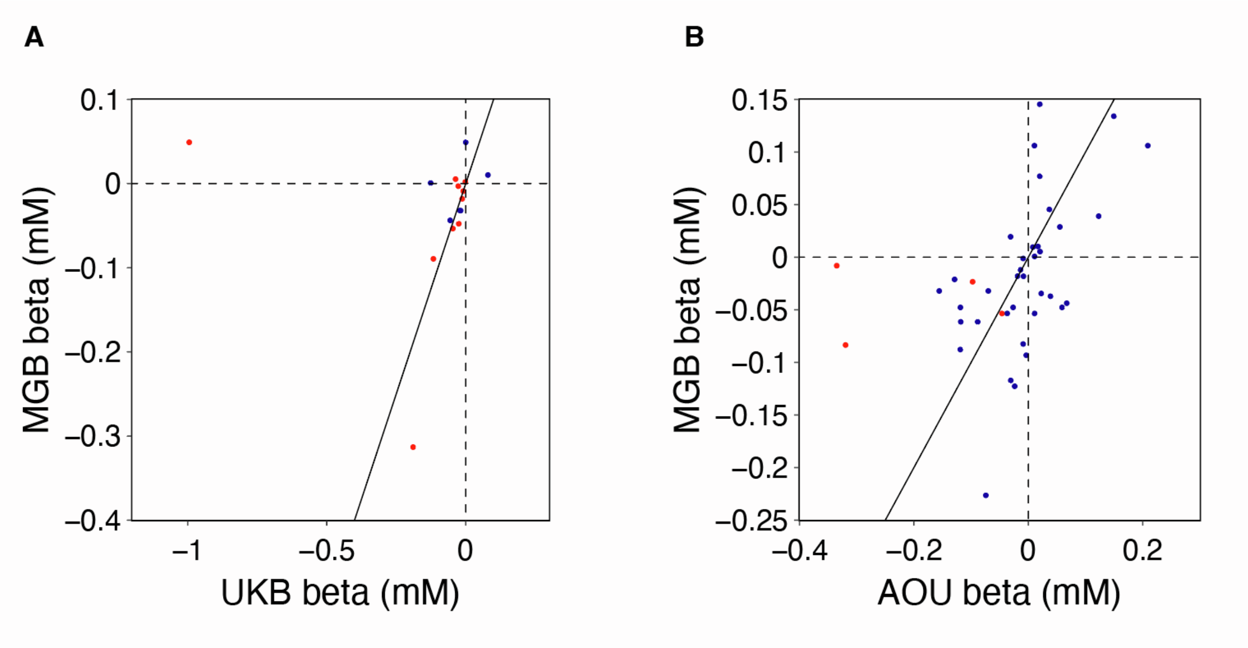

Figure caption: **Comparison of serum calcium effects in MGB, AOU, and UKB.** Scatter plot of betas of linear regression on serum calcium levels in the UKB (A) and AOU (B) compared with the MGB Biobank. All plots include only variants that have ADH1 score > 1.5. Red points correspond to variants that have a significant ($p < 0.05$) association with serum calcium in the UKB and AOU. The slope of the black line is 1. AOU=All of Us; MGB=Mass General Brigham; UKB=UK Biobank.

Figure S6 Phenome-wide association study using both known and novel ADH1-associated variants

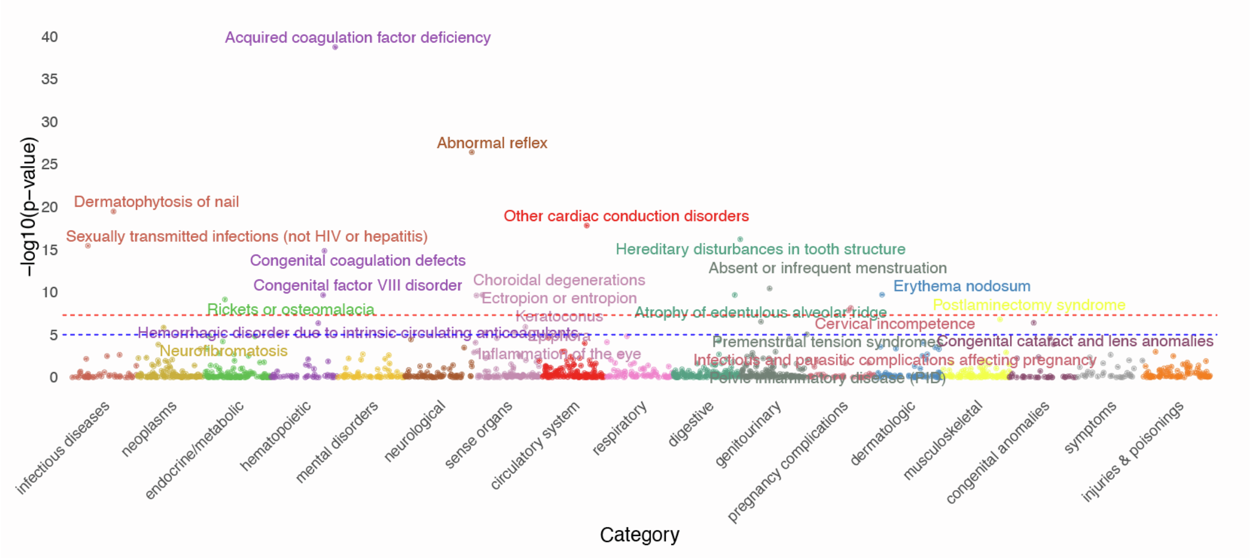

Figure caption: ***Phenome-wide association study using both known and novel ADH1-associated variants.*** *Blue dashed line indicates 1E-5 significance threshold, and red dashed line indicates 5E-8 significance threshold.*

Figure S7: Genetic architecture of serum calcium with respect to *CASR* variation

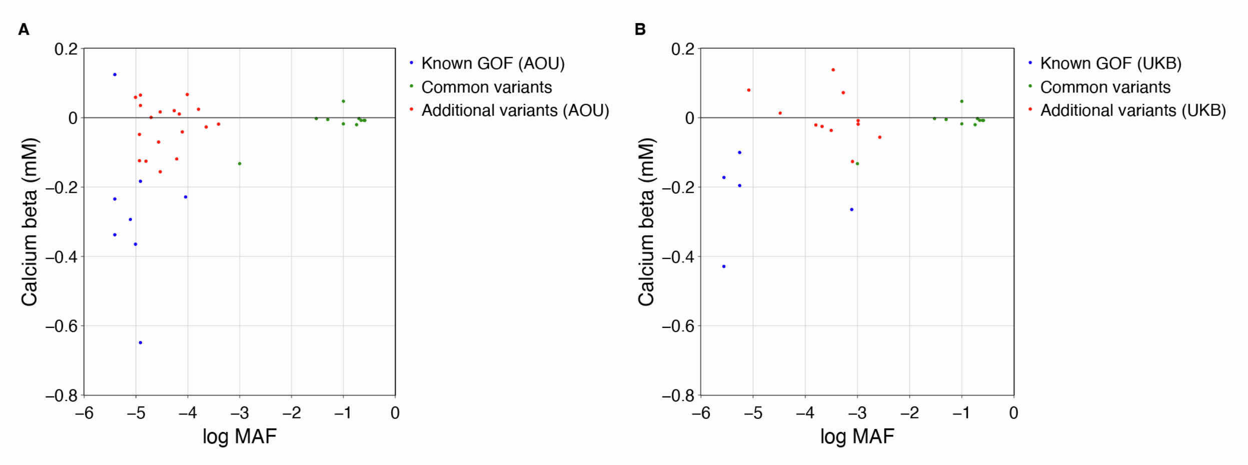

Figure caption: **Genetic architecture of serum calcium with respect to *CASR* variation.** (A, B) Scatter plot of effect on serum calcium versus $\log_{10}$ of variant minor allele frequency for various sets of variants in the UKB (A) or AOU (B).

Table S3: Sensitivity and specificity in the UK Biobank

Ethnicity Cohort	Sensitivity	Specificity
"African"	N/A	1
"Caribbean"	N/A	0.97
"Chinese"	N/A	1
"White British"	0.75	0.98
"Indian"	1	1
"Pakistani"	N/A	1

Table caption: ***Sensitivity and specificity in the UK Biobank***

Table S4: Sensitivity and specificity in All of Us

Ancestry Cohort	Sensitivity	Specificity
AFR-like	1	0.92
AMR-like	1	0.93
EAS-like	1	0.81
EUR-like	0.75	0.94
MID-like	N/A	0.88
SAS-like	N/A	0.94

Table caption: ***Sensitivity and specificity in All of Us.*** *AFR=African ancestry; AMR=American ancestry; EAS=East Asian ancestry; EUR=European ancestry; MID=Middle Eastern ancestry; SAS=South Asian ancestry.*

Table S6: UKB known ADH1 variants outside of analysis cohort

Variant	Ethnicity	Age	Sex	Ca2+ (mM)	Phosphate (mM)	Relevant meds	$eGFR_{cy}$ (mL/min/1.73m^2)	Diagnoses
p.Val104Ile [c.310G>A]	"Indian"	61	M	2.24	*1.6*	N/A	71.4	N/A
p.Glu127Gly [c.380A>G]	"British"	64	F	N/A	N/A	N/A	N/A	Chronic kidney disease, stage 3
p.Arg205Cys [c.613C>T]	"British"	43	F	2.46	1.05	N/A	122.1	N/A

*Table caption: **UKB known ADH1 variants outside of analysis cohort**. ADH1=autosomal dominant hypocalcemia type 1; Ca2+=calcium; $eGFR_{cy}$=estimated glomerular filtration rate calculated using cystatin C; F=female; M=male; meds=medications; UKB=UK Biobank.*

Table S11: In vitro testing

Variant	pEC50	SEM	n
WT	3.17	0.03	5
p.Met74Leu	2.90*	0.06	5
p.Val104Ile	3.49**	0.11	5
p.Leu87Pro	3.16	0.06	5
p.Ser247Phe	3.45*	0.07	5
p.Ala364Glu	3.49**	0.06	5
p.Ala824Gly	3.45*	0.06	5
p.Asn1074Asp	3.4	0.06	5
p.His766Gln	3.36	0.03	5

Table caption: ***In vitro testing.*** *Mean ± SEM. Log(agonist) vs. response, variable slope (four-parameter). Tested using one-way ANOVA with Dunnett's multiple comparison: *P < 0.05, **P < 0.01, **P < 0.001 vs. WT. p.Met74Leu is an FHH1 variant; p.Val104Ile is an ADH1 variant. ADH1 = autosomal dominant hypocalcemia type 1; FHH1 = familial hypocalciuric hypercalcemia type 1; N.D. = not determined; pEC*$_{50}$ *=* $-\log_{10}(EC_{50})$*, where* EC_{50} *is the concentration for half-maximal response; SEM = standard error of the mean; WT = wild type.*

Table S12: Sponsored testing variants

Variant
p.Leu723Arg
p.Asp275Glu
p.Ala364Glu
p.Arg896Alafs*43
p.Thr972Met
p.Pro278Leu
p.Ile777Thr
p.Cys129Tyr
p.Ile162Val
p.Phe790Leu
p.Asn1074Asp
p.Ile187Asn
p.Asn189His
p.Ala835Pro
p.Phe798Leu
p.Glu241Lys
p.Thr151Met
p.Glu604Lys
p.Phe788Cys
p.Ala840Val
p.Gln245Arg
p.Pro221Leu
p.Phe815del
p.Glu767Lys
p.Phe806Ser
p.Phe128Leu
p.Phe832Ser

Table caption: ***Sponsored testing variants***

Table S13: Variant frequencies

Variant	Population prevalence			
	UKB	AOU	gnomAD	TOPMed
p.Gln253Lys	1.72E-05	1.12E-04	2.97E-05	1.81E-04
p.Asn345Asp	1.07E-06	2.90E-05	4.34E-06	3.40E-05
p.Ala364Glu	1.27E-04	3.10E-05	8.18E-05	2.27E-05
p.Arg678Cys	5.37E-06	1.40E-05	7.44E-06	N/A
p.Gln926Arg	9.13E-05	1.35E-04	1.13E-04	1.17E-04
p.Gly1019Arg	1.72E-05	2.00E-05	1.98E-05	4.53E-05
p.Gln1040Glu	2.15E-06	8.00E-06	4.34E-06	1.13E-05
p.Ser1061Asn	1.07E-06	2.00E-06	6.84E-07	N/A
p.Asn1074Asp	7.63E-05	2.40E-05	5.64E-05	1.89E-05

*Table caption: **Variant frequencies.** AOU=All of Us; UKB=UK Biobank.*

Table S14: Symptomatic odds ratios

Variant	Ethnicity	Symptomatic odds ratio (p value)
p.Gln253Lys	“Caribbean”	1.9 (0.28)
p.Asn345Asp	“African”	12.5 (0.08)
p.Ala364Glu	“White British”	1.2 (0.61)
p.Arg678Cys	“White British”	4.0 (0.23)
p.Gln926Arg	“White British”	1.1 (0.68)
p.Gly1019Arg	“Caribbean”	3.2 (0.28)
	“White British”	4.0 (0.01)
p.Gln1040Glu	“Caribbean”	4.8 (0.2)
p.Ser1061Asn	“White British”	12.0 (0.08)
p.Asn1074Asp	“White British”	1.2 (0.48)
	“African”	4.2 (0.22)

Table caption: ***Symptomatic odds ratios***

Table S17: Common variants

SNPs (GRCh37)	MAF(s)	Beta (mg/dl)	Beta (mM)	P-value(s)	Annotation(s)
3.121981609.G.A	0.1	0.19	0.0475	7.49e-583	*CASR* intronic
3.122034854.A.G	0.25	-0.03	-0.0075	2.89E-29	Intergenic
3.122010221.C.T	0.001	-0.53	-0.1325	7.26E-54	*CASR* 3 prime UTR
3.122003769.A.G	0.18	-0.08	-0.02	3.63E-125	*CASR* missense
3.121945470.T.C, 3.121945838.A.G	0.26	-0.03	-0.0075	1.91e-25 - 1.90e-25	*CASR* intronic
3.122088753.C.T, 3.122102186.A.G	0.05	-0.02	-0.005	1.07e-05 - 1.54e-05	*MIX23*, *FAM162A* non-coding
3.121907476.A.G, 3.121922222.C.G	0.22	-0.03	-0.0075	1.20e-17 - 4.76e-18	*CASR* intronic
3.121975485.G.A, 3.121984021.G.A, 3.121987328.G.C, 3.121987587.G.C, 3.121988120.A.G, 3.121988505.T.C, 3.121988738.G.A, 3.121993432.G.A, 3.121996967.TTG.T, 3.122000667.C.T, 3.122001099.T.C, 3.122005131.C.T, 3.122010681.C.T	0.2	-0.01	-0.0025	1.92e-05 - 6.53e-05	*CASR* intronic, 3 prime UTR, or downstream transcript variant
3.121950668.A.G, 3.121951168.C.T	0.03-0.04	-0.01	-0.0025	1.87e-29 - 4.32e-28	*CASR* intronic

3.121845538.T.TTATACC, 3.121847759.C.T, 3.121854556.T.A, 3.121860038.A.G, 3.121864368.C.CT, 3.121867962.T.C, 3.121869044.A.C	0.10-0.13	-0.07	-0.0175	1.05e-86 - 8.81e-87	Intergenic

*Table caption: **Common variants**. Beta (mg/dl) = effect size in milligrams per deciliter; Beta (mM) = effect size in millimoles per liter; MAF = minor allele frequency; SNP = single nucleotide polymorphism (GRCh37 reference genome).*

Supplemental Methods

UK Biobank (UKB)

The UK Biobank (UKB) is a large-scale biomedical database and research resource containing genetic, lifestyle, health and clinical information from 500,000 participants[1]. The total number of participants with data in the UKB at time of analysis was 469,835. Informed consent was obtained from all study participants, and the study was approved by the North West Multi-Centre Research Ethics Committee as a Research Tissue Bank approval. Exome sequencing, alignment, quality control (QC), and variant calling of the UKB study participants were performed as previously described[2].

We performed QC on the UKB dataset with the goal of maximizing sensitivity for identifying novel GOF *CASR* (MIM: 601199) variants. QC was performed using the PLINK (v2.00a3.1LM)[3,4] and HAIL (v0.2.78)[5,6] software packages. Since autosomal dominant hypocalcemia type 1 (ADH1, MIM: 601198)-causing variants are expected to be rare, we aimed to retain as many samples, variants, and genotypes as possible. To this end, we employed a QC pipeline consisting of three steps: sample, variant, and genotype filtering. We applied these three steps in all possible orders and then took the union of all samples, variants, and genotypes that were retained after any order of application.

Before QC, the dataset consisted of 469,835 individuals, 1,258 variants, and 591,522,265 calls based on the MANE transcript of *CASR* (ENST00000639785.2, NM_000388.4). These variants were missense, synonymous, intron, 5'- and 3'-UTR, splicing, frameshift, stop-gain, in-frame indel, or impacted a non-coding transcript. For sample QC, we removed related individuals and those with missing call rate greater than 0.01. We also removed individuals with a disagreement between self-reported and genetic sex. For variant QC, we filtered variants with a call rate less than 0.9. For genotype QC, we applied different filters for SNVs and non-SNVs to ensure genotype quality. SNVs were filtered based on a genotype quality (GQ) score greater than 19 and a sequencing depth (DP) greater than 6, whereas non-SNPs were filtered based on a GQ greater than 19 and a DP greater than 9.

Following QC, the dataset comprised 433,793 individuals (age range: 37 to 73 years, 54% female). The number of “White British” (Field 22006) individuals was 392,147. This subset consists of participants with self-reported “White British” ethnicity determined to be genetically homogeneous by principal component analysis The remaining ethnicities were determined using self-reported ethnicity (Field 21000) with the following cohort sizes: Black African: 2,925, Any other Asian background: 1,614, Any other Black background: 105, Any other mixed background: 903, Any other white background: 14,705, Asian or Asian British: 40, Bangladeshi: 206, Black or Black British: 24, British: 382,216, Caribbean: 3,792, Chinese: 1,412, Do not know: 180, Indian: 5,179, Irish: 11,339, Mixed: 37, Other ethnic group: 4,012, Pakistani: 1,580, Prefer not to answer: 1,436, White: 474, White and Asian: 718, White and Black African: 363, White and Black Caribbean: 533. Self-reported ethnicity is used as a proxy for genetic ancestry because a true genetically homogeneous population is not provided in the UKB for ancestries other than "White British". We focused our analysis on the Black African, Caribbean, Chinese, White British, Indian, and Pakistani self-reported ethnicity groups due to cohort size (total of 407,755 individuals). We removed a total of 126 individuals across ethnicities who were outliers by principal components analysis (PCA)[7]. For each PC, ethnic outliers lying more than 6 SD away from the mean were excluded. There were 566,898,080 variant calls. In total, we identified 1,253 *CASR* variants relative to its MANE transcript (ENST00000639785.2/NM_000388.4; 1,078 amino acid residues).

Serum calcium levels were uncorrected for albumin based on studies showing a superior correlation between ionized calcium and uncorrected calcium values[8]. For both serum calcium levels and serum phosphate levels, association was tested using linear regression. Age, sex, and the first 10 genetic principal components were used as covariates.

eGFR was calculated using the 2012 CKD-EPI cystatin C equation[9] due to the lower missingness rate of cystatin C measurements relative to creatinine measurements in the UKB. Association was tested using linear regression. Age, sex, and the first 10 genetic principal components were used as covariates.

For medications, the count of instances of 'calcichew', 'calcichew d3 tablet', 'colecalciferol', 'cholecalciferol', 'calcitriol', 'calcium citrate', 'calcium carbonate', 'alfacalcidol', 'PTH', 'natpar', 'teriparatide', and 'forteo' was determined for each individual. Linear regression was used to determine the association of each variant with the medication count. Age, sex, and the first 10 genetic principal components were used as covariates.

We mapped ICD-10 codes to a total of 1,518 phenotypes (phecodes) using the Phecode Map 1.2 from phewascatalog.org[11]. To maximize sensitivity, we required only a single instance of a diagnosis for defining phenotypes. We applied the phecode exclusion ranges to remove controls with related conditions[10]. If an ICD diagnosis code did not match the ICD code in the phecode mapping directly, we removed the one or two rightmost digit(s) from the ICD code diagnosis and attempted to map again until the ICD codes were successfully mapped to phecodes.

For diagnosis codes, we considered phecodes 252.2 (Hypoparathyroidism), 275.5 (Disorders of calcium or phosphorous metabolism), 350.1 (Tetany), 687.4 (Paresthesia), and 345.0/345.1/345.11/345.12 (Epilepsy). Association was tested using Fisher's exact test and, for all locus-phenotype combinations for which there are at least 10 events per variable in the logistic regression[11–13], using logistic regression with Firth correction, following standard guidelines for logistic regression analysis. Based on the similarity of results from logistic regression with Firth correction and the Fisher exact test for combinations with more than 10 events per variable (**Table S1**), we concluded that the effects of the covariates/population substructure on the association are relatively minor and that, for locus-phenotype combination with less than 10 events per covariate, where the Fisher p-values are the only available association tests, the Fisher p-values provide meaningful information. Associations for all binary phenotypes were then tested using the Fisher exact test.

For individuals with previously established ADH1-associated variants, both inpatient and primary care data (in Read2 and CTV3 format) were examined. Analyses assessing the symptom burden of pathogenic variants were restricted to individuals with both inpatient and primary care data (n = 180,334).

All regression analyses were conducted separately in each ethnicity group, and results were meta-analyzed.

All of Us (AOU)
We used data from the AOU Research Program Curated Data Repository Release v7, a longitudinal cohort study led by the National Institutes of Health for the advancement of precision medicine[14]. These data included 245,394 short read whole genome sequences. 59% of individuals were female, and ages ranged from 18 to 117. We used the genomic data available on the AoU Research Bench that had already undergone quality control. We further filtered samples for those with call rate > 0.97, variants for call rate > 0.9, and genotypes for GQ > 19. To filter out related individuals, we used the precomputed maximal subset of unrelated samples that AOU provides. Genetic ancestry was used to group individuals.

For serum calcium level, we used the concept "Calcium [mass/volume] in Serum or Plasma", for which data was available for 143,416 individuals. For serum phosphate level, we used "Phosphate [mass/volume] in Serum or Plasma", for which data was available for 57,037 individuals. We retained measurements of calcium and phosphate levels between the 0.001 (calcium: 1.51 mmol/L, phosphate: 1.30 mg/dL) and 0.999 quantiles (calcium: 2.75 mmol/L, phosphate: 7.54 mg/dL) in order to exclude potentially erroneous values. Calcium and phosphate values were then converted to millimolar (mM). Association was determined using linear regression using age, sex, and the top 10 genetic principal components as covariates.

For medications, we used concept IDs corresponding to calcitriol, colecalciferol, and teriparatide. Association was determined using linear regression using age, sex, and the top 10 genetic principal components as covariates.

For diagnosis codes, we considered the same phecodes that we used in the UK Biobank [252.2 (Hypoparathyroidism), 275.5 (Disorders of calcium or phosphorous metabolism), 350.1 (Tetany), 687.4 (Paresthesia), and 345.0/345.1/345.11/345.12 (Epilepsy)]. To capture phenotype data originally recorded using SNOMED terms in AOU, these phecodes were mapped to ICD-10 codes, which were mapped to SNOMED using the Owlready2 library's Pymedtermino2 module[15] , which utilizes UMLS data. Association was determined using Fisher's exact test.

The *All of Us* Research Program Resource Access Board (RAB) has granted an exception to the program's Data and Statistics Dissemination Policy for reporting exact participant counts of less than 20 in some of the analyses reporting in this study, due to the very low risk to participant privacy and very low risk of potential for re-identification.

Systematic Review of *CASR* variants previously associated with ADH1

In the UKB, we detected nine *CASR* variants previously associated with ADH1: c.310G>A [p.Val104Ile], c.372C>A [p.Asn124Lys], c.380A>G [p.Glu127Gly], c.452C>T [p.Thr151Met], c.613C>T [p.Arg205Cys], c.748G>A [p.Glu250Lys], c.1631G>A [p.Arg544Gln], c.2663C>T [p.Thr888Met], and c.2824G>A [p.Glu942Lys]. We subsequently reviewed the articles describing these variants and found peer-reviewed evidence supporting the pathogenicity of all[16–25] but c.748G>A [p.Glu250Lys], c.1631G>A [p.Arg544Gln], and c.2824G>A [p.Glu942Lys]. c.748G>A [p.Glu250Lys] was previously identified both in individuals with familial hypocalciuric hypercalcemia type 1 (FHH1, MIM: 145980) and ADH1 but was shown to have no impact on sensitivity to extracellular calcium *in vitro*[16]*.* c.1631G>A [p.Arg544Gln] was found in an individual with an ADH1 phenotype only in the recessive state[26] and was classified as Likely Benign with respect to ADH1 in ClinVar[27]. Similarly, c.2824G>A [p.Glu942Lys] was classified as Benign/Likely Benign in ClinVar. Thus, we excluded these three variants from the following analyses. Interestingly, c.613C>T [p.Arg205Cys] had been previously identified in both individuals with FHH1[28] and ADH1[24], although *in vitro* functional analysis was not performed. Of the remaining 6 variants, five of these variants are located in the ECD and structural analysis indicated that these variants are located in regions that are known to be important in receptor activation including the homodimer interface (p.Val104, p.Asn124, p.Glu127, p.Thr151) and ECD-cysteine-rich domain interface (p.Arg205) (**Figure S2** and **Table S5**).

In AOU, eight previously established ADH1 variants were detected (**Table 2**) across 16 individuals. These variants were in structurally important locations including the TM6-TM7 hotspot region (**Figure S1** and **Table S5**).

It is important to note that perhaps due to sample bias and the rarity of these variants, we were unable to detect associations in some variants that are well established to cause ADH1. For example, in the UKB, c.2663C>T [p.Thr888Met] showed no association with reduced serum calcium, even though the mutation disrupts negative regulation of CaSR[29] and was detected in hypocalcemic individuals[25]. Similarly, c.380A>G [p.Glu127Gly] did not show any relevant associations with ICD-10-based phecodes (and had no reported serum calcium or phosphate values), despite having been identified in a hypocalcemic individual[16] and occurring in the same location as other ADH1-associated mutations (c.380A>C [p.Glu127Ala][30] and c.379G>A [p.Glu127Lys][19,20]). In AOU, c.1810G>A [p.Glu604Lys] had no detected associations, but had also been found in multiple individuals with ADH1[16,31–33].

Variant Scoring in UKB and AOU

To prioritize pathogenic ADH1 variants, we calculated a variant score that was the sum of sub-scores indicating strength of association with ADH1 phenotypes and other characteristics consistent with a gain-of-function variant. We refer to this score as a variant's ADH1 score. We applied this score to all 479 rare (MAF<0.01) missense/nonsense/frameshift *CASR* variants as a recent paper that reviewed all ADH1 variants[34] reported 6 frameshifts and 1 nonsense variants. These variants are within the last exon of the *CASR* transcript and are likely to undergo NMD-escape to generate a protein. Several functional studies have shown that CaSR nonsense mutants produce CaSR protein, albeit at a smaller size corresponding to the amino acids lost by the truncation[35,36]. These nonsense variants usually lead to loss of part of the distal CaSR C tail that is known to bind regulators of cell surface expression such as dorfin, which mediates ubiquitin-mediated degradation[37], β-arrestin-1 that contributes to receptor internalization[38] and the putative dileucine endocytic motif that binds adaptor protein-2 and facilitates clathrin-mediated

endocytosis[39] Thus, these truncated variants have enhanced cell surface expression which increases receptor signaling and can result in ADH1. The first component of the score was association with serum calcium and phosphate levels, as measured in mmol/L. To calculate this sub-score, we applied linear regression models to each variant and serum calcium and phosphate levels and calculated the product of the β and -log10(p-values). The second component of the score was association with diagnosis code-based phenotypes (see sections for the UKB and AOU for phenotypes used). We used the sum of significance measurements (-log10(p-value)) of the resulting Fisher's exact tests as the sub-score. The third component of the score was whether individuals with the variant were taking medication related to ADH1. To calculate this sub-score, we again applied linear regression models to each variant and the number of relevant medications (for the UKB, we used all instances of p20003; for AOU, we used the concept IDs described in the AOU section). The number of appearances of 'calcichew', 'calichew d3 tablet', 'calcitriol', 'calcium citrate', 'calcium carbonate', 'colecalciferol', 'cholecalciferol', 'alfacalcidol', 'PTH', 'natpar', 'teriparatide', or 'forteo' in an individual's medical record was used as the response variable in the regression. The sub-score was the product of the β and -log10(p-values). The fourth component of the scores was whether a variant occurred at the same location as a previously described ADH1 variant, within a hotspot of ADH1 variants (amino acids 116–136[40] and 819–837[41]), or at the location of a variant discovered through a sponsored testing program. We assigned these variants a sub-score of 1. The fifth and final component of the score was the ACMG score from Varsome[42]. To calculate this sub-score, we used Varsome's API to extract the 'acmg_score', which is the result of a computational implementation of the ACMG criteria to classify variant pathogenicity[43]. We converted these scores to z-scores and lowered the weight of these scores by applying a ceiling of 0.25. This is because the ACMG criteria are not able to distinguish between GOF and LOF mutations.

As the observed empirical correlation matrix between the sub-scores showed that the sub-scores are almost uncorrelated (**Table S2**), we defined the overall score as the sum of the sub-scores. To set the weights of the sub-scores, we started with a value of 1 for all sub-scores. We then increased the value of the calcium sub-score, since hypocalcemia is observed in nearly all individuals with ADH1 described[44], and because some known variants had only associations with hypocalcemia in the UKB (such as c.310G>A [p.Val104Ile] and c.452C>T [p.Thr151Met]). We reduced the weight for predicted ACMG pathogenicity because it was unable to distinguish loss- from gain-of-function. We also reduced the weight for whether a variant occurred in a hotspot, because this was only indirect evidence of variant function. We arrived at the following weights: 3 for serum calcium, 1 for serum phosphate (since fewer, ~50%, individuals are hyperphosphatemic), 0.2 for Varsome ACMG, 0.5 for whether a variant occurs in the location of a known GOF variant or hotspot (amino acids 116–136[40] and 819–837[41] we assigned 0.5 based on the rationale that this criterion was suggestive but not sufficient to prioritize a variant), and 1 for each of the diagnosis code-based phenotypes. Variants were scored separately for each ethnicity (in the UKB) or ancestry (in AOU).

To arrive at a threshold for the ADH1 score, we created a null distribution of scores by resampling from each sub-score distribution of the synonymous variants to remove any correlation across the sub-scores (as would be expected for variants with no association with ADH1, **Figure S1**). We chose a threshold value of the score, 1.5, such that 98% of the scores from the null distribution fell below that threshold. This threshold yielded a specificity of 0.97 and sensitivity of 0.75 in the White British cohort and also similar sensitivity and specificity across all other ethnicities analyzed within the UKB based on previously known ADH1 pathogenic variants (**Table S3**). This score and threshold was then applied to all the ancestries across AOU which showed consistent sensitivity and specificity (**Table S4**).

Determination of sensitivity to extracellular calcium *in vitro*
To generate variant CaSR cell lines, WT and cmyc-tagged variant *CASR* were integrated into FlpIn TREx HEK293 cells (Invitrogen) to ensure a single copy was integrated per cell. All cells were maintained in Dulbecco's modified eagle medium (DMEM) (Thermo Fisher) supplemented with 5% fetal bovine serum (FBS) and antibiotic selection (200 µg/mL hygromycin, 5 µg/mL blasticidin). All five variants were expressed at the cell surface at levels comparable to WT CaSR (data not shown).

The response of each CaSR variant was then determined using a Ca_i^{2+} mobilization assay, which has been described in detail previously[45]. Briefly, FlpIn TRex HEK293 stable cell lines were seeded at 40,000

cells/well in poly-D-lysine (50 µg/mL) coated clear 96 well plates and incubated overnight at 37 °C in 5% CO_2 in the presence of 100 ng tetracycline. Cells were washed in assay buffer containing 150 mM NaCl, 2.6 mM KCl, 1.18 mM $MgCl_2$, 10 mM D-glucose, 10 mM HEPES, 0.1 mM $CaCl_2$, 0.5% BSA, 4 mM probenecid, pH 7.4, and loaded with 1 µM Fluo-8 AM (Abcam) in assay buffer for 1 hr. Calcium (Ca_o^{2+}) was added and measurements of Ca_i^{2+} mobilization were performed in duplicate at 37 °C using a FDSS/µCELL functional drug screening system (Hamamatsu) at 490 nm excitation and 520 nm emission. Data were normalized to the responses to assay buffer (0%) and 1 µM ionomycin (100%). The peak Ca_i^{2+} mobilization response was used for subsequent determination of the agonist response. The results are expressed at the mean ± SEM where n is the number of independent experiments. Nonlinear regression analysis was performed using GraphPad Prism® 10 (GraphPad Software, San Diego, CA).

Sponsored testing program

A genetic testing program sponsored by BridgeBio Pharma, Inc. was made available for individuals with suspected genetic hypoparathyroidism who met program eligibility criteria through Prevention Genetics. The next-generation sequencing panel has evolved to include 26 genes known to be associated with hypoparathyroidism: *ACADM* (MIM: 607008), *AIRE* (MIM: 607358), *ATP1A1* (MIM: 182310), *CASR*, *CHD7* (MIM: 608892), *CLDN16* (MIM: 603959), *CLDN19* (MIM: 610036), *CNNM2* (MIM: 607803), *DHCR7* (MIM: 602858), *EGF* (MIM: 131530), *FAM111A* (MIM: 615292), *FXYD2* (MIM: 601814), *GATA3* (MIM: 131320), *GCM2* (MIM: 603716), *GNA11* (MIM: 139313), *HADHA* (MIM: 600890), *HADHB* (MIM: 143450), *KCNA1* (MIM: 176260), *NEBL* (MIM: 605491), *PTH* (MIM: 168450), *SEMA3E* (MIM: 608166), *SLC12A3* (MIM: 600968), *SOX3* (MIM: 313430), *TBCE* (MIM: 604934), *TBX1* (MIM: 602054) and *TRPM6* (MIM: 607009).

A total of 169 samples between December 2020 and December 2022 were tested from participants with a mean ± SD age of 23.4 ± 20.4 (range 0–81) who were diagnosed with nonsurgical/idiopathic hypoparathyroidism (73.9%), hypocalcemia suspected to be of genetic cause (23.7%) or had a relative with a confirmed diagnosis of genetic hypoparathyroidism (2.4%). Pathogenic or likely pathogenic variants, and variants of uncertain significance were identified in 64 individuals (37.9%). Amongst these 64 participants, 77 variants were detected with 46.9% of variant harboring individuals documented as having unknown or no family history. In order of frequency, the relative number of individuals with detected variants are as follows: *CASR* 56.3% (36/64), *AIRE* 12.5% (8/64), *TBX1* 9.38% (6/64), *GATA3* 7.8% (5/64), *GNA11* 6.25% (4/64), *CHD7* 3.13% (2/64), *FAM111A* 3.13% (2/64), *PTH* 3.13% (2/64), *ACADM* 1.6% (1/64), *GCM2* 1.6% (1/64), *HADHB* 1.6% (1/64), and *TBCE* 1.6% (1/64). Of note, five individuals had variants identified in more than one gene.

Calculation of excess disease burden

A baseline frequency of individuals with any ADH1 phecode-based phenotype was calculated using phecodes 252.2 (Hypoparathyroidism), 275.5 (Disorders of calcium or phosphorous metabolism), 350.1 (Tetany), 687.4 (Paresthesia), 345.0/345.1/345.11/345.12 (Epilepsy), 594.0 (Urinary calculus), 594.1 (Calculus of kidney), and 585.3 (Chronic renal failure). This frequency was subtracted from the frequency of symptomatic individuals among individuals with ADH1 variants to calculate the excess frequency of symptomatic individuals among individuals with ADH1 variants. This excess frequency was multiplied by the number of individuals in each ethnicity (in the UKB) or ancestry (in AOU) to determine the excess disease burden for each variant.

Odds ratios of being symptomatic were also calculated, and p values were determined using a binomial test (**Table S13**).

Phenome-wide association study (pheWAS)

We tested for association sets of *CASR* variants with diagnosis code-based phenotypes. We calculated the burden score by assigning a value equal to the total number of alternate alleles an individual carried.

To test for associations, we performed SKAT-O test via the SKAT() command from the SKAT R package (v2.2.5)[46]. We also performed a logistic regression via the Python statsmodels package v0.14.1 to

determine the direction of effect for these variants. Age, sex, and the first 10 genetic principal components were used as covariates.

Three-dimensional modeling of CaSR structure

Snake plots were generated using GPCRdb.org[47]. CaSR three-dimensional modeling was undertaken using the reported three-dimensional cryo-EM and X-ray crystallography structures of either the CaSR ECD, CaSR ECD and TMD, and CaSR ECD and TMD with G proteins (Protein Data Bank (PDB) accession numbers: 8SZF, 8SZI, 5FBH, 7SIM, 7M3E, 7M3F, 7M3J[48–51]) in the PyMOL Molecular Graphics System (Version 2.5.2, Schrodinger, LLC).

Mass General Brigham Biobank data

The analysis in the Mass General Brigham (MGB) Biobank utilized whole-exome sequencing data for approximately 54,000 participants. Whole-exome sequencing data was conducted at the Broad Institute of Harvard and MIT. More details were described in previous publications[52]. Following removal of related individuals, we retained 39,081 individuals.

Calcium levels were averaged across repeated measurements for each participant. Close relative pairs were identified based on inferred kinship coefficients and excluded. Merging genetic and phenotype data resulted in n=35,509 participants for the calcium analysis. The analysis of calcium levels was performed using a linear regression model with sex and the first 10 genetic principal components as covariates.

Common variant fine-mapping and colocalization (SuSiE)

We used the Coloc-SuSiE[53] method to test for colocalization between serum calcium GWAS SNPs within the *CASR* region and GWAS loci for 55 other phenotypes. These phenotypes were identified through the GWAS Catalog[54], Open Targets[55], and literature searches for GWAS search terms and CASR. We identified 10 credible sets for serum calcium causal SNPs from a comprehensive biobank-based GWAS of serum calcium[56]. Each of these peaks was tested for colocalization with SNPs in the *CASR* region associated with other phenotypes, as well as GTEx[57] eQTLs for *CASR*. We applied an 80% probability threshold to determine colocalization between the serum calcium SNPs and those associated with other traits. Significant colocalization were observed for asthma, reticulocyte count, and mean corpuscular hemoglobin (probabilities of colocalization (H4) of 0.89, 0.90, and 0.99, respectively). No colocalization was observed between serum calcium variants and *CASR* expression in GTEx, likely due to limited sample sizes across tissues and significant eQTLs for *CASR* in only the pancreas (n = 328) and spleen (n = 241).

References

1. Sudlow, C., Gallacher, J., Allen, N., Beral, V., Burton, P., Danesh, J., Downey, P., Elliott, P., Green, J., Landray, M., et al. (2015). UK Biobank: An Open Access Resource for Identifying the Causes of a Wide Range of Complex Diseases of Middle and Old Age. PLoS Med. *12*, e1001779. https://doi.org/10.1371/journal.pmed.1001779.

2. Backman, J.D., Li, A.H., Marcketta, A., Sun, D., Mbatchou, J., Kessler, M.D., Benner, C., Liu, D., Locke, A.E., Balasubramanian, S., et al. (2021). Exome sequencing and analysis of 454,787 UK Biobank participants. Nature *599*, 628–634. https://doi.org/10.1038/s41586-021-04103-z.

3. Purcell, S., and Chang, C. PLINK v2.00a3.1LM. www.cog-genomics.org/plink/2.0/.

4. Chang, C.C., Chow, C.C., Tellier, L.C., Vattikuti, S., Purcell, S.M., and Lee, J.J. (2015). Second-generation PLINK: rising to the challenge of larger and richer datasets. Gigascience *4*, s13742-015-0047–0048. https://doi.org/10.1186/s13742-015-0047-8.

5. Hail-Team HAIL (v0.2.78).

6. Ganna, A., Genovese, G., Howrigan, D.P., Byrnes, A., Kurki, M.I., Zekavat, S.M., Whelan, C.W., Kals, M., Nivard, M.G., Bloemendal, A., et al. (2016). Ultra-rare disruptive and damaging mutations influence educational attainment in the general population. Nat. Neurosci. *19*, 1563–1565. https://doi.org/10.1038/nn.4404.

7. Sarnowski, C., Leong, A., Raffield, L.M., Wu, P., Vries, P.S. de, DiCorpo, D., Guo, X., Xu, H., Liu, Y., Zheng, X., et al. (2019). Impact of Rare and Common Genetic Variants on Diabetes Diagnosis by Hemoglobin A1c in Multi-Ancestry Cohorts: The Trans-Omics for Precision Medicine Program. Am. J. Hum. Genet. *105*, 706–718. https://doi.org/10.1016/j.ajhg.2019.08.010.

8. Kenny, C.M., Murphy, C.E., Boyce, D.S., Ashley, D.M., and Jahanmir, J. (2021). Things We Do for No Reason^{TM}: Calculating a “Corrected Calcium” Level. J. Hosp. Med. *16*, 499–501. https://doi.org/10.12788/jhm.3619.

9. Inker, L.A., Schmid, C.H., Tighiouart, H., Eckfeldt, J.H., Feldman, H.I., Greene, T., Kusek, J.W., Manzi, J., Lente, F.V., Zhang, Y.L., et al. (2012). Estimating Glomerular Filtration Rate from Serum Creatinine and Cystatin C. N. Engl. J. Med. *367*, 20–29. https://doi.org/10.1056/nejmoa1114248.

10. Bastarache, L. (2021). Using Phecodes for Research with the Electronic Health Record: From PheWAS to PheRS. Annu. Rev. Biomed. Data Sci. *4*, 1–19. https://doi.org/10.1146/annurev-biodatasci-122320-112352.

11. Moons, K.G.M., Groot, J.A.H. de, Bouwmeester, W., Vergouwe, Y., Mallett, S., Altman, D.G., Reitsma, J.B., and Collins, G.S. (2014). Critical Appraisal and Data Extraction for Systematic Reviews of Prediction Modelling Studies: The CHARMS Checklist. PLoS Med. *11*, e1001744. https://doi.org/10.1371/journal.pmed.1001744.

12. Moons, K.G.M., Altman, D.G., Reitsma, J.B., Ioannidis, J.P.A., Macaskill, P., Steyerberg, E.W., Vickers, A.J., Ransohoff, D.F., and Collins, G.S. (2015). Transparent Reporting of a multivariable prediction model for Individual Prognosis Or Diagnosis (TRIPOD): Explanation and Elaboration. Ann. Intern. Med. *162*, W1–W73. https://doi.org/10.7326/m14-0698.

13. Pavlou, M., Ambler, G., Seaman, S.R., Guttmann, O., Elliott, P., King, M., and Omar, R.Z. (2015). How to develop a more accurate risk prediction model when there are few events. BMJ : Br. Méd. J. *351*, h3868. https://doi.org/10.1136/bmj.h3868.

14. Investigators, A. of U.R.P., Denny, J.C., Rutter, J.L., Goldstein, D.B., Philippakis, A., Smoller, J.W., Jenkins, G., and Dishman, E. (2019). The "All of Us" Research Program. N. Engl. J. Med. *381*, 668–676. https://doi.org/10.1056/nejmsr1809937.

15. Lamy, J.-B. (2017). Owlready: Ontology-oriented programming in Python with automatic classification and high level constructs for biomedical ontologies. Artif. Intell. Med. *80*, 11–28. https://doi.org/10.1016/j.artmed.2017.07.002.

16. Hannan, F.M., Nesbit, M.A., Zhang, C., Cranston, T., Curley, A.J., Harding, B., Fratter, C., Rust, N., Christie, P.T., Turner, J.J.O., et al. (2012). Identification of 70 calcium-sensing receptor mutations in hyper- and hypo-calcaemic patients: evidence for clustering of extracellular domain mutations at calcium-binding sites. Hum. Mol. Genet. *21*, 2768–2778. https://doi.org/10.1093/hmg/dds105.

17. Hu, J., Mora, S., Colussi, G., Proverbio, M.C., Jones, K.A., Bolzoni, L., Ferrari, M.E.D., Civati, G., and Spiegel, A.M. (2009). Autosomal Dominant Hypocalcemia Caused by a Novel Mutation in the Loop 2 Region of the Human Calcium Receptor Extracellular Domain*. J. Bone Miner. Res. *17*, 1461–1469. https://doi.org/10.1359/jbmr.2002.17.8.1461.

18. Schouten, B.J., Raizis, A.M., Soule, S.G., Cole, D.R., Frengley, P.A., George, P.M., and Florkowski, C.M. (2010). Four cases of autosomal dominant hypocalcaemia with hypercalciuria including two with novel mutations in the calcium-sensing receptor gene. Ann. Clin. Biochem. *48*, 286–290. https://doi.org/10.1258/acb.2010.010139.

19. Lienhardt, A., Bai, M., Lagarde, J.-P., Rigaud, M., Zhang, Z., Jiang, Y., Kottler, M.-L., Brown, E.M., and Garabédian, M. (2001). Activating Mutations of the Calcium-Sensing Receptor: Management of Hypocalcemia. J. Clin. Endocrinol. Metab. *86*, 5313–5323. https://doi.org/10.1210/jcem.86.11.8016.

20. Hawkes, C.P., Shulman, D.I., and Levine, M.A. (2020). Recombinant human parathyroid hormone (1–84) is effective in CASR-associated hypoparathyroidism. Eur. J. Endocrinol. *183*, K13–K21. https://doi.org/10.1530/eje-20-0710.

21. Pearce, S.H.S., Williamson, C., Kifor, O., Bai, M., Coulthard, M.G., Davies, M., Lewis-Barned, N., McCredie, D., Powell, H., Kendall-Taylor, P., et al. (1996). A Familial Syndrome of Hypocalcemia with Hypercalciuria Due to Mutations in the Calcium-Sensing Receptor. N. Engl. J. Med. *335*, 1115–1122. https://doi.org/10.1056/nejm199610103351505.

22. Sørheim, J.I., Husebye, E.S., Nedrebø, B.G., Svarstad, E., Lind, J., Boman, H., and Løvås, K. (2010). Phenotypic Variation in a Large Family with Autosomal Dominant Hypocalcaemia. Horm. Res. Paediatr. *74*, 399–405. https://doi.org/10.1159/000303188.

23. Løvlie, R., Eiken, H.G., Sørheim, J.I., and Boman, H. (1996). The Ca2+-sensing receptor gene (PCAR1) mutation T151M in isolated autosomal dominant hypoparathyroidism. Hum. Genet. *98*, 129–133. https://doi.org/10.1007/s004390050174.

24. Ji, Y., Kang, C., Chen, J., and Zhang, L. (2021). Identification of p.Arg205Cys in CASR in an autosomal dominant hypocalcaemia type 1 pedigree. Medicine *100*, e26443. https://doi.org/10.1097/md.0000000000026443.

25. Lazarus, S., Pretorius, C.J., Khafagi, F., Campion, K.L., Brennan, S.C., Conigrave, A.D., Brown, E.M., and Ward, D.T. (2011). A novel mutation of the primary protein kinase C phosphorylation site in the calcium-sensing receptor causes autosomal dominant hypocalcemia. Eur. J. Endocrinol. *164*, 429–435. https://doi.org/10.1530/eje-10-0907.

26. Cavaco, B.M., Canaff, L., Nolin-Lapalme, A., Vieira, M., Silva, T.N., Saramago, A., Domingues, R., Rutter, M.M., Hudon, J., Gleason, J.L., et al. (2018). Homozygous Calcium-Sensing Receptor Polymorphism R544Q Presents as Hypocalcemic Hypoparathyroidism. J. Clin. Endocrinol. Metab. *103*, 2879–2888. https://doi.org/10.1210/jc.2017-02407.

27. Landrum, M.J., Lee, J.M., Riley, G.R., Jang, W., Rubinstein, W.S., Church, D.M., and Maglott, D.R. (2014). ClinVar: public archive of relationships among sequence variation and human phenotype. Nucleic Acids Res. *42*, D980–D985. https://doi.org/10.1093/nar/gkt1113.

28. Nissen, P.H., Christensen, S.E., Ladefoged, S.A., Brixen, K., Heickendorff, L., and Mosekilde, L. (2012). Identification of rare and frequent variants of the CASR gene by high-resolution melting. Clin. Chim. Acta *413*, 605–611. https://doi.org/10.1016/j.cca.2011.12.004.

29. Bai, M., Trivedi, S., Lane, C.R., Yang, Y., Quinn, S.J., and Brown, E.M. (1998). Protein Kinase C Phosphorylation of Threonine at Position 888 in Ca2+ o -Sensing Receptor (CaR) Inhibits Coupling to Ca2+ Store Release*. J. Biol. Chem. *273*, 21267–21275. https://doi.org/10.1074/jbc.273.33.21267.

30. Pollak, M.R., Brown, E.M., Estep, H.L., McLaine, P.N., Kifor, O., Park, J., Hebert, S.C., Seidman, C.E., and Seidman, J.G. (1994). Autosomal dominant hypocalcaemia caused by a Ca2+-sensing receptor gene mutation. Nat. Genet. *8*, 303–307. https://doi.org/10.1038/ng1194-303.

31. Alvarez-Hernandez, D., Santamaria, I., Rodriguez-Garcia, M., Iglesias, P., Delgado-Lillo, R., and Cannata-Andia, J. (2003). A novel mutation in the calcium-sensing receptor responsible for autosomal dominant hypocalcemia in a family with two uncommon parathyroid hormone polymorphisms. J. Mol. Endocrinol. *31*, 255–262. https://doi.org/10.1677/jme.0.0310255.

32. Iglesias, P., and Díez, J.J. (2006). Intracranial calcifications and activating mutation of the calcium-sensing receptor. J. Neurol., Neurosurg. Psychiatry *77*, 1243. https://doi.org/10.1136/jnnp.2006.097162.

33. García-Castaño, A., Madariaga, L., Nanclares, G.P. de, Ariceta, G., Gaztambide, S., and Castaño, L. (2019). Novel mutations associated with inherited human calcium-sensing receptor disorders: A clinical genetic study. Eur. J. Endocrinol. *180*, 59–70. https://doi.org/10.1530/eje-18-0129.

34. Roszko, K.L., Smith, L.M.S., Sridhar, A.V., Roberts, M.S., Hartley, I.R., Gafni, R.I., Collins, M.T., Fox, J.C., and Nemeth, E.F. (2022). Autosomal Dominant Hypocalcemia Type 1: A Systematic Review. J. Bone Miner. Res. *37*, 1926–1935. https://doi.org/10.1002/jbmr.4659.

35. Maruca, K., Brambilla, I., Mingione, A., Bassi, L., Capelli, S., Brasacchio, C., Soldati, L., Cisternino, M., and Mora, S. (2017). Autosomal dominant hypocalcemia due to a truncation in the C-tail of the calcium-sensing receptor. Mol. Cell. Endocrinol. *439*, 187–193. https://doi.org/10.1016/j.mce.2016.08.032.

36. Lienhardt, A., Garabédian, M., Bai, M., Sinding, C., Zhang, Z., Lagarde, J.-P., Boulesteix, J., Rigaud, M., Brown, E.M., and Kottler, M.-L. (2000). A Large Homozygous or Heterozygous In-Frame Deletion within the Calcium-Sensing Receptor's Carboxylterminal Cytoplasmic Tail That Causes Autosomal Dominant Hypocalcemia1. J. Clin. Endocrinol. Metab. *85*, 1695–1702. https://doi.org/10.1210/jcem.85.4.6570.

37. Huang, Y., Niwa, J., Sobue, G., and Breitwieser, G.E. (2006). Calcium-sensing Receptor Ubiquitination and Degradation Mediated by the E3 Ubiquitin Ligase Dorfin*. J. Biol. Chem. *281*, 11610–11617. https://doi.org/10.1074/jbc.m513552200.

38. Pi, M., Oakley, R.H., Gesty-Palmer, D., Cruickshank, R.D., Spurney, R.F., Luttrell, L.M., and Quarles, L.D. (2005). β-Arrestin- and G Protein Receptor Kinase-Mediated Calcium-Sensing Receptor Desensitization. Mol. Endocrinol. *19*, 1078–1087. https://doi.org/10.1210/me.2004-0450.

39. Nesbit, M.A., Hannan, F.M., Howles, S.A., Reed, A.A.C., Cranston, T., Thakker, C.E., Gregory, L., Rimmer, A.J., Rust, N., Graham, U., et al. (2013). Mutations in AP2S1 cause familial hypocalciuric hypercalcemia type 3. Nat. Genet. *45*, 93–97. https://doi.org/10.1038/ng.2492.

40. Wu, Y., Zhang, C., Huang, X., Cao, L., Liu, S., and Zhong, P. (2022). Autosomal dominant hypocalcemia with a novel CASR mutation: a case study and literature review. J. Int. Méd. Res. *50*, 03000605221110489. https://doi.org/10.1177/03000605221110489.

41. Hu, J., McLarnon, S.J., Mora, S., Jiang, J., Thomas, C., Jacobson, K.A., and Spiegel, A.M. (2005). A Region in the Seven-transmembrane Domain of the Human Ca2+ Receptor Critical for Response to Ca2+ *. J. Biol. Chem. *280*, 5113–5120. https://doi.org/10.1074/jbc.m413403200.

42. Kopanos, C., Tsiolkas, V., Kouris, A., Chapple, C.E., Aguilera, M.A., Meyer, R., and Massouras, A. (2018). VarSome: the human genomic variant search engine. Bioinformatics *35*, 1978–1980. https://doi.org/10.1093/bioinformatics/bty897.

43. Kopanos, C., Tsiolkas, V., Kouris, A., Chapple, C.E., Aguilera, M.A., Meyer, R., and Massouras, A. (2018). VarSome: the human genomic variant search engine. Bioinformatics *35*, 1978–1980. https://doi.org/10.1093/bioinformatics/bty897.

44. Roszko, K.L., Smith, L.M.S., Sridhar, A.V., Roberts, M.S., Hartley, I.R., Gafni, R.I., Collins, M.T., Fox, J.C., and Nemeth, E.F. (2022). Autosomal Dominant Hypocalcemia Type 1: A Systematic Review. J. Bone Miner. Res. *37*, 1926–1935. https://doi.org/10.1002/jbmr.4659.

45. Josephs, T.M., Keller, A.N., Khajehali, E., DeBono, A., Langmead, C.J., Conigrave, A.D., Capuano, B., Kufareva, I., Gregory, K.J., and Leach, K. (2020). Negative allosteric modulators of the human calcium-sensing receptor bind to overlapping and distinct sites within the 7-transmembrane domain. Br. J. Pharmacol. *177*, 1917–1930. https://doi.org/10.1111/bph.14961.

46. Lee, S., Emond, M.J., Bamshad, M.J., Barnes, K.C., Rieder, M.J., Nickerson, D.A., Team, N.G.E.S.P.L.P., Christiani, D.C., Wurfel, M.M., and Lin, X. (2012). Optimal Unified Approach for Rare-Variant Association Testing with Application to Small-Sample Case-Control Whole-Exome Sequencing Studies. Am. J. Hum. Genet. *91*, 224–237. https://doi.org/10.1016/j.ajhg.2012.06.007.

47. Pándy-Szekeres, G., Munk, C., Tsonkov, T.M., Mordalski, S., Harpsøe, K., Hauser, A.S., Bojarski, A.J., and Gloriam, D.E. (2017). GPCRdb in 2018: adding GPCR structure models and ligands. Nucleic Acids Res. *46*, gkx1109-. https://doi.org/10.1093/nar/gkx1109.

48. He, F., Wu, C.-G., Gao, Y., Rahman, S.N., Zaoralová, M., Papasergi-Scott, M.M., Gu, T.-J., Robertson, M.J., Seven, A.B., Li, L., et al. (2024). Allosteric modulation and G-protein selectivity of the Ca2+-sensing receptor. Nature *626*, 1141–1148. https://doi.org/10.1038/s41586-024-07055-2.

49. Gao, Y., Robertson, M.J., Rahman, S.N., Seven, A.B., Zhang, C., Meyerowitz, J.G., Panova, O., Hannan, F.M., Thakker, R.V., Bräuner-Osborne, H., et al. (2021). Asymmetric activation of the calcium-sensing receptor homodimer. Nature *595*, 455–459. https://doi.org/10.1038/s41586-021-03691-0.

50. Park, J., Zuo, H., Frangaj, A., Fu, Z., Yen, L.Y., Zhang, Z., Mosyak, L., Slavkovich, V.N., Liu, J., Ray, K.M., et al. (2021). Symmetric activation and modulation of the human calcium-sensing receptor. Proc. Natl. Acad. Sci. *118*, e2115849118. https://doi.org/10.1073/pnas.2115849118.

51. Zhang, C., Zhang, T., Zou, J., Miller, C.L., Gorkhali, R., Yang, J.-Y., Schilmiller, A., Wang, S., Huang, K., Brown, E.M., et al. (2016). Structural basis for regulation of human calcium-sensing receptor by magnesium ions and an unexpected tryptophan derivative co-agonist. Sci. Adv. *2*, e1600241. https://doi.org/10.1126/sciadv.1600241.

52. Boutin, N.T., Schecter, S.B., Perez, E.F., Tchamitchian, N.S., Cerretani, X.R., Gainer, V.S., Lebo, M.S., Mahanta, L.M., Karlson, E.W., and Smoller, J.W. (2022). The Evolution of a Large Biobank at Mass General Brigham. J. Pers. Med. *12*, 1323. https://doi.org/10.3390/jpm12081323.

53. Wallace, C. (2021). A more accurate method for colocalisation analysis allowing for multiple causal variants. PLoS Genet. *17*, e1009440. https://doi.org/10.1371/journal.pgen.1009440.

54. Cerezo, M., Sollis, E., Ji, Y., Lewis, E., Abid, A., Bircan, K.O., Hall, P., Hayhurst, J., John, S., Mosaku, A., et al. (2024). The NHGRI-EBI GWAS Catalog: standards for reusability, sustainability and diversity. Nucleic Acids Res. *53*, D998–D1005. https://doi.org/10.1093/nar/gkae1070.

55. Buniello, A., Suveges, D., Cruz-Castillo, C., Llinares, M.B., Cornu, H., Lopez, I., Tsukanov, K., Roldán-Romero, J.M., Mehta, C., Fumis, L., et al. (2024). Open Targets Platform: facilitating therapeutic hypotheses building in drug discovery. Nucleic Acids Res. *53*, D1467–D1475. https://doi.org/10.1093/nar/gkae1128.

56. Sakaue, S., Kanai, M., Tanigawa, Y., Karjalainen, J., Kurki, M., Koshiba, S., Narita, A., Konuma, T., Yamamoto, K., Akiyama, M., et al. (2021). A cross-population atlas of genetic associations for 220 human phenotypes. Nat. Genet. *53*, 1415–1424. https://doi.org/10.1038/s41588-021-00931-x.

57. Lonsdale, J., Thomas, J., Salvatore, M., Phillips, R., Lo, E., Shad, S., Hasz, R., Walters, G., Garcia, F., Young, N., et al. (2013). The Genotype-Tissue Expression (GTEx) project. Nat. Genet. *45*, 580–585. https://doi.org/10.1038/ng.2653.
