# Supplementary material for: A calcium-sensing receptor allelic series and underdiagnosis of genetically driven hypocalcemia
Source: Am J Hum Genet. 2025 Jul 14;112(8):1818–32. doi: 10.1016/j.ajhg.2025.06.013 (PMC12414667; doi:10.1016/j.ajhg.2025.06.013)
Supplement: Document S2. Article plus supplemental information [file mmc3.pdf]

A calcium-sensing receptor allelic series and underdiagnosis of genetically driven hypocalcemia

Graphical abstract

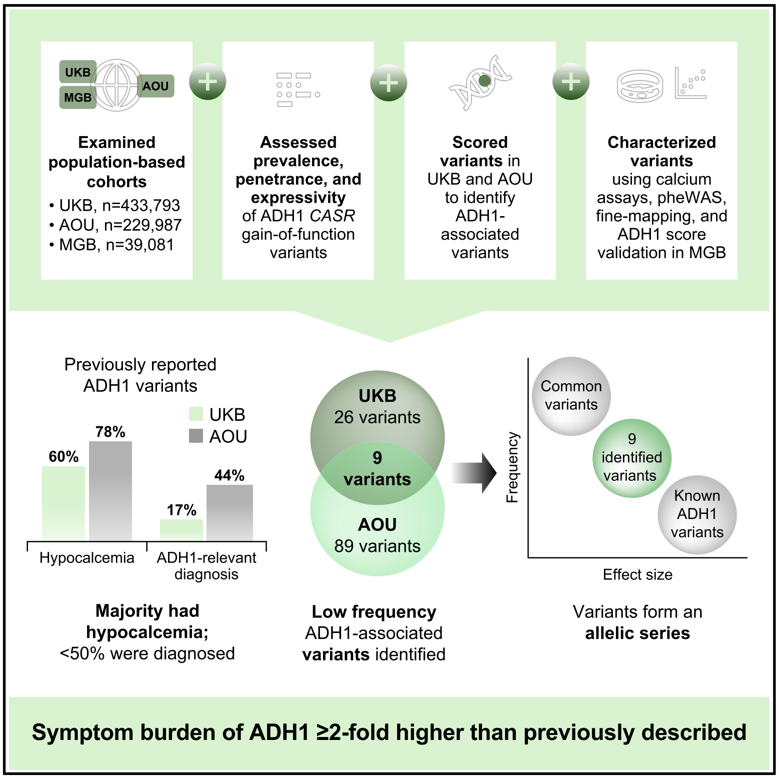

Authors

Jeremy B. Chang, Connor P. Barnhill, Alexander M. Apostolov, ..., Jonathan C. Fox, Christoph Lange, Sun-Gou Ji

Correspondence

jeremy.chang@bridgebio.com (J.B.C.),
sun-gou.ji@bridgebio.com (S.-G.J.)

We examined variants of *CASR* associated with autosomal-dominant hypocalcemia type 1 (ADH1) and created a score to identify 9 additional variants. The symptom burden of ADH1 was ≥2-fold higher than previously appreciated. Our approach is applicable to other genetic diseases where symptom burden may also be higher than appreciated.

Chang et al., 2025, The American Journal of Human Genetics *112*, 1818–1832
August 7, 2025 © 2025 The Author(s). Published by Elsevier Inc. on behalf of American Society of Human Genetics.
https://doi.org/10.1016/j.ajhg.2025.06.013

ARTICLE

A calcium-sensing receptor allelic series and underdiagnosis of genetically driven hypocalcemia

Jeremy B. Chang,[1,*] Connor P. Barnhill,[1] Alexander M. Apostolov,[2] Marcus M. Soliai,[1] Julian Hecker,[3,4] Jovia L. Nierenberg,[1] Lyndsay M. Stapleton Smith,[1] Arun S. Mathew,[1] Xue Zeng,[1] Jiayin Diao,[5] C. Dilanka Fernando,[5] Qingwen Chen,[3,4] Ben W. Dulken,[1,6] Aleksandr Petukhov,[1] Russ Altman,[7] Tracy M. Josephs,[5] Jessica A. Lasky-Su,[3,4] Caroline M. Gorvin,[8,9] Mary Scott Roberts,[1] Scott H. Adler,[1] Jonathan C. Fox,[1] Christoph Lange,[3,10,11] and Sun-Gou Ji[1,*]

Summary

The availability of genomic sequencing has revealed that variants in genes that cause rare monogenic disorders are relatively common, which raises the question of variant pathogenicity. Autosomal-dominant hypocalcemia type 1 (ADH1) is a rare genetic form of hypoparathyroidism caused by gain-of-function (GoF) variants in the calcium-sensing receptor (CaSR) encoded by *CASR*. We examined the prevalence, penetrance, and expressivity of GoF *CASR* variants in the UK Biobank (UKB; $n = 433{,}793$), All of Us (AOU; $n = 229{,}987$), and Mass General Brigham Biobank ($n = 39{,}081$). Individuals with previously reported ADH1-associated variants indeed showed ADH1 symptoms, including hypocalcemia (60% in the UKB and 78% in AOU). However, less than half had an ADH1-relevant diagnosis code (17% in the UKB and 44% in AOU), suggesting that individuals with ADH1 are present in these biobanks but may be underdiagnosed. We then developed a scoring algorithm and identified nine low-frequency ADH1-associated variants, which were further validated using genetic sequencing of individuals with nonsurgical hypoparathyroidism ($n = 169$) and an *in vitro* functional assay. These nine variants have an intermediate effect and frequency relative to previously reported ADH1-associated variants, completing an allelic series with respect to serum calcium, and alone are responsible for a symptom burden roughly equivalent to all previously reported ADH1-associated variants. Our work indicates that hypocalcemia due to GoF in *CASR* with ADH1-associated symptoms is underdiagnosed, provides a deeper understanding of the genotype-phenotype relationship of *CASR* variants, and illustrates that variants in genes underlying rare disorders may cause a much greater symptom burden than currently appreciated.

Introduction

An estimated 1 in 10 people suffer from a rare disease, and 72% of these diseases are thought to have a genetic basis.[1,2] Nevertheless, precise estimation of the symptom burden (i.e., number and severity of symptomatic individuals) of rare genetic disease is challenging given potential ascertainment bias, lack of specific diagnostic criteria, small numbers of affected individuals, and clinical heterogeneity. In addition, incomplete penetrance and variable expressivity of seemingly pathogenic variants complicate the matter further. The development of a framework for examining rare monogenic diseases within large population-based cohorts can enhance patient care by refining prevalence estimates, delineating symptom burden, elucidating variant penetrance and expressivity, and identifying additional variants that contribute to the phenotypic spectrum. A better understanding of disease-causing variants and their impact at a population level can bring resources and awareness to rare disease communities.

Autosomal-dominant hypocalcemia type 1 (ADH1; MIM: 601198) is a rare genetic form of hypoparathyroidism caused by gain-of-function (GoF) variants in the calcium-sensing receptor (CaSR) encoded by *CASR* (MIM: 601199). At the molecular level, the CaSR is a G-protein-coupled receptor (GPCR) that regulates calcium homeostasis, in part by modulating secretion of the parathyroid hormone.[3] Historically, individuals with ADH1 have primarily been characterized in the context of small familial cohorts,[4] which can lead to an incomplete understanding of the genotype-phenotype relationship.

So far, at least 121 *CASR* variants have been described in association with ADH1.[4–8] Loss-of-function (LoF) variants are also observed in association with familial hypocalciuric hypercalcemia type 1 (FHH1; MIM: 145980).[9] These LoF variants are more frequent in the general population than GoF variants and can complicate the identification of

[1]BridgeBio Pharma, 3160 Porter Drive, Suite 250, Palo Alto, CA 94304, USA; [2]Department of Bioengineering, Stanford University, Stanford, CA 94305, USA; [3]Channing Division of Network Medicine, Brigham and Women's Hospital, Boston, MA 02115, USA; [4]Harvard Medical School, Boston, MA 02115, USA; [5]Drug Discovery Biology Theme, Monash Institute of Pharmaceutical Sciences, Monash University, Parkville, VIC 3052, Australia; [6]Department of Pathology, Stanford School of Medicine, Stanford, CA 94305, USA; [7]Departments of Bioengineering, Genetics, and Medicine, Stanford University, Stanford, CA 94305, USA; [8]Institute of Metabolism and Systems Research (IMSR) and Centre for Diabetes, Endocrinology and Metabolism (CEDAM), University of Birmingham, Birmingham B15 2TT, UK; [9]Centre for Membrane Proteins and Receptors (COMPARE), Universities of Birmingham and Nottingham, Birmingham B15 2TT, UK; [10]Channing Division of Network Medicine, Brigham and Women's Hospital, Boston, MA 02115, USA; [11]Department of Biostatistics, Harvard T.H. Chan School of Public Health, Boston, MA 02115, USA
*Correspondence: jeremy.chang@bridgebio.com (J.B.C.), sun-gou.ji@bridgebio.com (S.-G.J.)
https://doi.org/10.1016/j.ajhg.2025.06.013.
© 2025 The Author(s). Published by Elsevier Inc. on behalf of American Society of Human Genetics.
This is an open access article under the CC BY license (http://creativecommons.org/licenses/by/4.0/).

GoF variants, especially when functional characterization is not performed because both are considered pathogenic.

Phenotypically, ADH1 has variable expressivity where individuals with the same genetic variant can show a wide range of clinical symptoms.[10] It is primarily characterized by persistent hypocalcemia in the setting of low parathyroid hormone levels. This hypocalcemia can cause paresthesia, tetany, and, in severe cases, cardiac arrhythmias, laryngospasm, and seizures. Individuals can experience hypercalciuria, which can be exacerbated by calcium supplementation, leading to kidney stones and chronic kidney disease. ADH1 has also been described with features of Bartter's syndrome, including hypomagnesemia, hypokalemia, and metabolic alkalosis.[11] Therefore, diagnosing ADH1 is not straightforward, and no specific diagnosis code existed until October 2023.

To clarify the genotype-phenotype relationship of *CASR* variants, we characterized variants and associated phenotypes in three population-based biobanks: the UK Biobank (UKB), All of Us (AOU), and the Mass General Brigham (MGB) Biobank. Using the example of ADH1, we provide a framework to identify and characterize pathogenic variants of rare diseases in large biobanks and illustrate how such an approach can complement studies from familial and clinically ascertained cohorts.

Subjects, material, and methods

UKB

The UKB is a large-scale biomedical database containing genetic, lifestyle, health, and clinical information from 500,000 participants.[12,13] Self-reported ethnicities from the UKB that were included in our analysis are "Black African," "Caribbean," "Chinese," "White British," "Indian," and "Pakistani." Quality control on all qualifying variants within the Matched Annotation from NCBI and ENBL-EBI (MANE) transcript of *CASR* (ENST00000639785.2, GenBank: NM_000388.4)[14] was performed using PLINK (v.2.00a3.1LM)[15] and HAIL (v.0.2.78).[16] Serum calcium levels were uncorrected for albumin. The estimated glomerular filtration rate (eGFR) was calculated using the 2012 CKD-EPI cystatin C equation.[17] We extracted ICD-10 codes and medications from both in-patient and general practitioner (GP) data. ICD-10 codes were then mapped to a total of 1,518 phenotypes (phecodes) using the Phecode Map 1.2.[18] Phecodes we considered to be ADH1 associated were 252.2 (hypoparathyroidism), 275.5 (disorders of calcium or phosphorous metabolism), 350.1 (tetany), 687.4 (paresthesia), and 345.0/345.1/345.11/345.12 (various forms of epilepsy). Locus-phenotype associations were tested using Fisher's exact test, and for locus-phenotype combinations with at least 10 events per variable, we used covariate/PC-adjusted logistic regression with Firth correction. Based on the similarity of the results from logistic regression with Firth correction and Fisher's exact test for combinations with more than 10 events per variable (Table S1) and the substantial number of combinations with fewer events, we used *p* values from Fisher's exact test for subsequent analyses. For medications, the count of instances of "calcichew," "calcichew d3 tablet," "colecalciferol," "cholecalciferol," "calcitriol," "calcium citrate," "calcium carbonate," "alfacalcidol," "PTH," "natpar," "teriparatide," and "forteo" was determined for each individual, and they were considered ADH1-associated medications. All regression analyses were conducted separately in each self-reported ethnicity group, and the results were meta-analyzed. Further details on the study, exome sequencing, and phenotypes are in the supplemental methods.

AOU

The AOU Research Program is a longitudinal cohort study led by the National Institutes of Health for the advancement of precision medicine.[19] Genetically determined ancestry groups in AOU are genetic ancestries similar to the African ancestry (AFR), American ancestry (AMR), East Asian ancestry (EAS), European ancestry (EUR), Middle Eastern ancestry (MID), and South Asian ancestry (SAS) 1000 Genomes population references and will be referred to as AFR-like, AMR-like, EAS-like, EUR-like, MID-like, and SAS-like, respectively. For serum calcium levels, we used the concept "calcium [mass/volume] in serum or plasma," and for serum phosphate levels, we used "phosphate [mass/volume] in serum or plasma." For diagnosis codes, we considered the same phecodes that we used in the UKB. To capture phenotype data originally recorded using SNOMED terms in AOU, these phecodes were mapped to ICD-10 codes, which were mapped to SNOMED using the Owlready2 library's Pymedtermino2 module,[20] which utilizes Unified Medical Language System (UMLS) data. For ADH1-associated medications, we used concept IDs corresponding to calcitriol, colecalciferol, and teriparatide. Additional information on the study, whole-genome sequencing, and phenotypes are in the supplemental methods.

Variant scoring in UKB and AOU

To prioritize pathogenic ADH1 variants,[21–30] we calculated a variant score as the sum of sub-scores indicating the strength of association with ADH1 phenotypes and other characteristics consistent with a GoF variant. We refer to this score as a variant's ADH1 score, which was composed of the variant's association with serum calcium and phosphate levels, the presence of the variant in any individual with an ADH1-associated diagnosis or medication, whether the location of the variant was in a hotspot for a known GoF variant (amino acids 116–136[5] and 819–837[31]), and an ensemble of *in silico* pathogenicity predictors through Varsome.[32] The significance of the sub-scores was tested using Fisher's exact test for binary phenotypes/logistic regression with Firth correction or linear regression for quantitative phenotypes. The weights of the sub-scores were defined as 3 for serum

calcium, 1 for serum phosphate (since fewer, ~50%, individuals are hyperphosphatemic), 1 for each of the ADH1-associated diagnoses or medications, 0.5 for whether it occurred at the same location as a known GoF variant, and 0.2 for Varsome predictions. All variants remaining after hard filtering were scored. Details are described further in the supplemental methods, and the correlation matrix between the sub-scores for each self-reported ethnicity is shown in Table S2. The threshold for a significance of 1.5 was defined such that 98% of the scores from the null distribution (created by resampling sub-scores of synonymous variants) fell below that threshold (Figure S1). This threshold yielded the expected specificity of 0.98 with respect to synonymous variants and a sensitivity of 0.75 in a subset of participants with self-reported White British ethnicity determined to be genetically homogeneous by principal-component analysis (Field 22006) and had similar sensitivity and specificity in all other self-reported ethnicities (Field 21000) in the UKB based on known ADH1 pathogenic variants (Table S3). Self-reported ethnicity is used as a proxy for genetic ancestry because a true genetically homogeneous population is not provided in the UKB for ancestries other than White British. This score and threshold were then applied to all genetic ancestries in AOU, which showed consistent sensitivity and specificity (Table S4). Full summary statistics of variants considered in this study are available in the supplemental information. The code for calculating the ADH1 variant score is available on Zenodo (https://doi.org/10.5281/zenodo.15428300).

In vitro and computational analyses

To generate variant CASR cell lines, wild-type (WT) and cmyc-tagged variant *CASR*s were integrated into FlpIn TREx HEK293 cells (Invitrogen) using isogenic integration at the Flp-recombinase integration site, and cells underwent hygromycin antibiotic selection to ensure a single copy was integrated per cell. cmyc-$CaSR_{variant}$ expression was under the control of tetracycline, thus allowing titration of cmyc-$CaSR_{variant}$. The response of each CaSR variant was then determined using a Ca_i^{2+} mobilization assay using fluorescence-activated cell sorting (FACS) analysis on a FACS Canto II (Becton Dickinson) as described previously.[33]

The excess burden of disease was calculated using phecode-based phenotypes. A pheWAS of *CASR* variants was conducted using the SKAT-O test via the SKAT() function from the SKAT R package (v.2.2.5).[34] We also performed a logistic regression using the Python statsmodels package v.0.14.1 to determine the direction of effect for these variants. Age, sex, and the first 10 genetic principal components were used as covariates. We fine mapped the *CASR* region and tested for colocalization with 55 other phenotypes using Coloc-SuSiE.[35] Experimental details and computational methods are described further in the supplemental methods.

MGB Biobank

The analysis in the MGB[36] utilized exome sequencing data from approximately 54,000 participants. Following the removal of related individuals, we retained 39,081 individuals. Calcium levels were averaged across repeated measurements for each participant. Close relative pairs were identified based on inferred kinship coefficients and excluded, with a KING kinship coefficient cutoff of 0.0884. Merging genetic and phenotype data resulted in $n = 35{,}509$ participants for the calcium analysis. The analysis of calcium levels was performed using a linear regression model with sex and the first 10 genetic principal components as covariates.

Ethics

Each study was approved by study-specific institutional review boards, and informed consent was obtained from all study participants. The publication of summaries involving fewer than 20 participants was approved by special exemption from the AOU Research Program Resource Access Board due to the rarity of ADH1. To protect participant privacy, demographic details that could potentially lead to re-identification have been excluded.

Results

Confirmation that *CASR* variants previously associated with ADH1 were associated with ADH1 phenotypes in the UKB and AOU

Five previously reported ADH1-associated variants[4–8] (c.310G>A [p.Val104Ile], c.372C>A [p.Asn124Lys], c.452C>T p.Thr151Met], c.613C>T [p.Arg205Cys], and c.2663C>T [p.Thr888Met]) were found in a heterozygous state in 10 individuals in the UKB (7 females and 3 males; Table 1; a systematic review of the location and structural modeling of the variants is provided in Table S5, and a structural analysis of the variants is shown in Figure S2). The majority of individuals in the UKB had only a single measurement of serum calcium (85%; Figure S3A), and no significant differences in mean serum calcium level were detected across self-reported ethnicities (ANOVA; Figure S3B). Of the 10 heterozygotes with reported values (Figure S3C), six (or 60%) had serum calcium below the lower limit of normal (LLN; 2.2 mM[37]), whereas only 2% of UKB participants, overall, had serum calcium below the LLN (p = 1.0E−11, t test; Table 1; Figure S3D). Four of 10 heterozygotes were above the upper limit of normal (ULN) for serum phosphate (1.45 mM[38]; Table 1), which is similar to previous reports.[4] No significant differences in mean serum phosphate levels were detected across self-reported ethnicities except between the UKB self-reported Black African and Indian, White British and Indian, and Caribbean and Indian cohorts (p = 1E10−4, 2.6E−2, and 8.7E−3, respectively; Tukey honestly significant difference [HSD]; Figure S3E). At least one ADH1-related medication or symptom was observed (nominal

Table 1. Characteristics of variants previously associated with ADH1 detected in the UKB

Variant	Self-reported ethnicity	No. het	No. hom	Frequency in biobank	MAF in ethnicity	Calcium			Phosphate			$eGFR_{cy}$		Age	Sex	Ca^{2+} (mM)	Phosphate (mM)	Relevant meds	$eGFR_{cy}$ (mL/min/1.73 m^2)	Diagnoses
						Mean (mM)	Beta (mM)	*p* value	Mean (mM)	Beta (mM)	*p* value	Beta (mL/min/1.73 m^2)	*p* value							
c.310G>A (p.Val104Ile)[a]	White British	1	0	7.4E−06	2.8E−06	2.13	−0.24	9.1E−03	1.46	0.35	2.2E−02	−4.1	6.9E−01	59	M	2.13[b]	1.46[c]	N/A	76.5	N/A
	Indian	3	0	7.4E−06	7.8E−04	2.14	−0.24	1.8E−06	1.56	0.41	1.2E−06	−0.6	9.5E−01	55	M	2.07[b]	1.44	N/A	79.5	N/A
			0											55	F	2.07[b]	1.70[c]	N/A	78.5	N/A
			0											58	M	2.19[b]	1.51[c]	N/A	81.4	tetany (OR 157.6, $p = 0.01$)[d]
c.372C>A (p.Asn124Lys)[a]	White British	2	0	2.1E−06	5.5E−06	2.20	−0.19	3.1E−03	1.25	0.05	6.4E−01	−20.4	4.4E−02	70	F	2.19[b]	1.27	calcitriol	71.8	hypoparathyroidism (OR 836, $p =$ 2.4E−03)[d]
			0											53	F	2.20[b]	1.23	N/A	66.0	N/A
c.452C>T (p.Thr151Met)[a]	White British	1	0	1.1E−06	2.8E−06	1.96	−0.43	4.5E−06	1.57	0.38	1.5E−02	10.0	4.9E−01	52	F	1.96[b]	1.57[c]	N/A	109.4	N/A
c.613C>T (p.Arg205Cys)	Chinese	1	0	2.1E−06	7.2E−04	2.39	−0.01	9.0E−01	1.04	−0.21	1.8E−01	10.8	3.4E−01	66	F	2.39	1.04	N/A	103.6	N/A
c.2663C>T (p.Thr888Met)	White British	2	0	2.1E−06	5.5E−06	2.29	−0.10	1.3E−01	1.33	0.14	2.1E−01	5.4	6.0E−01	60	F	2.22	1.37	N/A	98.8	N/A
			0											59	F	2.36	1.29	N/A	95.1	N/A

$eGFR_{cy}$, estimated glomerular filtration rate calculated using the CKD-EPI cystatin C equation (2012).[17] Bonferroni significance thresholds were 7.1E−3 for calcium, phosphate, and $eGFR_{cy}$ and 6.2E−3 for medications and diagnoses. Lower limit of normal (LLN) calcium is 2.2 mM, and upper limit of normal (ULN) phosphate is 1.45 mM. OR and *p* values in the "relevant meds" and "diagnoses" columns are for the medication or diagnosis for the variant in each self-reported ethnicity. F, female; het, heterozygous; hom, homozygous; Inf, infinite; M, male; MAF, minor-allele frequency; meds, medications; OR, odds ratio.
[a]At least one nominally significant association.
[b]Below LLN.
[c]Above ULN.
[d]$p < 0.05$ (nominal significance threshold).

significance threshold $p < 0.05$) in individuals with c.310G>A (p.Val104Ile), c.372C>A (p.Asn124Lys), and c.452C>T (p.Thr151Met). Three individuals outside of the analysis cohort due to self-reported ethnicity or relatedness exclusions had a previously reported ADH1 variant: c.380A>G (p.Glu127Gly), c.310G>A (p.Val104Ile), or c.613C>T (p.Arg205Cys) (Table S6).

In AOU, eight previously established ADH1 variants were detected across 16 individuals[21,23,39–42] (Table 2; a systematic review of the location and structural modeling of the variants is provided in Table S5, and structural analysis of the variants is shown in Figure S2). We detected at least one nominal ADH1-related association (nominal significance threshold $p \leq 0.05$) in six of eight variants. All six had at least a nominally significant association with reduced serum calcium levels in at least one genetically determined ancestry, including two of the three variants also found in the UKB (c.310G>A [p.Val104Ile] and c.452C>T [p.Thr151Met]). Three of these variants were also associated with calcium-increasing medications (c.310G>A [p.Val104Ile], c.2431A>G [p.Met811Val], and c.2503G>A [p.Ala835Thr]). At least nominally significant associations between three out of eight variants with ADH1-related diagnoses were detected (Table 2).

We detected no associations with ADH1 phenotypes in individuals with Arg205 in either the UKB or AOU. Interestingly, c.613C>T (p.Arg205Cys) is reported in ClinVar,[43] as observed in both individuals with FHH1[44] and ADH1[29] with no functional analyses listed. Therefore, this variant was excluded from the known ADH1 variants list used for the following prevalence and penetrance estimates, as well as for developing the ADH1 score.

Frequency and symptom burden due to *CASR* variants previously associated with ADH1 in the UKB, AOU, TOPMed, and gnomAD

In the UKB, the frequency of previously established ADH1-associated variants was 2.4:100,000. In AOU, the frequency was 3.9:100,000. Similarly, five previously established variants were observed (c.372C>A [p.Asn124Lys], c.452C>T [p.Thr151Met], c.1767C>G [p.Phe589Leu], c.2530G>A [p.Ala844Thr], and c.2647G>A [p.Val883Met]) in TOPMed[45] with a frequency of 3.8:100,000, and five (c.310G>A [p.Val104Ile], c.1767C>G [p.Phe589Leu], c.2330T>C [p.Ile777Thr], c.2530G>A [p.Ala844Thr], and c.2647G>A [p.Val883Met]) were observed in gnomAD[46] with a frequency of 4.6:100,000. These frequencies were similar to a previously reported[9] frequency of 3.9:100,000 within the Geisinger DiscovEHR cohort.

Although these variants are pathogenic for ADH1, their symptom burden can be variable. Clinically, ADH1 is heterogeneous, with only 73% of diagnosed individuals experiencing hypocalcemia-related symptoms.[4] In the UKB, among individuals with both in-patient and primary care records, we found that 1/6 (17%) heterozygotes had an ICD-10 code directly suggestive of diagnosis of ADH1 (hypocalcemia, disorder of calcium/phosphorous metabolism, or hypoparathyroidism), despite the high frequency of hypocalcemia observed based on calcium measurements, with 6/10 (60%) heterozygotes at or below the serum calcium LLN. Within AOU, 4/9 (44%) had a diagnosis directly suggestive of ADH1. The lower rate of diagnoses in both cohorts could suggest underdiagnosis or incomplete coverage of medical history.

Although the UKB and AOU may trend healthier than the general population, we observed that individuals with ADH1 are well captured in both cohorts. This gave us confidence that these two cohorts could be mined to identify additional ADH1 variants.

Development of a variant score to identify hypocalcemia-associated variants in the UKB and AOU

Because ADH1 was only recently assigned a specific ICD-10 code (E20.810) and due to the incomplete penetrance and variable expressivity we observed across known ADH1-associated variants, it was unlikely that a single phenotype could identify individuals with ADH1. Therefore, we undertook a holistic evaluation of known ADH1 phenotypes in addition to other variant characteristics that indicate GoFs. We developed a variant score, referred to as the ADH1 score, within the UKB cohort that corresponded to the strength of a variant's association with ADH1 phenotypes and relevant variant characteristics (Figures 1A and S1).

The ADH1 score effectively distinguished synonymous variants from ADH1-associated variants in the UKB self-reported White British cohort (0.02 vs. 5.14, $p = 2.2\text{E}{-33}$; Figure 1B). Based on an empirical distribution generated by resampling sub-scores, a threshold of 1.5 was selected, yielding a specificity of 0.98 and a sensitivity of 0.75 in the same cohort, with similar performance observed across other self-reported ethnicities (Tables S3 and S4).

The ADH1 score was applied to 384 rare (minor-allele frequency [MAF] < 0.01) missense/nonsense/frameshift *CASR* variants across the UKB and 468 such variants in AOU.[4–6] A summary of our analysis is shown in Figure 1D, and a complete table of variants and their scores is provided in Tables S7 and S8. Excluding previously established ADH1- and FHH1-associated variants, 35 variants had a score above 1.5 (Figure 1C) in the UKB. Within AOU, 98 variants had a score above 1.5. Overall, we found that 124 variants scored above the threshold in either the UKB or AOU (Figures 1D, 1E, and S4). There was relatively limited overlap since only 151 rare missense/frameshift/stop gains were detected in both biobanks.

Validating the ADH1 score in the MGB Biobank

898 *CASR* variants were identified in the MGB Biobank ($n = 39{,}081$). 101 and 112 were rare (MAF < 0.01) missense/stop-gain variants that were also found in the UKB and AOU, respectively. For each of these variants, we estimated the effect on serum calcium and stratified

Table 2. Characteristics of variants previously associated with ADH1 detected in AOU

						Calcium			Phosphate			Medications				
Variant	Ancestry	No. het	No. hom	Frequency in biobank	MAF in ancestry	Mean (mM)	Beta (mM)	*p* value	Mean (mM)	Beta (mM)	*p* value	No. users	Beta	*p* value	Medications used	Diagnoses
c.310G>A (p.Val104Ile)[a]	AMR-like	1	0	8.70E−06	1.20E−05	2.14	−0.18	0.09	1.26	0.15	0.52	1	3.64	1.40E−10[b]	calcitriol, calcium carbonate, calcium citrate, cholecalciferol	epilepsy: inf, *0.02*[b]; hypoparathyroidism: inf, 2.6E−03[b]
	EUR-like	1	0	8.70E−06	3.90E−06	2.07	−0.23	*0.02*	N/A	N/A	N/A	0	N/A	N/A	N/A	hypocalcemia: inf, 0.01[b]
c.452C>T (p.Thr151Met)[a]	AMR-like	1	0	4.30E−06	1.20E−05	1.66	−0.65	3.20E−09[b]	1.42	0.29	0.21	1	0.55	0.34	calcium carbonate	N/A
c.613C>T (p.Arg205Cys)	AFR-like	4	0	3.00E−05	3.90E−05	2.45	0.13	0.09	1.02	−0.1	0.49	1	0.05	0.85	cholecalciferol	N/A
	EAS-like	3	0	3.00E−05	2.70E−04	2.4	0.08	0.46	N/A	N/A	N/A	1	0.23	0.42	cholecalciferol	N/A
c.1810G>A (p.Glu604Lys)	EUR-like	1	0	4.30E−06	3.90E−06	2.45	0.12	0.23	N/A	N/A	N/A	0	N/A	N/A	N/A	N/A
c.2431A>G (p.Met811Val)[a]	EAS-like	1	0	4.30E−06	9.00E−05	2.07	−0.23	0.03[b]	1.35	0.22	0.32	1	1.58	1.10E−03[b]	calcium carbonate, cholecalciferol	hypocalcemia: inf, 8.1e−03[b]
c.2443_2445del (p.Phe815del)[a]	EUR-like	2	0	8.70E−06	7.80E−06	2.03	−0.29	4.70E−03[b]	N/A	N/A	N/A	0	N/A	N/A	N/A	N/A
c.2503G>A (p.Ala835Thr)[a]	EUR-like	1	0	4.30E−06	3.90E−06	1.97	−0.34	1.10E−03[b]	1.37	0.28	0.15	1	4.49	1.90E−11[b]	calcitriol, calcium carbonate, calcium citrate, cholecalciferol, teriparatide	abnormal involuntary movements: inf, 0.03[b]; disorders of calcium/ phosphorus metabolism: inf, 7.5e−03[b]; epilepsy: inf, 0.03[b]; hypocalcemia: inf, 0.01[b]; hypoparathyroidism: inf, 3.7e−03[b]
c.2800C>T (p.Gln934Ter)[a]	AFR-like	1	0	4.30E−06	9.80E−06	1.97	−0.36	7.20E−04[b]	N/A	N/A	N/A	0	N/A	N/A	N/A	N/A

Bonferroni significance threshold was 5.0E−3 for calcium and diagnoses, 1E−2 for phosphate, and 8.3E−3 for medications. Lower limit of normal (LLN) calcium is 2.2 mM, and upper limit of normal (ULN) phosphate is 1.45 mM. AFR, African ancestry; AMR, American ancestry; EAS, East Asian ancestry; EUR, European ancestry; het, heterozygous; hom, homozygous; MAF, minor-allele frequency.

[a]At least one nominally significant association.

[b]$p < 0.05$.

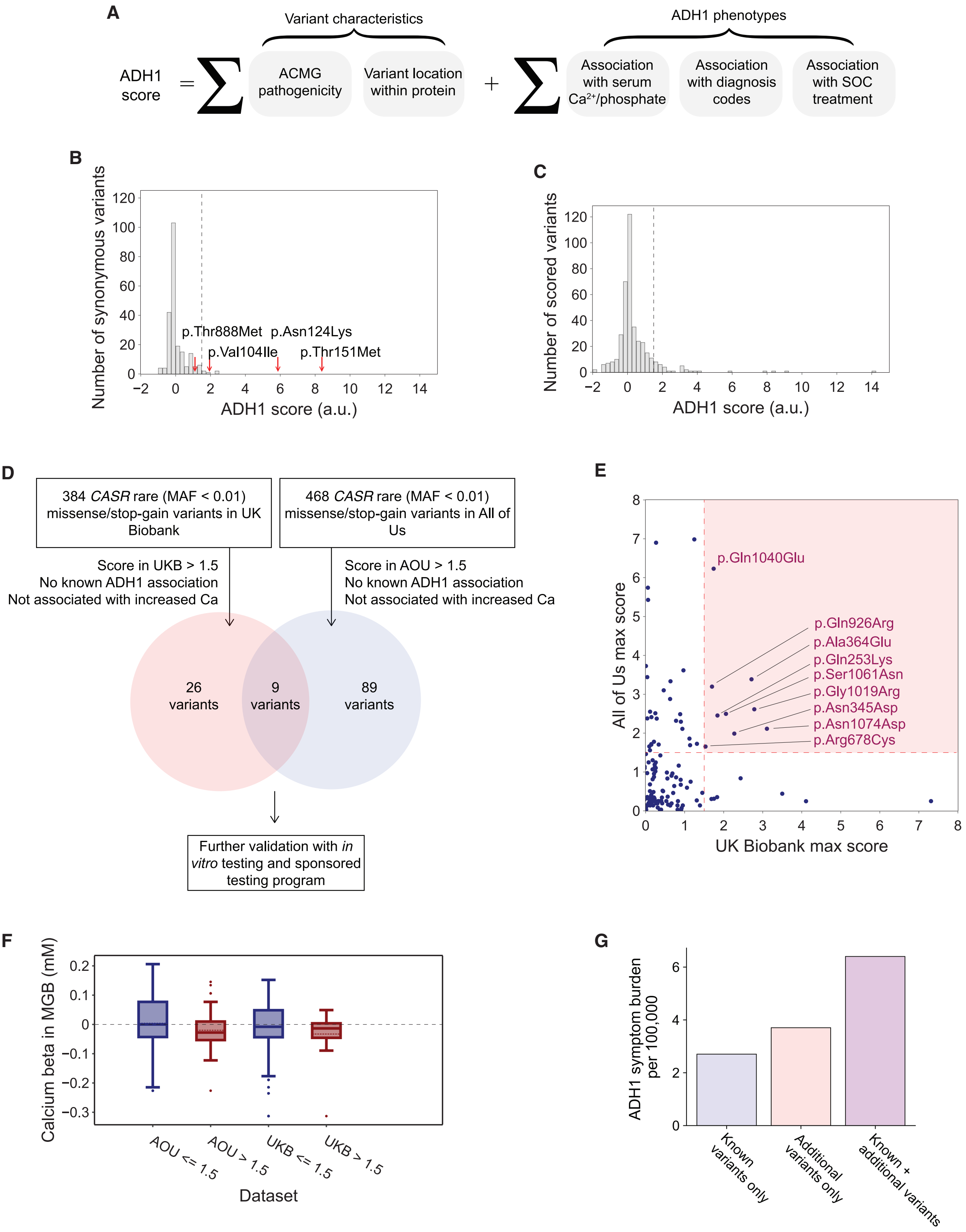

Figure 1. A scoring method to identify variants associated with ADH1

(A) The ADH1 score for each variant and each self-reported ethnicity in the UKB or genetically determined ancestry in AOU was calculated as the weighted sum of sub-scores corresponding to strength of association with ADH1 phenotypes and other variant characteristics. Variant location had a positive sub-score when variants were within known hotspots of GoF variants (aa positions 116–136 and 819–837). SOC treatment, standard-of-care treatment, e.g., calcium supplementation or parathyroid hormone.

(legend continued on next page)

the variants based on the ADH1 score computed in the UKB and AOU, respectively. The median effect on serum calcium was more negative for variants with ADH1 scores >1.5 than those with ADH1 scores $\leq$1.5, which was statistically significant for the AOU-based score and directionally consistent for the UKB-based score, providing independent evidence for the utility of the ADH1 score (t test; AOU: p = 4.6E−2 and UKB: p = 1.3E−1; Figure 1F). Additionally, the majority of variants had the same effect direction when present in the MGB Biobank (Figure S5; Table S9).

Identification of nine additional variants with ADH1 associations in both the UKB and AOU

There were nine variants that scored >1.5 in both biobanks (Tables 3 and S10; a systematic review of the location and structural modeling of the variants is provided in Table S5, and a structural analysis of the variants is shown in Figure S2). c.757C>A (p.Gln253Lys) was associated with relevant medications in the UKB meta-analysis (p = 6.70E−06), nominally associated with partial epilepsy (p = 0.02), and suggestively associated with disturbance of skin sensation (p = 0.08) in the AOU AMR-like cohort. c.1033A>G (p.Asn345Asp) was associated with relevant medications in the UKB meta-analysis (p = 1.40E−67) and tetany in the UKB self-reported Black African cohort (p = 3.5E−03). It was also nominally associated with partial epilepsy in the AOU AFR-like cohort (p = 0.04). c.1091C>A (p.Ala364Glu) was associated with mildly reduced serum calcium levels in the UKB self-reported White British cohort (p = 1.20E−04) and nominally associated with hypoparathyroidism (p = 0.05) and suggestively associated with disorders of calcium/phosphorus metabolism (p = 0.10) within the AOU EUR-like cohort.

c.2032C>T (p.Arg678Cys) was nominally associated with paresthesia in the UKB self-reported White British cohort (p = 0.03) and abnormal involuntary movements in the AOU AFR-like cohort (p = 0.04). c.2777A>G (p.Gln926Arg) was associated with a mild decrease of serum calcium (p = 0.03) and a mild increase of serum phosphate (2.40E−03) in the UKB self-reported White British cohort. It was also associated with relevant medications across the UKB self-reported Indian cohort (p = 5.40E−04) and two ancestries in AOU (AFR-like, p = 3.00E−03, and EUR-like, p = 4.50E−03) and was associated with paresthesia in the UKB self-reported White British cohort (p = 0.05). It was suggestively associated with hypocalcemia in the AOU AFR-like cohort (p = 0.07) and with abnormal involuntary movements (p = 0.05) and generalized convulsive epilepsy (p = 0.09) in the AOU EUR-like cohort. c.3055G>A (p.Gly1019Arg) was nominally associated with disorders of calcium or phosphorous metabolism (p = 0.03) in the UKB self-reported Caribbean cohort as well as with chronic kidney disease in the UKB self-reported White British cohort (p = 0.01). In AOU, it was nominally associated with increased serum phosphate in the AOU AFR-like cohort (p = 0.03) and suggestively associated with abnormal involuntary movements (p = 0.10).

c.3118C>G (p.Gln1040Glu) was nominally associated with diagnosis of disorder of calcium or phosphorous metabolism in the UKB self-reported Caribbean cohort (p = 0.02), and it was also nominally associated with abnormal involuntary movements (p = 0.03) and disorders of calcium/phosphorus metabolism (p = 6.4E−03) and suggestively associated with epilepsy (p = 0.06) and epilepsy, recurrent seizures, and convulsions (p = 0.05) in the AOU AFR-like cohort. c.3182G>A (p.Ser1061Asn) was nominally associated with disorders of calcium or phosphorous in the UKB White British cohort (p = 9.6E−03) and was associated with relevant medications (p = 4.40E−04) and nominally associated with disturbance of skin sensation (p = 0.04) in the AOU SAS-like cohort.

c.3220A>G (p.Asn1074Asp) was associated with hypoparathyroidism in the UKB self-reported Black African cohort (p = 2.6E−03), nominally associated with hypoparathyroidism in the AOU EUR-like cohort (p = 0.03), and suggestively associated with epilepsy (p = 0.08) and epilepsy, recurrent seizures, and convulsions (p = 0.07) in the AOU AFR-like cohort (Tables 3 and S10).

Validation of a subset of these variants was conducted through *in vitro* experiments testing for extracellular sensitivity to calcium (Figure 2; Table S11; supplemental methods). We exogenously expressed seven *CASR* variants (including a known GoF variant, c.310G>A [p.Val104Ile], and a LoF [FHH1] variant, c.220A>C [p.Met74Leu]) in HEK293 cells to determine their response to extracellular calcium. Of the five variants tested, two had ADH1 scores above the threshold in both the UKB and AOU (c.1091C>A [p.Ala364Glu] and c.3220A>G [p.Asn1074Asp]), and two only had ADH1 scores in the UKB (and were not

(B) Comparison of synonymous variants (bars) and previously described ADH1-associated variants (red arrows) shows separation of their scores.
(C) Distribution of all scored variants (missense, frameshift, or nonsense).
(D) Summary of biobank scoring analysis.
(E) Comparison of ADH1 scores of variants found in both the UKB and AOU. Highlighted region indicates variants that scored higher than the 1.5 threshold in both the UKB and AOU.
(F) Boxplot comparing effects on serum calcium, estimated in the MGB Biobank, of variants that scored above/below the threshold of 1.5 in AOU and the UKB. The boxes represent the interquartile range (IQR), the whiskers extend to 1.5 times the IQR, and the dots are outside of this range. The dashed line indicates the mean.
(G) Bar chart showing the symptom burden of ADH1 (per 100,000 individuals in the UKB) from known variants, additional variants, and the combined total.
ADH1, autosomal dominant hypocalcemia type 1; AOU, All of Us; a.u., arbitrary units; MGB, Mass General Brigham; SOC, standard of care; UKB, UK Biobank.

Table 3. Variants that scored above threshold in both UKB and AOU

Variant	Biobank	Ethnicity or Ancestry	No. het	No. hom	MAF in ethnicity or ancestry	ADH1 score	Calcium			Phosphate			Meds	
							Mean (mM)	Beta (mM)	*p* value	Mean (mM)	Beta (mM)	*p* value	Beta	*p* value
c.757C>A (p.Gln253Lys)	UKB	Black African	3	0	1.80E−05	−0.02	2.38	−8.10E−03	0.91	1.18	0.03	0.81	−0.01	0.85
	UKB	Caribbean	10	0	1.80E−05	1.84	2.33	−0.06	0.18	1.23	0.07	0.31	−0.02	0.6
	AOU	AFR-like	40	0	3.90E−04	−0.15	2.3	−0.02	0.51	1.08	−0.03	0.7	0.05	0.58
	AOU	AMR-like	8	0	9.80E−05	2.45	2.39	0.07	0.1	0.9	−0.21	0.37	0.14	0.48
c.1033A>G (p.Asn345Asp)	UKB	Black African	1	0	1.10E−06	2.27	2.53	0.14	0.16	1.17	−9.30E−04	1	−0.03	0.78
	AOU	AFR-like	8	0	7.80E−05	0.95	2.27	−0.04	0.35	1.06	−0.05	0.64	−0.07	0.72
	AOU	EUR-like	4	0	1.60E−05	1.99	2.18[a]	−0.13	0.09	1.05	−0.01	0.92	−0.2	0.55
c.1091C>A (p.Ala364Glu)	UKB	White British	114	0	1.30E−04	2.71	2.34	−0.04	1.20E−04[b]	1.18	0.02	0.16	−0.02	0.18
	AOU	EUR-like	14	0	5.50E−05	3.39	2.33	0.02	0.53	1.28	0.19	0.16	0.05	0.76
c.2032C>T (p.Arg678Cys)	UKB	White British	3	0	5.30E−06	1.53	2.46	0.08	0.14	1.19	0.06	0.51	−0.02	0.84
	AOU	AFR-like	3	0	2.90E−05	1.65	2.32	0.02	0.88	1.19	0.04	0.83	N/A	N/A
	AOU	EUR-like	3	0	1.20E−05	0.19	2.19[a]	−0.12	0.09	0.82	−0.24	0.22	0.2	0.61
c.2777A>G (p.Gln926Arg)	UKB	White British	76	0	9.20E−05	1.7	2.35	−0.03	0.03[b]	1.2	0.06	2.40E−03[b]	−2.20E−03	0.89
	UKB	Indian	2	0	9.20E−05	0.11	2.4	N/A	N/A	1.08	N/A	N/A	−2.50E−16	5.40E−04[b]
	AOU	AFR-like	1	0	9.80E−06	2.23	2.38	0.06	0.58	1.44	0.34	0.11	1.62	3.00E−03[b]
	AOU	AMR-like	5	0	6.10E−05	3.18	2.17[a]	−0.12	0.06	1.41	0.32	0.17	−0.04	0.88
	AOU	EUR-like	58	0	2.30E−04	3.2	2.28	−0.03	0.12	1.13	0.05	0.25	0.25	4.50E−03[b]
c.3055G>A (p.Gly1019Arg)	UKB	Caribbean	3	0	1.70E−05	2.78	2.27	−0.13	0.08	0.93	−0.21	0.07	−0.02	0.8
	AOU	White British	12	0	1.70E−05	0.85	2.4	0.01	0.65	1.14	−0.04	0.41	−0.02	0.6
	AOU	AFR-like	7	0	6.80E−05	2.61	2.32	0.01	0.84	1.57[c]	0.46	0.03[b]	N/A	N/A
	AOU	EUR-like	3	0	1.20E−05	1.23	2.27	−0.05	0.51	1.09	0.05	0.72	0.33	0.39
c.3118C>G (p.Gln1040Glu)	UKB	Caribbean	2	0	2.10E−06	1.74	2.46	0.07	0.48	1.35	0.23	0.15	−9.00E−03	0.92
	AOU	AFR-like	2	0	2.00E−05	6.23	2.31	1.00E−03	0.99	1.3	0.2	0.34	N/A	N/A
	AOU	AMR-like	1	0	1.20E−05	−0.02	2.38	0.07	0.55	N/A	N/A	N/A	N/A	N/A
c.3182G>A (p.Ser1061Asn)	UKB	White British	1	0	1.10E−06	2.06	N/A	N/A	N/A	N/A	N/A	N/A	−0.03	0.8
	AOU	SAS-like	1	0	1.60E−04	2.5	2.32	0.02	0.8	N/A	N/A	N/A	1.71	4.40E−04[b]

(*Continued on next page*)

Table 3. Continued

Variant	Biobank	Ethnicity or Ancestry	No. het	No. hom	MAF in ethnicity or ancestry	ADH1 score	Calcium			Phosphate			Meds	
							Mean (mM)	Beta (mM)	*p* value	Mean (mM)	Beta (mM)	*p* value	Beta	*p* value
c.3220A>G (p.Asn1074Asp)	UKB	Black African	3	0	7.80E−05	3.11	2.32	−0.02	0.8	1.26	0.08	0.51	−0.03	0.63
	UKB	White British	58	0	7.80E−05	3.01	2.36	−0.02	0.12	1.17	0.01	0.62	1.80E−03	0.92
	AOU	AFR-like	3	0	2.90E−05	2.11	2.13[a]	−0.16	0.15	1.23	0.1	0.63	N/A	N/A
	AOU	AMR-like	1	0	1.20E−05	0.57	2.32	0.04	0.75	N/A	N/A	N/A	N/A	N/A
	AOU	EUR-like	7	0	2.70E−05	1.93	2.24	−0.07	0.24	0.84	−0.21	0.28	−0.09	0.72
	AOU	MID-like	1	0	5.50E−04	1.33	2.16[a]	−0.14	0.19	1.21	0.2	0.32	0.43	0.47

In the UKB, Bonferroni significance thresholds were 7.1E−3 for calcium and phosphate and 6.2E−3 for medications. In AOU, Bonferroni significance threshold was 5.0E−3 for calcium and diagnoses, 1E−2 for phosphate, and 8.3E−3 for medications. Lower limit of normal (LLN) calcium is 2.2 mM, and upper limit of normal (ULN) phosphate is 1.45 mM. c.1091C>A (p.Ala364Glu) had an *in vitro* assay *p* < 5E−2 and c.3220A>G (p.Asn1074Asp) had an *in vitro* assay *p* = 7E−2, and both variants were detected in the sponsored testing program. The ethnicity or ancestry column contains self-reported ethnicities for the UKB and genetically determined ancestry for AOU. ADH1, autosomal dominant hypocalcemia; AFR, African ancestry; AMR, American ancestry; EAS, East Asian ancestry; EUR, European ancestry; het, heterozygous; hom, homozygous; MAF, minor-allele frequency; meds, medications; MID, Middle Eastern ancestry.

[a]Below LLN.

[b]$p < 0.05$.

[c]Above ULN.

found in AOU, c.2471C>G [p.Ala824Gly] and c.260T>C [p.Leu87Pro]), or did not score above the threshold in AOU, c.740C>T (p.Ser247Phe). Three of the five variants showed nominally significantly increased sensitivity to extracellular calcium (c.740C>T [p.Ser247Phe], c.1091C>A [p.Ala364Glu], and c.2471C>G [p.Ala824Gly], $p < 0.05$), and a fourth variant showed a suggestive increase in sensitivity (c.3220A>G [p.Asn1074Asp], $p = 0.07$). c.260T>C (p.Leu87Pro) showed no significant changes. Interestingly, c.3220A>G (p.Asn1074Asp) and c.1091C>A (p.Ala364Glu) were also observed in individuals enrolled in a genetic testing program for genetic hypoparathyroidism. Moreover, a pathogenic variant, likely pathogenic variant, or variant of uncertain significance was identified in *CASR* for more than half (56.3%) of individuals with suspected genetic hypoparathyroidism, comprising the largest genetic subset of the genes tested (see Table S12 and supplemental methods for a full list of variants).

Frequency and disease burden of ADH1-associated variants

Interestingly, the nine variants identified using the ADH1 score were found at 12 times higher frequency than all previously established ADH1-associated variants combined (8.3E−5 vs. 6.9E−6; Tables 3, S10, and S13). These variants also had lower odds ratios for ADH1 phenotypes than previously established ADH1-associated variants (Tables 1 and 2).

To more accurately estimate the symptom burden due to these variants, we determined the excess number of individuals with any diagnosed ADH1 phenotype among individuals with an ADH1 variant in the UKB (Tables 3, S10, and S14). Across the nine variants, we found an excess of 14 individuals over the baseline with any ADH1 phenotype (3.7:100,000 in the UKB), which was similar to the frequency of previously reported variants in the UKB (2.7:100,000) (Figure 1G).

Identification of putative phenotypes associated with GoF *CASR* variants

Because individuals with ADH1 have largely been ascertained based on the clinical phenotypes of individuals suspected to have genetic hypoparathyroidism, we sought to take a genetics-first approach to expanding the phenotypic spectrum of ADH1. A phenome-wide association study (pheWAS) through SKAT-O[34] was conducted using both previously reported ($n = 4$) and additionally identified ($n = 9$) ADH1-associated variants in the White British cohort of the UKB across 1,518 phenotypes (Figure S6; Tables S15 and S16).

We recovered associations with abnormal reflex ($p = 3.94E{-}27$) and cardiac conduction disorder ($p = 1.48E{-}18$), both of which are linked to known symptoms of ADH1. Abnormal reflex may be related to tetany, and a prolonged QT interval has been reported in ADH1.[4] Calcium plays an essential role in the heart,[47] and hypocalcemia has generally been linked to QT prolongation[48] and

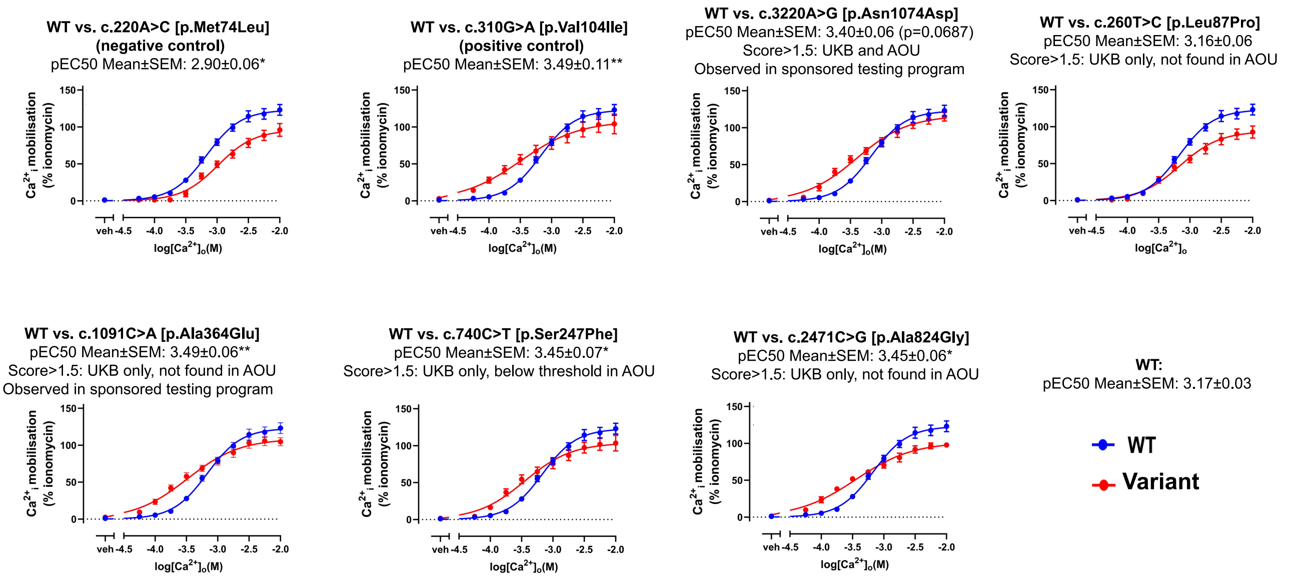

Figure 2. ***In vitro*** **functional assay to characterize variant sensitivity to extracellular calcium**
HEK293 cells expressing each *CASR* variant were exposed to various concentrations of extracellular calcium, and calcium signaling activity was determined using Fluo-8 AM. c.220A>C (p.Met74Leu) was a negative control, and c.310G>A (p.Val104Ile) was a positive control. Each experiment was run 5 times. $*p < 0.05$ and $**p < 0.01$. p values are not reported for tests that are not statistically significant or near significance.

arrhythmia.[49] Associations with coagulation defects and bone and tooth disorders were also detected, all of which have plausible connections to calcium homeostasis, as many of the proteins in the coagulation cascade are calcium dependent.[50] Furthermore, other associations included rickets or osteomalacia ($p = 7.34E-10$), as well as hereditary disturbances in tooth structure ($p = 6.26E-17$). This was consistent with reported associations with mutations in the vitamin D receptor and hypocalcemic rickets[51] and dental symptoms in persons with idiopathic hypoparathyroidism.[52]

CASR variants constitute an allelic series on serum calcium

Multiple independent common variants in *CASR* are also associated with serum calcium levels.[53] We conducted fine mapping of these common variants using SuSiE[54] and identified 10 independent causal sets that were distinct from ADH1-associated variants (Table S17). Additionally, we compared their frequency and effect size against the nine variants identified using the ADH1 score, as well as variants previously associated with ADH1 (Figures 3 and S7). As expected, we show that the impact on serum calcium grew more negative as the variant frequency decreased, suggesting *CASR* function as a central lever for calcium homeostasis in humans.

Discussion

Through our analysis of two large-scale biobanks, we found that the symptom burden of ADH1 is at least 2-fold greater than previously believed.[9,55] We developed an ADH1 variant score that identified five ADH1-associated variants with intermediate effect and frequency. The identified variants show a statistically significant but smaller effect on serum calcium, which can be explained by a combination of lower penetrance and variable expressivity. In other words, a larger proportion of individuals with these variants have sub-clinical phenotypes than traditional ADH1 variants, but individuals in the clinical range show overt clinical symptoms, making these variants more difficult to identify as ADH1 associated. These variants complete an allelic series of *CASR* by bridging the gap between common genome-wide association study (GWAS) variants and rare familial variants (a GoF causing hypocalcemia and a LoF causing hypercalcemia). This allelic series suggests that there is a dose-responsive relationship between *CASR* activity and serum calcium levels. However, we note that the pEC50 (negative logarithm of the EC50, which stands for half-maximal effective concentration) observed in *in vitro* assays did not correlate directly with the penetrance of each variant. The assay only captures one dimension of calcium receptor activity that can be modulated in different ways, and pEC50 is just one dimension of the assay itself, as shown in the complex shape of calcium mobilization. This effect would need to be considered in the context of other factors to quantitatively measure the biochemical activity of CaSR.

Individuals with previously reported variants were hypocalcemic (63% in the UKB and 89% in AOU), but less than half had a diagnosis code consistent with ADH1 (17% in the UKB and 44% in AOU), which are rates lower than previously described in clinically ascertained cohorts.[4] In

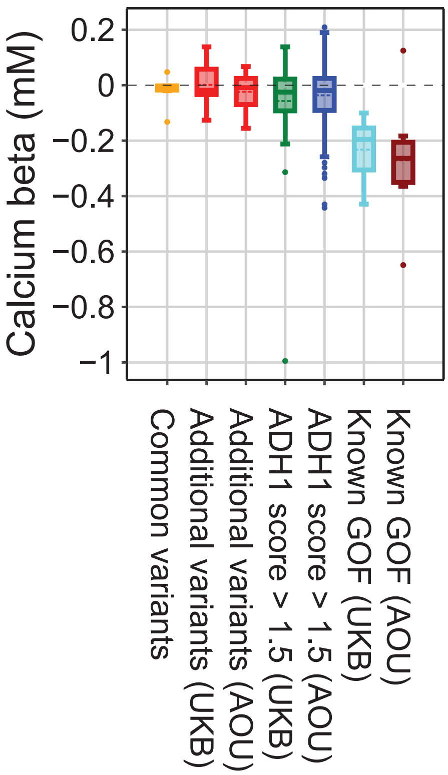

Figure 3. Genetic architecture of serum calcium with respect to *CASR* variation
Boxplot of calcium effects for sets of variants. The boxes represent the interquartile range (IQR), the whiskers extend to 1.5 times the IQR, and the dots are outside of this range. Additional variants are those that scored >1.5 in both the UKB and AOU (as presented in Tables 3 and S10). The ADH1 score >1.5 categories include the additional variants as well as all others that met the score criteria for each biobank.
ADH1, autosomal dominant hypocalcemia type 1; AOU, All of Us; IQR, interquartile range; UKB, UK Biobank.

principle, our approach provides a complementary data point to the classical phenotype-first penetrance of ADH1 variants. Nevertheless, even based on this lower-bound penetrance, we suggest a symptomatic prevalence of 0.3:100,000–2.0:100,000, purely due to previously reported ADH1 variants, and the nine additional variants alone contribute to an additional symptom burden of 3.7:100,000. This figure only accounts for symptom burden due to variants observed in both biobanks and is likely still an underestimate of the true ADH1 symptom burden.

The higher-than-expected genetics-based prevalence of monogenic disorders is consistent with recent reports in *NOTCH3*-associated monogenic stroke (MIM: 125310),[56] monogenic developmental disorders,[57] and other dominant disorders,[58,59] highlighting an important but underrecognized role of genetic variants underlying disease variability in the general population. These studies consistently showed an increased prevalence of dominant pathogenic variants but significantly smaller numbers of diagnosed individuals than expected, suggesting lower penetrance and greater variability in expressivity that may be modified by other genetic factors, environmental factors, or simply stochasticity in these seemingly monogenic disorders.[56,60,61] Future work could consider environmental factors (e.g., diet) or a genome-wide or pathway-based polygenic score (e.g., incorporating variants in other genes involved in calcium homeostasis such as *VDR* [MIM: 601769], *CYP24A1* [MIM: 126065], *CLDN14* [MIM: 605608], *GNA11* [MIM: 139313], and *PTH1R* [MIM: 168468][62]) to further understand modifiers of *CASR* variant penetrance.

The framework that we developed to identify novel ADH1 associations may serve as or could be developed further into a blueprint to use for other monogenic diseases. Our work highlights the potentially underrecognized symptom burden driven by these genes. However, even with a sample size of ∼750,000, the number of individuals with known ADH1 variants was limited ($n < 30$); thus, we were underpowered to build a hypothesis-free model. Through ever-growing cohorts with diverse inclusion criteria, we anticipate that further improvements to this framework will deepen our understanding of the true burden and variable expressivity of variants in genes associated with rare monogenic disorders.

Acknowledgments

We thank all participants of the UKB, AOU, and MGB Biobank, as this study would not have been possible without their contributions. We also thank the NIH's AOU Research Program, the UKB resource (under application number 62375), and the MGB team for making available the participant data examined in this study. J.B.C. thanks K. Shen for her support and feedback. T.M.J. is a National Health and Medical Research Council Fellow (2008341). This work was funded by BridgeBio Pharma.

Author contributions

Conceptualization, J.B.C. and S.-G.J.; formal analysis, J.B.C., M. M.S., A.P., C.P.B., A.M.A., B.W.D., J.H., Q.C., J.L.N., X.Z., and C.M.G.; investigation, J.B.C., M.M.S., A.P., C.P.B., A.M.A., B.W. D., J.H., L.M.S.S., A.S.M., J.L.N., J.D., C.D.F., Q.C., T.M.J., and C.M.G.; resources, L.M.S.S., A.S.M., and S.-G.J.; data curation, J.B.C., M.M.S., A.P., X.Z., and C.P.B.; writing – original draft, J.B.C. and S.-G.J.; writing – review & editing, C.L., R.A., T.M.J., J.A.L.-S., C.M.G., M.S.R., S.H.A., J.C.F., J.L.N., X.Z., J.B.C., and S.-G.J.; visualization, J.B.C.; supervision, J.B.C. and S.-G.J.; project administration, J.B.C. and S.-G.J.

Declaration of interests

J.B.C., M.M.S., X.Z., C.P.B., J.L.N., L.M.S.S., A.S.M., A.P., M.S.R., S. H.A., J.C.F., and S.-G.J. are current or former employees and shareholders of BridgeBio Pharma. B.W.D., C.L., and R.A. are consultants of BridgeBio Pharma.

Supplemental information

Supplemental information can be found online at https://doi.org/10.1016/j.ajhg.2025.06.013.

Web resources

Coloc SuSiE, https://chr1swallace.github.io/coloc/articles/a06_SuSiE.html
GenBank, https://www.ncbi.nlm.nih.gov/genbank/
Genotype-Tissue Expression (GTEx) Project, https://www.gtexportal.org/home/
HAIL v.0.2.78, https://github.com/hail-is/hail
OMIM, https://www.omim.org
Owlready2, https://owlready2.readthedocs.io/en/latest/index.html#
Phecode Map 1.2, https://phewascatalog.org/phewas/#home
PLINK v.2.00a3.1LM, www.cog-genomics.org/plink/2.0/
Python statsmodels package v.0.14.1, https://www.statsmodels.org/stable/install.html
SKAT R package v.2.2.5, https://cran.r-project.org/web/packages/SKAT/vignettes/SKAT.pdf
Varsome, https://varsome.com/

Received: November 19, 2024
Accepted: June 20, 2025
Published: July 14, 2025

References

1. Tisdale, A., Cutillo, C.M., Nathan, R., Russo, P., Laraway, B., Haendel, M., Nowak, D., Hasche, C., Chan, C.-H., Griese, E., et al. (2021). The IDeaS initiative: pilot study to assess the impact of rare diseases on patients and healthcare systems. Orphanet J. Rare Dis. *16*, 429. https://doi.org/10.1186/s13023-021-02061-3.
2. Nguengang Wakap, S., Lambert, D.M., Olry, A., Rodwell, C., Gueydan, C., Lanneau, V., Murphy, D., Le Cam, Y., and Rath, A. (2020). Estimating cumulative point prevalence of rare diseases: analysis of the Orphanet database. Eur. J. Hum. Genet. *28*, 165–173. https://doi.org/10.1038/s41431-019-0508-0.
3. Pontén, F., Gry, M., Fagerberg, L., Lundberg, E., Asplund, A., Berglund, L., Oksvold, P., Björling, E., Hober, S., Kampf, C., et al. (2009). A global view of protein expression in human cells, tissues, and organs. Mol. Syst. Biol. *5*, 337. https://doi.org/10.1038/msb.2009.93.
4. Roszko, K.L., Stapleton Smith, L.M., Sridhar, A.V., Roberts, M.S., Hartley, I.R., Gafni, R.I., Collins, M.T., Fox, J.C., and Nemeth, E.F. (2022). Autosomal Dominant Hypocalcemia Type 1: A Systematic Review. J. Bone Miner. Res. *37*, 1926–1935. https://doi.org/10.1002/jbmr.4659.
5. Wu, Y., Zhang, C., Huang, X., Cao, L., Liu, S., and Zhong, P. (2022). Autosomal dominant hypocalcemia with a novel CASR mutation: a case study and literature review. J. Int. Med. Res. *50*, 03000605221110489. https://doi.org/10.1177/03000605221110489.
6. Zung, A., Barash, G., Banne, E., and Levine, M.A. (2023). Novel Calcium-Sensing Receptor (CASR) Mutation in a Family with Autosomal Dominant Hypocalcemia Type 1 (ADH1): Genetic Study over Three Generations and Clinical Characteristics. Horm. Res. Paediatr. *96*, 473–482. https://doi.org/10.1159/000529833.
7. Gorvin, C.M. (2019). Molecular and clinical insights from studies of calcium-sensing receptor mutations. J. Mol. Endocrinol. *63*, R1–R16. https://doi.org/10.1530/jme-19-0104.
8. Gafni, R.I., Hartley, I.R., Roszko, K.L., Nemeth, E.F., Pozo, K.A., Lombardi, E., Sridhar, A.V., Roberts, M.S., Fox, J.C., and Collins, M.T. (2023). Efficacy and Safety of Encaleret in Autosomal Dominant Hypocalcemia Type 1. N. Engl. J. Med. *389*, 1245–1247. https://doi.org/10.1056/nejmc2302708.
9. Dershem, R., Gorvin, C.M., Metpally, R.P.R., Krishnamurthy, S., Smelser, D.T., Hannan, F.M., Carey, D.J., Thakker, R.V., Breitwieser, G.E.; and Regeneron Genetics Center (2020). Familial Hypocalciuric Hypercalcemia Type 1 and Autosomal-Dominant Hypocalcemia Type 1: Prevalence in a Large Healthcare Population. Am. J. Hum. Genet. *106*, 734–747. https://doi.org/10.1016/j.ajhg.2020.04.006.
10. Kingdom, R., and Wright, C.F. (2022). Incomplete Penetrance and Variable Expressivity: From Clinical Studies to Population Cohorts. Front. Genet. *13*, 920390. https://doi.org/10.3389/fgene.2022.920390.
11. Watanabe, S., Fukumoto, S., Chang, H., Takeuchi, Y., Hasegawa, Y., Okazaki, R., Chikatsu, N., and Fujita, T. (2002). Association between activating mutations of calcium-sensing receptor and Bartter's syndrome. Lancet *360*, 692–694. https://doi.org/10.1016/s0140-6736(02)09842-2.
12. Sudlow, C., Gallacher, J., Allen, N., Beral, V., Burton, P., Danesh, J., Downey, P., Elliott, P., Green, J., Landray, M., et al. (2015). UK Biobank: An Open Access Resource for Identifying the Causes of a Wide Range of Complex Diseases of Middle and Old Age. PLoS Med. *12*, e1001779. https://doi.org/10.1371/journal.pmed.1001779.
13. Backman, J.D., Li, A.H., Marcketta, A., Sun, D., Mbatchou, J., Kessler, M.D., Benner, C., Liu, D., Locke, A.E., Balasubramanian, S., et al. (2021). Exome sequencing and analysis of 454,787 UK Biobank participants. Nature *599*, 628–634. https://doi.org/10.1038/s41586-021-04103-z.
14. Harrison, P.W., Amode, M.R., Austine-Orimoloye, O., Azov, A.G., Barba, M., Barnes, I., Becker, A., Bennett, R., Berry, A., Bhai, J., et al. (2024). Ensembl 2024. Nucleic Acids Res. *52*, D891–D899. https://doi.org/10.1093/nar/gkad1049.
15. Chang, C.C., Chow, C.C., Tellier, L.C., Vattikuti, S., Purcell, S.M., and Lee, J.J. (2015). Second-generation PLINK: rising to the challenge of larger and richer datasets. GigaScience *4*, s13742-015-0047–0048. https://doi.org/10.1186/s13742-015-0047-8.
16. Ganna, A., Genovese, G., Howrigan, D.P., Byrnes, A., Kurki, M., Zekavat, S.M., Whelan, C.W., Kals, M., Nivard, M.G., Bloemendal, A., et al. (2016). Ultra-rare disruptive and damaging mutations influence educational attainment in the general population. Nat. Neurosci. *19*, 1563–1565. https://doi.org/10.1038/nn.4404.
17. Inker, L.A., Schmid, C.H., Tighiouart, H., Eckfeldt, J.H., Feldman, H.I., Greene, T., Kusek, J.W., Manzi, J., Van Lente, F., Zhang, Y.L., et al. (2012). Estimating Glomerular Filtration Rate from Serum Creatinine and Cystatin C. N. Engl. J. Med. *367*, 20–29. https://doi.org/10.1056/nejmoa1114248.
18. Bastarache, L. (2021). Using Phecodes for Research with the Electronic Health Record: From PheWAS to PheRS. Annu. Rev. Biomed. Data Sci. *4*, 1–19. https://doi.org/10.1146/annurev-biodatasci-122320-112352.
19. All of Us Research Program Investigators, Denny, J.C., Rutter, J.L., Goldstein, D.B., Philippakis, A., Smoller, J.W., Jenkins, G., Dishman, E., and Dishman, E. (2019). The "All of Us" Research Program. N. Engl. J. Med. *381*, 668–676. https://doi.org/10.1056/nejmsr1809937.
20. Lamy, J.-B. (2017). Owlready: Ontology-oriented programming in Python with automatic classification and high level

constructs for biomedical ontologies. Artif. Intell. Med. *80*, 11–28. https://doi.org/10.1016/j.artmed.2017.07.002.
21. Hannan, F.M., Nesbit, M.A., Zhang, C., Cranston, T., Curley, A.J., Harding, B., Fratter, C., Rust, N., Christie, P.T., Turner, J. J.O., et al. (2012). Identification of 70 calcium-sensing receptor mutations in hyper- and hypo-calcaemic patients: evidence for clustering of extracellular domain mutations at calcium-binding sites. Hum. Mol. Genet. *21*, 2768–2778. https://doi.org/10.1093/hmg/dds105.
22. Hu, J., Mora, S., Colussi, G., Proverbio, M.C., Jones, K.A., Bolzoni, L., De Ferrari, M.E., Civati, G., and Spiegel, A.M. (2002). Autosomal Dominant Hypocalcemia Caused by a Novel Mutation in the Loop 2 Region of the Human Calcium Receptor Extracellular Domain. J. Bone Miner. Res. *17*, 1461–1469. https://doi.org/10.1359/jbmr.2002.17.8.1461.
23. Schouten, B.J., Raizis, A.M., Soule, S.G., Cole, D.R., Frengley, P.A., George, P.M., and Florkowski, C.M. (2011). Four cases of autosomal dominant hypocalcaemia with hypercalciuria including two with novel mutations in the calcium-sensing receptor gene. Ann. Clin. Biochem. *48*, 286–290. https://doi.org/10.1258/acb.2010.010139.
24. Lienhardt, A., Bai, M., Lagarde, J.-P., Rigaud, M., Zhang, Z., Jiang, Y., Kottler, M.-L., Brown, E.M., and Garabédian, M. (2001). Activating Mutations of the Calcium-Sensing Receptor: Management of Hypocalcemia. J. Clin. Endocrinol. Metab. *86*, 5313–5323. https://doi.org/10.1210/jcem.86.11.8016.
25. Hawkes, C.P., Shulman, D.I., and Levine, M.A. (2020). Recombinant human parathyroid hormone (1–84) is effective in CASR-associated hypoparathyroidism. Eur. J. Endocrinol. *183*, K13–K21. https://doi.org/10.1530/eje-20-0710.
26. Pearce, S.H., Williamson, C., Kifor, O., Bai, M., Coulthard, M. G., Davies, M., Lewis-Barned, N., McCredie, D., Powell, H., Kendall-Taylor, P., et al. (1996). A Familial Syndrome of Hypocalcemia with Hypercalciuria Due to Mutations in the Calcium-Sensing Receptor. N. Engl. J. Med. *335*, 1115–1122. https://doi.org/10.1056/nejm199610103351505.
27. Sørheim, J.I., Husebye, E.S., Nedrebø, B.G., Svarstad, E., Lind, J., Boman, H., and Løvås, K. (2010). Phenotypic Variation in a Large Family with Autosomal Dominant Hypocalcaemia. Horm. Res. Paediatr. *74*, 399–405. https://doi.org/10.1159/000303188.
28. Løvlie, R., Eiken, H.G., Sørheim, J.I., and Boman, H. (1996). The Ca2+-sensing receptor gene (PCAR1) mutation T151M in isolated autosomal dominant hypoparathyroidism. Hum. Genet. *98*, 129–133. https://doi.org/10.1007/s004390050174.
29. Ji, Y., Kang, C., Chen, J., and Zhang, L. (2021). Identification of p.Arg205Cys in CASR in an autosomal dominant hypocalcaemia type 1 pedigree. Medicine *100*, e26443. https://doi.org/10.1097/md.0000000000026443.
30. Lazarus, S., Pretorius, C.J., Khafagi, F., Campion, K.L., Brennan, S.C., Conigrave, A.D., Brown, E.M., and Ward, D.T. (2011). A novel mutation of the primary protein kinase C phosphorylation site in the calcium-sensing receptor causes autosomal dominant hypocalcemia. Eur. J. Endocrinol. *164*, 429–435. https://doi.org/10.1530/eje-10-0907.
31. Hu, J., McLarnon, S.J., Mora, S., Jiang, J., Thomas, C., Jacobson, K.A., and Spiegel, A.M. (2005). A Region in the Seven-transmembrane Domain of the Human Ca2+ Receptor Critical for Response to Ca2+. J. Biol. Chem. *280*, 5113–5120. https://doi.org/10.1074/jbc.m413403200.
32. Kopanos, C., Tsiolkas, V., Kouris, A., Chapple, C.E., Albarca Aguilera, M., Meyer, R., and Massouras, A. (2019). VarSome: the human genomic variant search engine. Bioinformatics *35*, 1978–1980. https://doi.org/10.1093/bioinformatics/bty897.
33. Josephs, T.M., Keller, A.N., Khajehali, E., DeBono, A., Langmead, C.J., Conigrave, A.D., Capuano, B., Kufareva, I., Gregory, K.J., and Leach, K. (2020). Negative allosteric modulators of the human calcium-sensing receptor bind to overlapping and distinct sites within the 7-transmembrane domain. Br. J. Pharmacol. *177*, 1917–1930. https://doi.org/10.1111/bph.14961.
34. Lee, S., Emond, M.J., Bamshad, M.J., Barnes, K.C., Rieder, M. J., Nickerson, D.A., NHLBI GO Exome Sequencing Project—ESP Lung Project Team, Christiani, D.C., Wurfel, M.M., and Lin, X. (2012). Optimal Unified Approach for Rare-Variant Association Testing with Application to Small-Sample Case-Control Whole-Exome Sequencing Studies. Am. J. Hum. Genet. *91*, 224–237. https://doi.org/10.1016/j.ajhg.2012.06.007.
35. Wallace, C. (2021). A more accurate method for colocalisation analysis allowing for multiple causal variants. PLoS Genet. *17*, e1009440. https://doi.org/10.1371/journal.pgen.1009440.
36. Boutin, N.T., Schecter, S.B., Perez, E.F., Tchamitchian, N.S., Cerretani, X.R., Gainer, V.S., Lebo, M.S., Mahanta, L.M., Karlson, E.W., and Smoller, J.W. (2022). The Evolution of a Large Biobank at Mass General Brigham. J. Pers. Med. *12*, 1323. https://doi.org/10.3390/jpm12081323.
37. Goldstein. (1990). Chapter 143. Serum Calcium. In Clinical Methods: The History, Physical, and Laboratory Examinations, W. HK, H. WD, and H. JW, eds.
38. Bazydlo, L.A.L., Needham, M., and Harris, N.S. (2014). Calcium, Magnesium, and Phosphate. Lab. Med. *45*, e44–e50. https://doi.org/10.1309/lmglmz8ciymfnogx.
39. Cole, D.E.C., Yun, F.H.J., Wong, B.Y.L., Shuen, A.Y., Booth, R. A., Scillitani, A., Pidasheva, S., Zhou, X., Canaff, L., and Hendy, G.N. (2009). Calcium-sensing receptor mutations and denaturing high performance liquid chromatography. J. Mol. Endocrinol. *42*, 331–339. https://doi.org/10.1677/jme-08-0164.
40. Gagliardi, L., Burt, M.G., Feng, J., Poplawski, N.K., and Scott, H.S. (2016). Autosomal dominant hypocalcaemia due to a novel CASR mutation: clinical and genetic implications. Clin. Endocrinol. *85*, 495–497. https://doi.org/10.1111/cen.13104.
41. D'Souza-Li, L., Yang, B., Canaff, L., Bai, M., Hanley, D.A., Bastepe, M., Salisbury, S.R., Brown, E.M., Cole, D.E.C., and Hendy, G.N. (2002). Identification and Functional Characterization of Novel Calcium-Sensing Receptor Mutations in Familial Hypocalciuric Hypercalcemia and Autosomal Dominant Hypocalcemia. J. Clin. Endocrinol. Metab. *87*, 1309–1318. https://doi.org/10.1210/jcem.87.3.8280.
42. García-Castaño, A., Madariaga, L., Pérez de Nanclares, G., Ariceta, G., Gaztambide, S., and Castaño, L. (2019). Novel mutations associated with inherited human calcium-sensing receptor disorders: A clinical genetic study. Eur. J. Endocrinol. *180*, 59–70. https://doi.org/10.1530/eje-18-0129.
43. Landrum, M.J., Lee, J.M., Riley, G.R., Jang, W., Rubinstein, W.S., Church, D.M., and Maglott, D.R. (2014). ClinVar: public archive of relationships among sequence variation and human phenotype. Nucleic Acids Res. *42*, D980–D985. https://doi.org/10.1093/nar/gkt1113.

44. Nissen, P.H., Christensen, S.E., Ladefoged, S.A., Brixen, K., Heickendorff, L., and Mosekilde, L. (2012). Identification of rare and frequent variants of the CASR gene by high-resolution melting. Clin. Chim. Acta *413*, 605–611. https://doi.org/10.1016/j.cca.2011.12.004.
45. Taliun, D., Harris, D.N., Kessler, M.D., Carlson, J., Szpiech, Z. A., Torres, R., Taliun, S.A.G., Corvelo, A., Gogarten, S.M., Kang, H.M., et al. (2021). Sequencing of 53,831 diverse genomes from the NHLBI TOPMed Program. Nature *590*, 290–299. https://doi.org/10.1038/s41586-021-03205-y.
46. Karczewski, K.J., Francioli, L.C., Tiao, G., Cummings, B.B., Alföldi, J., Wang, Q., Collins, R.L., Laricchia, K.M., Ganna, A., Birnbaum, D.P., et al. (2020). The mutational constraint spectrum quantified from variation in 141,456 humans. Nature *581*, 434–443. https://doi.org/10.1038/s41586-020-2308-7.
47. Marks, A.R. (2003). Calcium and the heart: a question of life and death. J. Clin. Investig. *111*, 597–600. https://doi.org/10.1172/jci18067.
48. Johnson, J.D., and Jennings, R. (1968). Hypocalcemia and Cardiac Arrhythmias. Am. J. Dis. Child. *115*, 373–376. https://doi.org/10.1001/archpedi.1968.02100010375014.
49. Khalid, S., Albaba, I., and Neu, K. (2023). Hypocalcemia: A Little Known Cause of Supraventricular Tachyarrhythmia. Cureus *15*, e38456. https://doi.org/10.7759/cureus.38456.
50. Furie, B., and Furie, B.C. (1988). The molecular basis of blood coagulation. Cell *53*, 505–518. https://doi.org/10.1016/0092-8674(88)90567-3.
51. Arita, K., Nanda, A., Wessagowit, V., Akiyama, M., Alsaleh, Q.A., and McGrath, J.A. (2008). A novel mutation in the VDR gene in hereditary vitamin D-resistant rickets. Br. J. Dermatol. *158*, 168–171. https://doi.org/10.1111/j.1365-2133.2007.08232.x.
52. Kamarthi, N., Venkatraman, S., and Patil, P.B. (2013). Dental findings in the diagnosis of idiopathic hypoparathyroidism. Ann. Saudi Med. *33*, 411–413. https://doi.org/10.5144/0256-4947.2013.411.
53. Sinnott-Armstrong, N., Tanigawa, Y., Amar, D., Mars, N., Benner, C., Aguirre, M., Venkataraman, G.R., Wainberg, M., Ollila, H.M., Kiiskinen, T., et al. (2021). Genetics of 35 blood and urine biomarkers in the UK Biobank. Nat. Genet. *53*, 185–194. https://doi.org/10.1038/s41588-020-00757-z.
54. Zou, Y., Carbonetto, P., Wang, G., and Stephens, M. (2022). Fine-mapping from summary data with the "Sum of Single Effects" model. PLoS Genet. *18*, e1010299. https://doi.org/10.1371/journal.pgen.1010299.
55. Gunn, I.R., and Gaffney, D. (2004). Clinical and laboratory features of calcium-sensing receptor disorders: a systematic review. Ann. Clin. Biochem. *41*, 441–458. https://doi.org/10.1258/0004563042466802.
56. Cho, B.P.H., Harshfield, E.L., Al-Thani, M., Tozer, D.J., Bell, S., and Markus, H.S. (2022). Association of Vascular Risk Factors and Genetic Factors With Penetrance of Variants Causing Monogenic Stroke. JAMA Neurol. *79*, 1303–1311. https://doi.org/10.1001/jamaneurol.2022.3832.
57. Kingdom, R., Tuke, M., Wood, A., Beaumont, R.N., Frayling, T.M., Weedon, M.N., and Wright, C.F. (2022). Rare genetic variants in genes and loci linked to dominant monogenic developmental disorders cause milder related phenotypes in the general population. Am. J. Hum. Genet. *109*, 1308–1316. https://doi.org/10.1016/j.ajhg.2022.05.011.
58. Kingdom, R., Beaumont, R.N., Wood, A.R., Weedon, M.N., and Wright, C.F. (2024). Genetic modifiers of rare variants in monogenic developmental disorder loci. Nat. Genet. *56*, 861–868. https://doi.org/10.1038/s41588-024-01710-0.
59. Ibañez, K., Jadhav, B., Zanovello, M., Gagliardi, D., Clarkson, C., Facchini, S., Garg, P., Martin-Trujillo, A., Gies, S.J., Deforie, V.G., et al. (2024). Increased frequency of repeat expansion mutations across different populations. Nat. Med. *30*, 1–12. https://doi.org/10.1038/s41591-024-03190-5.
60. Wright, C.F., Sharp, L.N., Jackson, L., Murray, A., Ware, J.S., MacArthur, D.G., Rehm, H.L., Patel, K.A., and Weedon, M.N. (2024). Guidance for estimating penetrance of monogenic disease-causing variants in population cohorts. Nat. Genet. *56*, 1772–1779. https://doi.org/10.1038/s41588-024-01842-3.
61. Forrest, I.S., Chaudhary, K., Vy, H.M.T., Petrazzini, B.O., Bafna, S., Jordan, D.M., Rocheleau, G., Loos, R.J.F., Nadkarni, G.N., Cho, J.H., and Do, R. (2022). Population-Based Penetrance of Deleterious Clinical Variants. JAMA *327*, 350–359. https://doi.org/10.1001/jama.2021.23686.
62. da Silva Lopes, K., and Abe, S.K. (2021). Polymorphisms Contributing to Calcium Status: A Systematic Review. Nutrients *13*, 2488. https://doi.org/10.3390/nu13082488.

The American Journal of Human Genetics, Volume *112*

Supplemental information

A calcium-sensing receptor allelic series and underdiagnosis of genetically driven hypocalcemia

Jeremy B. Chang, Connor P. Barnhill, Alexander M. Apostolov, Marcus M. Soliai, Julian Hecker, Jovia L. Nierenberg, Lyndsay M. Stapleton Smith, Arun S. Mathew, Xue Zeng, Jiayin Diao, C. Dilanka Fernando, Qingwen Chen, Ben W. Dulken, Aleksandr Petukhov, Russ Altman, Tracy M. Josephs, Jessica A. Lasky-Su, Caroline M. Gorvin, Mary Scott Roberts, Scott H. Adler, Jonathan C. Fox, Christoph Lange, and Sun-Gou Ji

Figure S1: Distribution of resampled total scores

Figure S2: Structural analysis of CaSR variants

Figure S3: Serum calcium and phosphate levels in the UK Biobank

Figure S4: Maximum scores in the UKB and AOU

Figure S5: Comparison of serum calcium effects in MGB, AOU, and UKB

Figure S6 Phenome-wide association study using both known and novel ADH1-associated variants

Figure S7: Genetic architecture of serum calcium with respect to CASR variation

Table S3: Sensitivity and specificity in the UK Biobank

Table S4: Sensitivity and specificity in All of Us

Table S6: UKB known ADH1 variants outside of analysis cohort

Table S11: In vitro testing

Table S12: Sponsored testing variants

Table S13: Variant frequencies

Table S14: Symptomatic odds ratios

Table S17: Common variants

Supplemental Methods

References

Figure S1: Distribution of resampled total scores

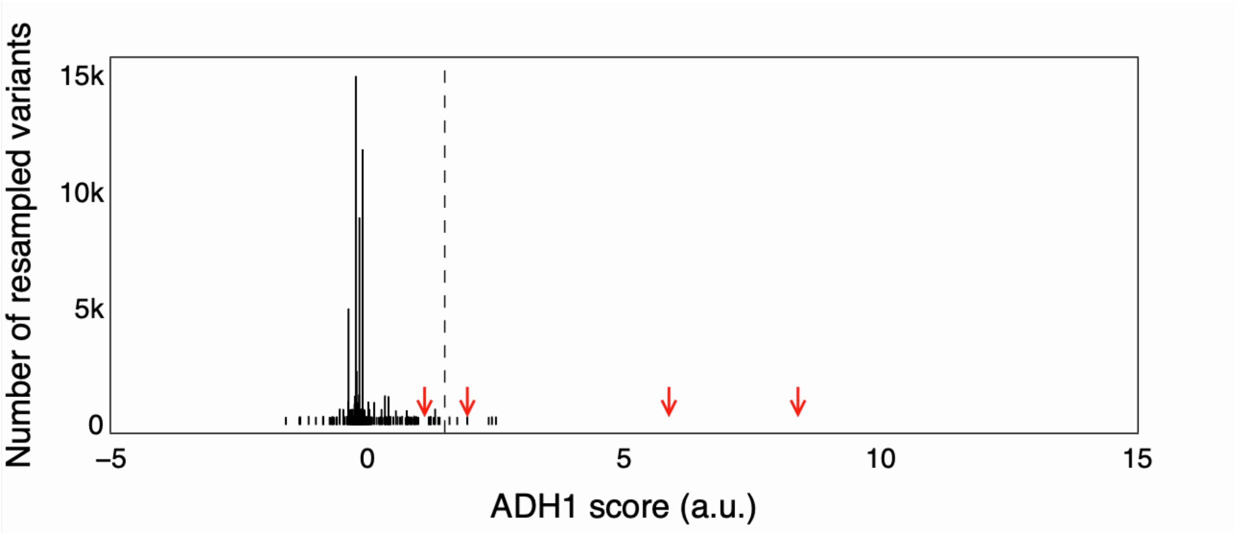

Figure caption: **Distribution of resampled total scores.** Histogram of total scores from the null distribution, which was created by re-sampling scores from synonymous variants. Red arrows indicate values of previously described gain-of-function variants. ADH1=autosomal dominant hypocalcemia type 1; a.u.=arbitrary units.

Figure S2: Structural analysis of CaSR variants

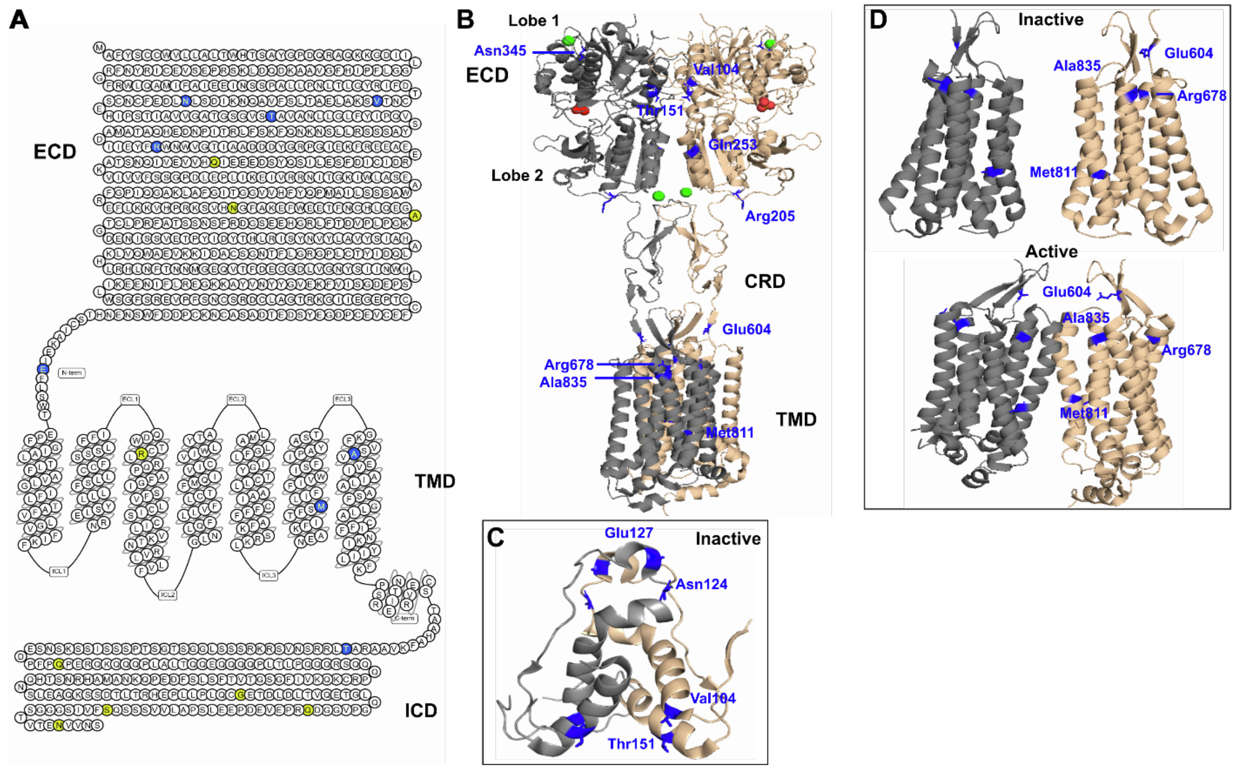

Figure caption. **Structural analysis of CaSR variants.** (A) Snake plot showing the locations of the 14 missense variants identified in Tables 1-3. Tables 1–2 missense variants (blue), Table 3 variants (yellow). ECD, extracellular domain, TMD, transmembrane domain, ICD, intracellular domain. (B) Model of homodimeric CaSR ECD, cysteine-rich domain (CRD) and TMD highlighting the locations of residues where variants were identified. Protomer 1 shown in grey and protomer 2 in light brown. Ca^{2+} shown in green. (C) Zoomed image of the ECD variants in the dimer interface. Several residues project into the dimer interface. (D) Location of the TMD variant residues in the inactive and active state.

Figure S3: Serum calcium and phosphate levels in the UK Biobank

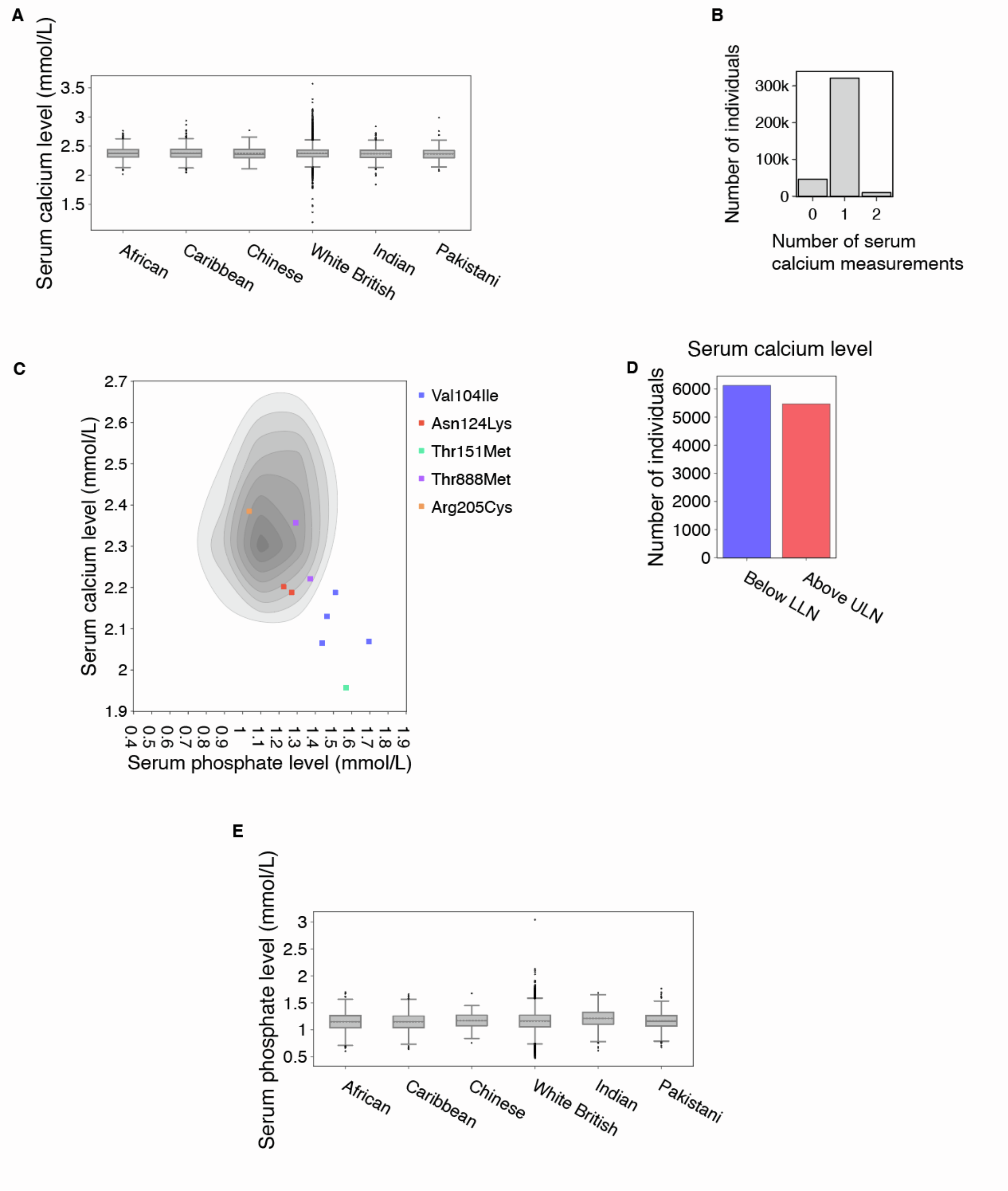

Figure caption. **Serum calcium and phosphate levels in the UK Biobank**. (A) Box plot showing serum calcium levels across self-reported ethnicities. *The boxes represent the interquartile range (IQR), the whiskers extend to 1.5 times the IQR. and the dots are outside of this range.* (B) Bar chart depicting the number of serum calcium measurements per individual in the UK Biobank, stratified by self-reported ethnicity. (C) Scatter plot showing the levels of serum calcium and serum phosphate for individuals with each variant. (D) Bar chart depicting the number of individuals with serum calcium below the lower limit of normal (LLN) or above the

upper limit of normal (ULN). (E) Box plot showing serum phosphate levels across self-reported ethnicities. *The boxes represent the interquartile range (IQR), the whiskers extend to 1.5 times the IQR. and the dots are outside of this range.*

Figure S4: Maximum scores in the UKB and AOU

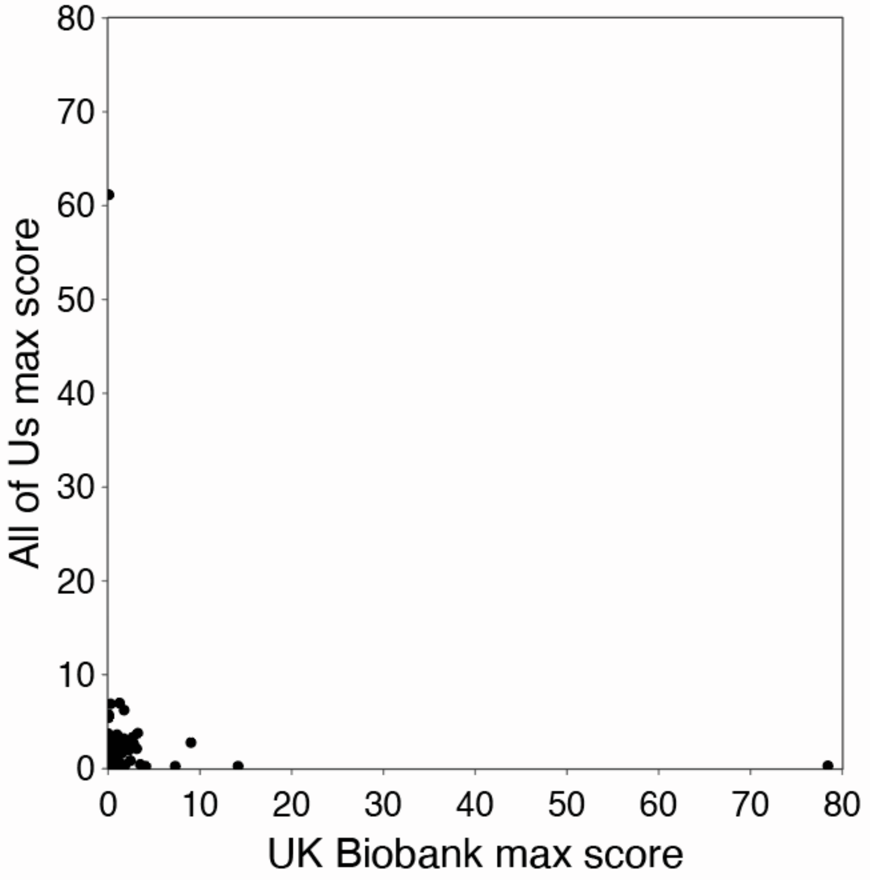

Figure caption: **Maximum scores in the UKB and AOU.** Scatter plot showing maximum scores in the UKB (on the x-axis) and AOU (on the y-axis). AOU=All of Us; UKB=UK Biobank.

Figure S5: Comparison of serum calcium effects in MGB, AOU, and UKB

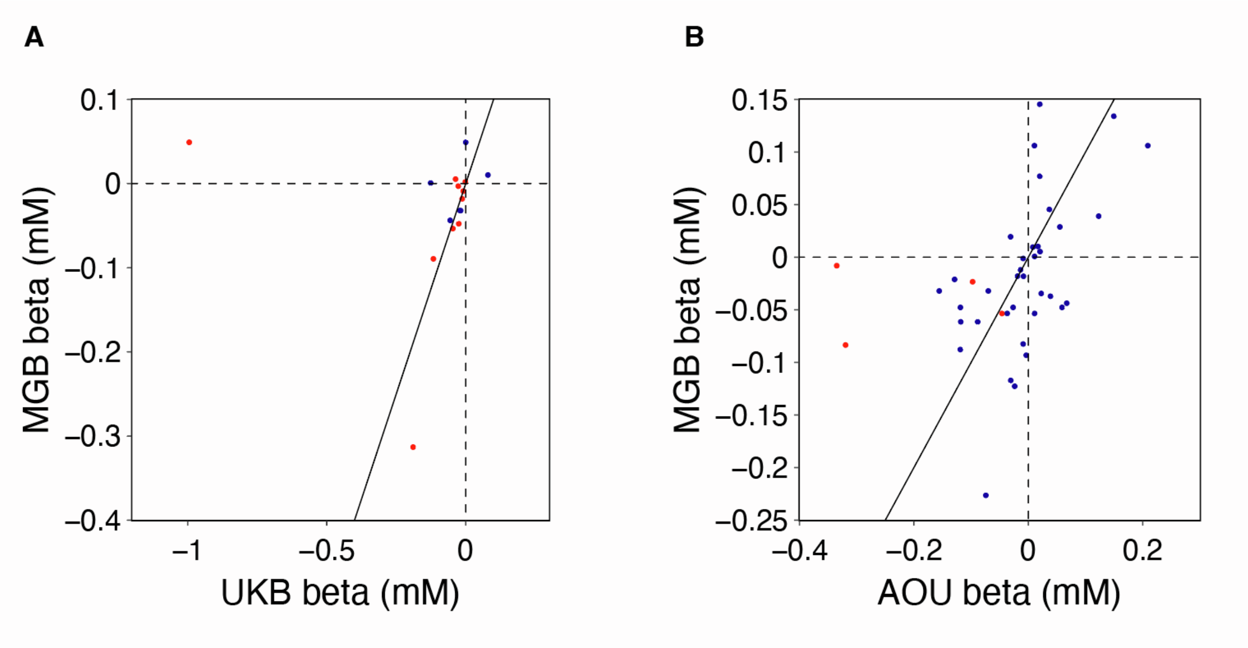

Figure caption: **Comparison of serum calcium effects in MGB, AOU, and UKB.** Scatter plot of betas of linear regression on serum calcium levels in the UKB (A) and AOU (B) compared with the MGB Biobank. All plots include only variants that have ADH1 score > 1.5. Red points correspond to variants that have a significant ($p < 0.05$) association with serum calcium in the UKB and AOU. The slope of the black line is 1. AOU=All of Us; MGB=Mass General Brigham; UKB=UK Biobank.

Figure S6 Phenome-wide association study using both known and novel ADH1-associated variants

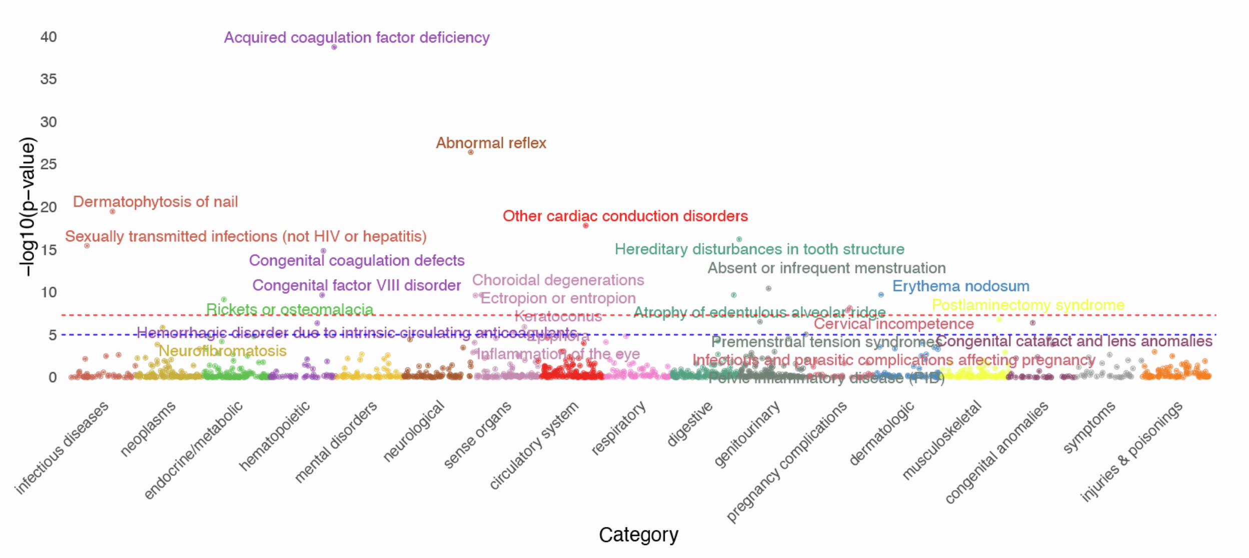

Figure caption: ***Phenome-wide association study using both known and novel ADH1-associated variants.*** *Blue dashed line indicates 1E-5 significance threshold, and red dashed line indicates 5E-8 significance threshold.*

Figure S7: Genetic architecture of serum calcium with respect to *CASR* variation

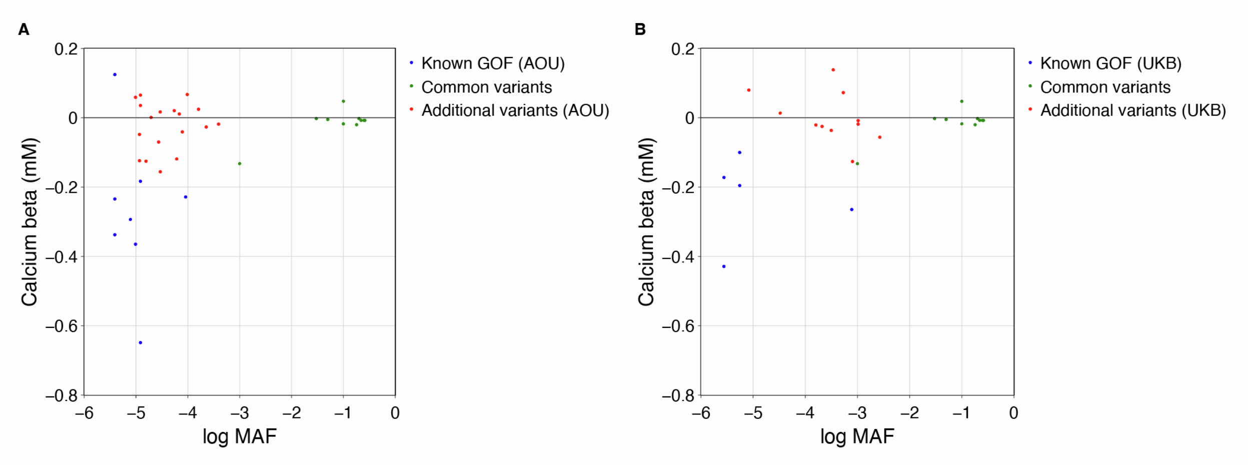

Figure caption: **Genetic architecture of serum calcium with respect to *CASR* variation.** (A, B) Scatter plot of effect on serum calcium versus $\log_{10}$ of variant minor allele frequency for various sets of variants in the UKB (A) or AOU (B).

Table S3: Sensitivity and specificity in the UK Biobank

Ethnicity Cohort	Sensitivity	Specificity
“African”	N/A	1
“Caribbean”	N/A	0.97
“Chinese”	N/A	1
“White British”	0.75	0.98
“Indian”	1	1
“Pakistani”	N/A	1

Table caption: ***Sensitivity and specificity in the UK Biobank***

Table S4: Sensitivity and specificity in All of Us

Ancestry Cohort	Sensitivity	Specificity
AFR-like	1	0.92
AMR-like	1	0.93
EAS-like	1	0.81
EUR-like	0.75	0.94
MID-like	N/A	0.88
SAS-like	N/A	0.94

Table caption: ***Sensitivity and specificity in All of Us.*** *AFR=African ancestry; AMR=American ancestry; EAS=East Asian ancestry; EUR=European ancestry; MID=Middle Eastern ancestry; SAS=South Asian ancestry.*

Table S6: UKB known ADH1 variants outside of analysis cohort

Variant	Ethnicity	Age	Sex	Ca2+ (mM)	Phosphate (mM)	Relevant meds	$eGFR_{cy}$ (mL/min/1.73m^2)	Diagnoses
p.Val104Ile [c.310G>A]	"Indian"	61	M	2.24	*1.6*	N/A	71.4	N/A
p.Glu127Gly [c.380A>G]	"British"	64	F	N/A	N/A	N/A	N/A	Chronic kidney disease, stage 3
p.Arg205Cys [c.613C>T]	"British"	43	F	2.46	1.05	N/A	122.1	N/A

*Table caption: **UKB known ADH1 variants outside of analysis cohort**. ADH1=autosomal dominant hypocalcemia type 1; Ca2+=calcium; $eGFR_{cy}$=estimated glomerular filtration rate calculated using cystatin C; F=female; M=male; meds=medications; UKB=UK Biobank.*

Table S11: In vitro testing

Variant	pEC50	SEM	n
WT	3.17	0.03	5
p.Met74Leu	2.90*	0.06	5
p.Val104Ile	3.49**	0.11	5
p.Leu87Pro	3.16	0.06	5
p.Ser247Phe	3.45*	0.07	5
p.Ala364Glu	3.49**	0.06	5
p.Ala824Gly	3.45*	0.06	5
p.Asn1074Asp	3.4	0.06	5
p.His766Gln	3.36	0.03	5

Table caption: ***In vitro testing.*** *Mean ± SEM. Log(agonist) vs. response, variable slope (four-parameter). Tested using one-way ANOVA with Dunnett's multiple comparison: *P < 0.05, **P < 0.01, **P < 0.001 vs. WT. p.Met74Leu is an FHH1 variant; p.Val104Ile is an ADH1 variant. ADH1 = autosomal dominant hypocalcemia type 1; FHH1 = familial hypocalciuric hypercalcemia type 1; N.D. = not determined;* $pEC_{50} = -\log_{10}(EC_{50})$*, where* EC_{50} *is the concentration for half-maximal response; SEM = standard error of the mean; WT = wild type.*

Table S12: Sponsored testing variants

Variant
p.Leu723Arg
p.Asp275Glu
p.Ala364Glu
p.Arg896Alafs*43
p.Thr972Met
p.Pro278Leu
p.Ile777Thr
p.Cys129Tyr
p.Ile162Val
p.Phe790Leu
p.Asn1074Asp
p.Ile187Asn
p.Asn189His
p.Ala835Pro
p.Phe798Leu
p.Glu241Lys
p.Thr151Met
p.Glu604Lys
p.Phe788Cys
p.Ala840Val
p.Gln245Arg
p.Pro221Leu
p.Phe815del
p.Glu767Lys
p.Phe806Ser
p.Phe128Leu
p.Phe832Ser

Table caption: ***Sponsored testing variants***

Table S13: Variant frequencies

Variant	Population prevalence			
	UKB	AOU	gnomAD	TOPMed
p.Gln253Lys	1.72E-05	1.12E-04	2.97E-05	1.81E-04
p.Asn345Asp	1.07E-06	2.90E-05	4.34E-06	3.40E-05
p.Ala364Glu	1.27E-04	3.10E-05	8.18E-05	2.27E-05
p.Arg678Cys	5.37E-06	1.40E-05	7.44E-06	N/A
p.Gln926Arg	9.13E-05	1.35E-04	1.13E-04	1.17E-04
p.Gly1019Arg	1.72E-05	2.00E-05	1.98E-05	4.53E-05
p.Gln1040Glu	2.15E-06	8.00E-06	4.34E-06	1.13E-05
p.Ser1061Asn	1.07E-06	2.00E-06	6.84E-07	N/A
p.Asn1074Asp	7.63E-05	2.40E-05	5.64E-05	1.89E-05

Table caption: ***Variant frequencies.*** *AOU=All of Us; UKB=UK Biobank.*

Table S14: Symptomatic odds ratios

Variant	Ethnicity	Symptomatic odds ratio (p value)
p.Gln253Lys	"Caribbean"	1.9 (0.28)
p.Asn345Asp	"African"	12.5 (0.08)
p.Ala364Glu	"White British"	1.2 (0.61)
p.Arg678Cys	"White British"	4.0 (0.23)
p.Gln926Arg	"White British"	1.1 (0.68)
p.Gly1019Arg	"Caribbean"	3.2 (0.28)
	"White British"	4.0 (0.01)
p.Gln1040Glu	"Caribbean"	4.8 (0.2)
p.Ser1061Asn	"White British"	12.0 (0.08)
p.Asn1074Asp	"White British"	1.2 (0.48)
	"African"	4.2 (0.22)

Table caption: ***Symptomatic odds ratios***

Table S17: Common variants

SNPs (GRCh37)	MAF(s)	Beta (mg/dl)	Beta (mM)	P-value(s)	Annotation(s)
3.121981609.G.A	0.1	0.19	0.0475	7.49e-583	*CASR* intronic
3.122034854.A.G	0.25	-0.03	-0.0075	2.89E-29	Intergenic
3.122010221.C.T	0.001	-0.53	-0.1325	7.26E-54	*CASR* 3 prime UTR
3.122003769.A.G	0.18	-0.08	-0.02	3.63E-125	*CASR* missense
3.121945470.T.C, 3.121945838.A.G	0.26	-0.03	-0.0075	1.91e-25 - 1.90e-25	*CASR* intronic
3.122088753.C.T, 3.122102186.A.G	0.05	-0.02	-0.005	1.07e-05 - 1.54e-05	*MIX23*, *FAM162A* non-coding
3.121907476.A.G, 3.121922222.C.G	0.22	-0.03	-0.0075	1.20e-17 - 4.76e-18	*CASR* intronic
3.121975485.G.A, 3.121984021.G.A, 3.121987328.G.C, 3.121987587.G.C, 3.121988120.A.G, 3.121988505.T.C, 3.121988738.G.A, 3.121993432.G.A, 3.121996967.TTG.T, 3.122000667.C.T, 3.122001099.T.C, 3.122005131.C.T, 3.122010681.C.T	0.2	-0.01	-0.0025	1.92e-05 - 6.53e-05	*CASR* intronic, 3 prime UTR, or downstream transcript variant
3.121950668.A.G, 3.121951168.C.T	0.03-0.04	-0.01	-0.0025	1.87e-29 - 4.32e-28	*CASR* intronic

3.121845538.T.TTATACC, 3.121847759.C.T, 3.121854556.T.A, 3.121860038.A.G, 3.121864368.C.CT, 3.121867962.T.C, 3.121869044.A.C	0.10-0.13	-0.07	-0.0175	1.05e-86 - 8.81e-87	Intergenic

*Table caption: **Common variants**. Beta (mg/dl) = effect size in milligrams per deciliter; Beta (mM) = effect size in millimoles per liter; MAF = minor allele frequency; SNP = single nucleotide polymorphism (GRCh37 reference genome).*

Supplemental Methods

UK Biobank (UKB)

The UK Biobank (UKB) is a large-scale biomedical database and research resource containing genetic, lifestyle, health and clinical information from 500,000 participants[1]. The total number of participants with data in the UKB at time of analysis was 469,835. Informed consent was obtained from all study participants, and the study was approved by the North West Multi-Centre Research Ethics Committee as a Research Tissue Bank approval. Exome sequencing, alignment, quality control (QC), and variant calling of the UKB study participants were performed as previously described[2].

We performed QC on the UKB dataset with the goal of maximizing sensitivity for identifying novel GOF *CASR* (MIM: 601199) variants. QC was performed using the PLINK (v2.00a3.1LM)[3,4] and HAIL (v0.2.78)[5,6] software packages. Since autosomal dominant hypocalcemia type 1 (ADH1, MIM: 601198)-causing variants are expected to be rare, we aimed to retain as many samples, variants, and genotypes as possible. To this end, we employed a QC pipeline consisting of three steps: sample, variant, and genotype filtering. We applied these three steps in all possible orders and then took the union of all samples, variants, and genotypes that were retained after any order of application.

Before QC, the dataset consisted of 469,835 individuals, 1,258 variants, and 591,522,265 calls based on the MANE transcript of *CASR* (ENST00000639785.2, NM_000388.4). These variants were missense, synonymous, intron, 5'- and 3'-UTR, splicing, frameshift, stop-gain, in-frame indel, or impacted a non-coding transcript. For sample QC, we removed related individuals and those with missing call rate greater than 0.01. We also removed individuals with a disagreement between self-reported and genetic sex. For variant QC, we filtered variants with a call rate less than 0.9. For genotype QC, we applied different filters for SNVs and non-SNVs to ensure genotype quality. SNVs were filtered based on a genotype quality (GQ) score greater than 19 and a sequencing depth (DP) greater than 6, whereas non-SNPs were filtered based on a GQ greater than 19 and a DP greater than 9.

Following QC, the dataset comprised 433,793 individuals (age range: 37 to 73 years, 54% female). The number of "White British" (Field 22006) individuals was 392,147. This subset consists of participants with self-reported "White British" ethnicity determined to be genetically homogeneous by principal component analysis The remaining ethnicities were determined using self-reported ethnicity (Field 21000) with the following cohort sizes: Black African: 2,925, Any other Asian background: 1,614, Any other Black background: 105, Any other mixed background: 903, Any other white background: 14,705, Asian or Asian British: 40, Bangladeshi: 206, Black or Black British: 24, British: 382,216, Caribbean: 3,792, Chinese: 1,412, Do not know: 180, Indian: 5,179, Irish: 11,339, Mixed: 37, Other ethnic group: 4,012, Pakistani: 1,580, Prefer not to answer: 1,436, White: 474, White and Asian: 718, White and Black African: 363, White and Black Caribbean: 533. Self-reported ethnicity is used as a proxy for genetic ancestry because a true genetically homogeneous population is not provided in the UKB for ancestries other than "White British". We focused our analysis on the Black African, Caribbean, Chinese, White British, Indian, and Pakistani self-reported ethnicity groups due to cohort size (total of 407,755 individuals). We removed a total of 126 individuals across ethnicities who were outliers by principal components analysis (PCA)[7]. For each PC, ethnic outliers lying more than 6 SD away from the mean were excluded. There were 566,898,080 variant calls. In total, we identified 1,253 *CASR* variants relative to its MANE transcript (ENST00000639785.2/NM_000388.4; 1,078 amino acid residues).

Serum calcium levels were uncorrected for albumin based on studies showing a superior correlation between ionized calcium and uncorrected calcium values[8]. For both serum calcium levels and serum phosphate levels, association was tested using linear regression. Age, sex, and the first 10 genetic principal components were used as covariates.

eGFR was calculated using the 2012 CKD-EPI cystatin C equation[9] due to the lower missingness rate of cystatin C measurements relative to creatinine measurements in the UKB. Association was tested using linear regression. Age, sex, and the first 10 genetic principal components were used as covariates.

For medications, the count of instances of 'calcichew', 'calcichew d3 tablet', 'colecalciferol', 'cholecalciferol', 'calcitriol', 'calcium citrate', 'calcium carbonate', 'alfacalcidol', 'PTH', 'natpar', 'teriparatide', and 'forteo' was determined for each individual. Linear regression was used to determine the association of each variant with the medication count. Age, sex, and the first 10 genetic principal components were used as covariates.

We mapped ICD-10 codes to a total of 1,518 phenotypes (phecodes) using the Phecode Map 1.2 from phewascatalog.org[11]. To maximize sensitivity, we required only a single instance of a diagnosis for defining phenotypes. We applied the phecode exclusion ranges to remove controls with related conditions[10]. If an ICD diagnosis code did not match the ICD code in the phecode mapping directly, we removed the one or two rightmost digit(s) from the ICD code diagnosis and attempted to map again until the ICD codes were successfully mapped to phecodes.

For diagnosis codes, we considered phecodes 252.2 (Hypoparathyroidism), 275.5 (Disorders of calcium or phosphorous metabolism), 350.1 (Tetany), 687.4 (Paresthesia), and 345.0/345.1/345.11/345.12 (Epilepsy). Association was tested using Fisher's exact test and, for all locus-phenotype combinations for which there are at least 10 events per variable in the logistic regression[11–13], using logistic regression with Firth correction, following standard guidelines for logistic regression analysis. Based on the similarity of results from logistic regression with Firth correction and the Fisher exact test for combinations with more than 10 events per variable (**Table S1**), we concluded that the effects of the covariates/population substructure on the association are relatively minor and that, for locus-phenotype combination with less than 10 events per covariate, where the Fisher p-values are the only available association tests, the Fisher p-values provide meaningful information. Associations for all binary phenotypes were then tested using the Fisher exact test.

For individuals with previously established ADH1-associated variants, both inpatient and primary care data (in Read2 and CTV3 format) were examined. Analyses assessing the symptom burden of pathogenic variants were restricted to individuals with both inpatient and primary care data (n = 180,334).

All regression analyses were conducted separately in each ethnicity group, and results were meta-analyzed.

All of Us (AOU)
We used data from the AOU Research Program Curated Data Repository Release v7, a longitudinal cohort study led by the National Institutes of Health for the advancement of precision medicine[14]. These data included 245,394 short read whole genome sequences. 59% of individuals were female, and ages ranged from 18 to 117. We used the genomic data available on the AoU Research Bench that had already undergone quality control. We further filtered samples for those with call rate > 0.97, variants for call rate > 0.9, and genotypes for GQ > 19. To filter out related individuals, we used the precomputed maximal subset of unrelated samples that AOU provides. Genetic ancestry was used to group individuals.

For serum calcium level, we used the concept "Calcium [mass/volume] in Serum or Plasma", for which data was available for 143,416 individuals. For serum phosphate level, we used "Phosphate [mass/volume] in Serum or Plasma", for which data was available for 57,037 individuals. We retained measurements of calcium and phosphate levels between the 0.001 (calcium: 1.51 mmol/L, phosphate: 1.30 mg/dL) and 0.999 quantiles (calcium: 2.75 mmol/L, phosphate: 7.54 mg/dL) in order to exclude potentially erroneous values. Calcium and phosphate values were then converted to millimolar (mM). Association was determined using linear regression using age, sex, and the top 10 genetic principal components as covariates.

For medications, we used concept IDs corresponding to calcitriol, colecalciferol, and teriparatide. Association was determined using linear regression using age, sex, and the top 10 genetic principal components as covariates.

For diagnosis codes, we considered the same phecodes that we used in the UK Biobank [252.2 (Hypoparathyroidism), 275.5 (Disorders of calcium or phosphorous metabolism), 350.1 (Tetany), 687.4 (Paresthesia), and 345.0/345.1/345.11/345.12 (Epilepsy)]. To capture phenotype data originally recorded using SNOMED terms in AOU, these phecodes were mapped to ICD-10 codes, which were mapped to SNOMED using the Owlready2 library's Pymedtermino2 module[15] , which utilizes UMLS data. Association was determined using Fisher's exact test.

The *All of Us* Research Program Resource Access Board (RAB) has granted an exception to the program's Data and Statistics Dissemination Policy for reporting exact participant counts of less than 20 in some of the analyses reporting in this study, due to the very low risk to participant privacy and very low risk of potential for re-identification.

Systematic Review of *CASR* variants previously associated with ADH1
In the UKB, we detected nine *CASR* variants previously associated with ADH1: c.310G>A [p.Val104Ile], c.372C>A [p.Asn124Lys], c.380A>G [p.Glu127Gly], c.452C>T [p.Thr151Met], c.613C>T [p.Arg205Cys], c.748G>A [p.Glu250Lys], c.1631G>A [p.Arg544Gln], c.2663C>T [p.Thr888Met], and c.2824G>A [p.Glu942Lys]. We subsequently reviewed the articles describing these variants and found peer-reviewed evidence supporting the pathogenicity of all[16–25] but c.748G>A [p.Glu250Lys], c.1631G>A [p.Arg544Gln], and c.2824G>A [p.Glu942Lys]. c.748G>A [p.Glu250Lys] was previously identified both in individuals with familial hypocalciuric hypercalcemia type 1 (FHH1, MIM: 145980) and ADH1 but was shown to have no impact on sensitivity to extracellular calcium *in vitro*[16]*.* c.1631G>A [p.Arg544Gln] was found in an individual with an ADH1 phenotype only in the recessive state[26] and was classified as Likely Benign with respect to ADH1 in ClinVar[27]. Similarly, c.2824G>A [p.Glu942Lys] was classified as Benign/Likely Benign in ClinVar. Thus, we excluded these three variants from the following analyses. Interestingly, c.613C>T [p.Arg205Cys] had been previously identified in both individuals with FHH1[28] and ADH1[24], although *in vitro* functional analysis was not performed. Of the remaining 6 variants, five of these variants are located in the ECD and structural analysis indicated that these variants are located in regions that are known to be important in receptor activation including the homodimer interface (p.Val104, p.Asn124, p.Glu127, p.Thr151) and ECD-cysteine-rich domain interface (p.Arg205) (**Figure S2** and **Table S5**).

In AOU, eight previously established ADH1 variants were detected (**Table 2**) across 16 individuals. These variants were in structurally important locations including the TM6-TM7 hotspot region (**Figure S1** and **Table S5**).

It is important to note that perhaps due to sample bias and the rarity of these variants, we were unable to detect associations in some variants that are well established to cause ADH1. For example, in the UKB, c.2663C>T [p.Thr888Met] showed no association with reduced serum calcium, even though the mutation disrupts negative regulation of CaSR[29] and was detected in hypocalcemic individuals[25]. Similarly, c.380A>G [p.Glu127Gly] did not show any relevant associations with ICD-10-based phecodes (and had no reported serum calcium or phosphate values), despite having been identified in a hypocalcemic individual[16] and occurring in the same location as other ADH1-associated mutations (c.380A>C [p.Glu127Ala][30] and c.379G>A [p.Glu127Lys][19,20]). In AOU, c.1810G>A [p.Glu604Lys] had no detected associations, but had also been found in multiple individuals with ADH1[16,31–33].

Variant Scoring in UKB and AOU
To prioritize pathogenic ADH1 variants, we calculated a variant score that was the sum of sub-scores indicating strength of association with ADH1 phenotypes and other characteristics consistent with a gain-of-function variant. We refer to this score as a variant's ADH1 score. We applied this score to all 479 rare (MAF<0.01) missense/nonsense/frameshift *CASR* variants as a recent paper that reviewed all ADH1 variants[34] reported 6 frameshifts and 1 nonsense variants. These variants are within the last exon of the *CASR* transcript and are likely to undergo NMD-escape to generate a protein. Several functional studies have shown that CaSR nonsense mutants produce CaSR protein, albeit at a smaller size corresponding to the amino acids lost by the truncation[35,36]. These nonsense variants usually lead to loss of part of the distal CaSR C tail that is known to bind regulators of cell surface expression such as dorfin, which mediates ubiquitin-mediated degradation[37], β-arrestin-1 that contributes to receptor internalization[38] and the putative dileucine endocytic motif that binds adaptor protein-2 and facilitates clathrin-mediated

endocytosis[39] Thus, these truncated variants have enhanced cell surface expression which increases receptor signaling and can result in ADH1. The first component of the score was association with serum calcium and phosphate levels, as measured in mmol/L. To calculate this sub-score, we applied linear regression models to each variant and serum calcium and phosphate levels and calculated the product of the β and -log10(p-values). The second component of the score was association with diagnosis code-based phenotypes (see sections for the UKB and AOU for phenotypes used). We used the sum of significance measurements (-log10(p-value)) of the resulting Fisher's exact tests as the sub-score. The third component of the score was whether individuals with the variant were taking medication related to ADH1. To calculate this sub-score, we again applied linear regression models to each variant and the number of relevant medications (for the UKB, we used all instances of p20003; for AOU, we used the concept IDs described in the AOU section). The number of appearances of 'calcichew', 'calichew d3 tablet', 'calcitriol', 'calcium citrate', 'calcium carbonate', 'colecalciferol', 'cholecalciferol', 'alfacalcidol', 'PTH', 'natpar', 'teriparatide', or 'forteo' in an individual's medical record was used as the response variable in the regression. The sub-score was the product of the β and -log10(p-values). The fourth component of the scores was whether a variant occurred at the same location as a previously described ADH1 variant, within a hotspot of ADH1 variants (amino acids 116–136[40] and 819–837[41]), or at the location of a variant discovered through a sponsored testing program. We assigned these variants a sub-score of 1. The fifth and final component of the score was the ACMG score from Varsome[42]. To calculate this sub-score, we used Varsome's API to extract the 'acmg_score', which is the result of a computational implementation of the ACMG criteria to classify variant pathogenicity[43]. We converted these scores to z-scores and lowered the weight of these scores by applying a ceiling of 0.25. This is because the ACMG criteria are not able to distinguish between GOF and LOF mutations.

As the observed empirical correlation matrix between the sub-scores showed that the sub-scores are almost uncorrelated (**Table S2**), we defined the overall score as the sum of the sub-scores. To set the weights of the sub-scores, we started with a value of 1 for all sub-scores. We then increased the value of the calcium sub-score, since hypocalcemia is observed in nearly all individuals with ADH1 described[44], and because some known variants had only associations with hypocalcemia in the UKB (such as c.310G>A [p.Val104Ile] and c.452C>T [p.Thr151Met]). We reduced the weight for predicted ACMG pathogenicity because it was unable to distinguish loss- from gain-of-function. We also reduced the weight for whether a variant occurred in a hotspot, because this was only indirect evidence of variant function. We arrived at the following weights: 3 for serum calcium, 1 for serum phosphate (since fewer, ~50%, individuals are hyperphosphatemic), 0.2 for Varsome ACMG, 0.5 for whether a variant occurs in the location of a known GOF variant or hotspot (amino acids 116–136[40] and 819–837[41] we assigned 0.5 based on the rationale that this criterion was suggestive but not sufficient to prioritize a variant), and 1 for each of the diagnosis code-based phenotypes. Variants were scored separately for each ethnicity (in the UKB) or ancestry (in AOU).

To arrive at a threshold for the ADH1 score, we created a null distribution of scores by resampling from each sub-score distribution of the synonymous variants to remove any correlation across the sub-scores (as would be expected for variants with no association with ADH1, **Figure S1**). We chose a threshold value of the score, 1.5, such that 98% of the scores from the null distribution fell below that threshold. This threshold yielded a specificity of 0.97 and sensitivity of 0.75 in the White British cohort and also similar sensitivity and specificity across all other ethnicities analyzed within the UKB based on previously known ADH1 pathogenic variants (**Table S3**). This score and threshold was then applied to all the ancestries across AOU which showed consistent sensitivity and specificity (**Table S4**).

Determination of sensitivity to extracellular calcium *in vitro*

To generate variant CaSR cell lines, WT and cmyc-tagged variant *CASR* were integrated into FlpIn TREx HEK293 cells (Invitrogen) to ensure a single copy was integrated per cell. All cells were maintained in Dulbecco's modified eagle medium (DMEM) (Thermo Fisher) supplemented with 5% fetal bovine serum (FBS) and antibiotic selection (200 µg/mL hygromycin, 5 µg/mL blasticidin). All five variants were expressed at the cell surface at levels comparable to WT CaSR (data not shown).

The response of each CaSR variant was then determined using a Ca_i^{2+} mobilization assay, which has been described in detail previously[45]. Briefly, FlpIn TRex HEK293 stable cell lines were seeded at 40,000

cells/well in poly-D-lysine (50 µg/mL) coated clear 96 well plates and incubated overnight at 37 °C in 5% CO_2 in the presence of 100 ng tetracycline. Cells were washed in assay buffer containing 150 mM NaCl, 2.6 mM KCl, 1.18 mM $MgCl_2$, 10 mM D-glucose, 10 mM HEPES, 0.1 mM $CaCl_2$, 0.5% BSA, 4 mM probenecid, pH 7.4, and loaded with 1 µM Fluo-8 AM (Abcam) in assay buffer for 1 hr. Calcium (Ca_o^{2+}) was added and measurements of Ca_i^{2+} mobilization were performed in duplicate at 37 °C using a FDSS/µCELL functional drug screening system (Hamamatsu) at 490 nm excitation and 520 nm emission. Data were normalized to the responses to assay buffer (0%) and 1 µM ionomycin (100%). The peak Ca_i^{2+} mobilization response was used for subsequent determination of the agonist response. The results are expressed at the mean ± SEM where n is the number of independent experiments. Nonlinear regression analysis was performed using GraphPad Prism® 10 (GraphPad Software, San Diego, CA).

Sponsored testing program

A genetic testing program sponsored by BridgeBio Pharma, Inc. was made available for individuals with suspected genetic hypoparathyroidism who met program eligibility criteria through Prevention Genetics. The next-generation sequencing panel has evolved to include 26 genes known to be associated with hypoparathyroidism: *ACADM* (MIM: 607008), *AIRE* (MIM: 607358), *ATP1A1* (MIM: 182310), *CASR*, *CHD7* (MIM: 608892), *CLDN16* (MIM: 603959), *CLDN19* (MIM: 610036), *CNNM2* (MIM: 607803), *DHCR7* (MIM: 602858), *EGF* (MIM: 131530), *FAM111A* (MIM: 615292), *FXYD2* (MIM: 601814), *GATA3* (MIM: 131320), *GCM2* (MIM: 603716), *GNA11* (MIM: 139313), *HADHA* (MIM: 600890), *HADHB* (MIM: 143450), *KCNA1* (MIM: 176260), *NEBL* (MIM: 605491), *PTH* (MIM: 168450), *SEMA3E* (MIM: 608166), *SLC12A3* (MIM: 600968), *SOX3* (MIM: 313430), *TBCE* (MIM: 604934), *TBX1* (MIM: 602054) and *TRPM6* (MIM: 607009).

A total of 169 samples between December 2020 and December 2022 were tested from participants with a mean ± SD age of 23.4 ± 20.4 (range 0–81) who were diagnosed with nonsurgical/idiopathic hypoparathyroidism (73.9%), hypocalcemia suspected to be of genetic cause (23.7%) or had a relative with a confirmed diagnosis of genetic hypoparathyroidism (2.4%). Pathogenic or likely pathogenic variants, and variants of uncertain significance were identified in 64 individuals (37.9%). Amongst these 64 participants, 77 variants were detected with 46.9% of variant harboring individuals documented as having unknown or no family history. In order of frequency, the relative number of individuals with detected variants are as follows: *CASR* 56.3% (36/64), *AIRE* 12.5% (8/64), *TBX1* 9.38% (6/64), *GATA3* 7.8% (5/64), *GNA11* 6.25% (4/64), *CHD7* 3.13% (2/64), *FAM111A* 3.13% (2/64), *PTH* 3.13% (2/64), *ACADM* 1.6% (1/64), *GCM2* 1.6% (1/64), *HADHB* 1.6% (1/64), and *TBCE* 1.6% (1/64). Of note, five individuals had variants identified in more than one gene.

Calculation of excess disease burden

A baseline frequency of individuals with any ADH1 phecode-based phenotype was calculated using phecodes 252.2 (Hypoparathyroidism), 275.5 (Disorders of calcium or phosphorous metabolism), 350.1 (Tetany), 687.4 (Paresthesia), 345.0/345.1/345.11/345.12 (Epilepsy), 594.0 (Urinary calculus), 594.1 (Calculus of kidney), and 585.3 (Chronic renal failure). This frequency was subtracted from the frequency of symptomatic individuals among individuals with ADH1 variants to calculate the excess frequency of symptomatic individuals among individuals with ADH1 variants. This excess frequency was multiplied by the number of individuals in each ethnicity (in the UKB) or ancestry (in AOU) to determine the excess disease burden for each variant.

Odds ratios of being symptomatic were also calculated, and p values were determined using a binomial test (**Table S13**).

Phenome-wide association study (pheWAS)

We tested for association sets of *CASR* variants with diagnosis code-based phenotypes. We calculated the burden score by assigning a value equal to the total number of alternate alleles an individual carried.

To test for associations, we performed SKAT-O test via the SKAT() command from the SKAT R package (v2.2.5)[46]. We also performed a logistic regression via the Python statsmodels package v0.14.1 to

determine the direction of effect for these variants. Age, sex, and the first 10 genetic principal components were used as covariates.

Three-dimensional modeling of CaSR structure

Snake plots were generated using GPCRdb.org[47]. CaSR three-dimensional modeling was undertaken using the reported three-dimensional cryo-EM and X-ray crystallography structures of either the CaSR ECD, CaSR ECD and TMD, and CaSR ECD and TMD with G proteins (Protein Data Bank (PDB) accession numbers: 8SZF, 8SZI, 5FBH, 7SIM, 7M3E, 7M3F, 7M3J[48–51]) in the PyMOL Molecular Graphics System (Version 2.5.2, Schrodinger, LLC).

Mass General Brigham Biobank data

The analysis in the Mass General Brigham (MGB) Biobank utilized whole-exome sequencing data for approximately 54,000 participants. Whole-exome sequencing data was conducted at the Broad Institute of Harvard and MIT. More details were described in previous publications[52]. Following removal of related individuals, we retained 39,081 individuals.

Calcium levels were averaged across repeated measurements for each participant. Close relative pairs were identified based on inferred kinship coefficients and excluded. Merging genetic and phenotype data resulted in n=35,509 participants for the calcium analysis. The analysis of calcium levels was performed using a linear regression model with sex and the first 10 genetic principal components as covariates.

Common variant fine-mapping and colocalization (SuSiE)

We used the Coloc-SuSiE[53] method to test for colocalization between serum calcium GWAS SNPs within the *CASR* region and GWAS loci for 55 other phenotypes. These phenotypes were identified through the GWAS Catalog[54], Open Targets[55], and literature searches for GWAS search terms and CASR. We identified 10 credible sets for serum calcium causal SNPs from a comprehensive biobank-based GWAS of serum calcium[56]. Each of these peaks was tested for colocalization with SNPs in the *CASR* region associated with other phenotypes, as well as GTEx[57] eQTLs for *CASR*. We applied an 80% probability threshold to determine colocalization between the serum calcium SNPs and those associated with other traits. Significant colocalization were observed for asthma, reticulocyte count, and mean corpuscular hemoglobin (probabilities of colocalization (H4) of 0.89, 0.90, and 0.99, respectively). No colocalization was observed between serum calcium variants and *CASR* expression in GTEx, likely due to limited sample sizes across tissues and significant eQTLs for *CASR* in only the pancreas (n = 328) and spleen (n = 241).

References

1. Sudlow, C., Gallacher, J., Allen, N., Beral, V., Burton, P., Danesh, J., Downey, P., Elliott, P., Green, J., Landray, M., et al. (2015). UK Biobank: An Open Access Resource for Identifying the Causes of a Wide Range of Complex Diseases of Middle and Old Age. PLoS Med. *12*, e1001779. https://doi.org/10.1371/journal.pmed.1001779.

2. Backman, J.D., Li, A.H., Marcketta, A., Sun, D., Mbatchou, J., Kessler, M.D., Benner, C., Liu, D., Locke, A.E., Balasubramanian, S., et al. (2021). Exome sequencing and analysis of 454,787 UK Biobank participants. Nature *599*, 628–634. https://doi.org/10.1038/s41586-021-04103-z.

3. Purcell, S., and Chang, C. PLINK v2.00a3.1LM. www.cog-genomics.org/plink/2.0/.

4. Chang, C.C., Chow, C.C., Tellier, L.C., Vattikuti, S., Purcell, S.M., and Lee, J.J. (2015). Second-generation PLINK: rising to the challenge of larger and richer datasets. Gigascience *4*, s13742-015-0047–0048. https://doi.org/10.1186/s13742-015-0047-8.

5. Hail-Team HAIL (v0.2.78).

6. Ganna, A., Genovese, G., Howrigan, D.P., Byrnes, A., Kurki, M.I., Zekavat, S.M., Whelan, C.W., Kals, M., Nivard, M.G., Bloemendal, A., et al. (2016). Ultra-rare disruptive and damaging mutations influence educational attainment in the general population. Nat. Neurosci. *19*, 1563–1565. https://doi.org/10.1038/nn.4404.

7. Sarnowski, C., Leong, A., Raffield, L.M., Wu, P., Vries, P.S. de, DiCorpo, D., Guo, X., Xu, H., Liu, Y., Zheng, X., et al. (2019). Impact of Rare and Common Genetic Variants on Diabetes Diagnosis by Hemoglobin A1c in Multi-Ancestry Cohorts: The Trans-Omics for Precision Medicine Program. Am. J. Hum. Genet. *105*, 706–718. https://doi.org/10.1016/j.ajhg.2019.08.010.

8. Kenny, C.M., Murphy, C.E., Boyce, D.S., Ashley, D.M., and Jahanmir, J. (2021). Things We Do for No Reason^{TM}: Calculating a “Corrected Calcium” Level. J. Hosp. Med. *16*, 499–501. https://doi.org/10.12788/jhm.3619.

9. Inker, L.A., Schmid, C.H., Tighiouart, H., Eckfeldt, J.H., Feldman, H.I., Greene, T., Kusek, J.W., Manzi, J., Lente, F.V., Zhang, Y.L., et al. (2012). Estimating Glomerular Filtration Rate from Serum Creatinine and Cystatin C. N. Engl. J. Med. *367*, 20–29. https://doi.org/10.1056/nejmoa1114248.

10. Bastarache, L. (2021). Using Phecodes for Research with the Electronic Health Record: From PheWAS to PheRS. Annu. Rev. Biomed. Data Sci. *4*, 1–19. https://doi.org/10.1146/annurev-biodatasci-122320-112352.

11. Moons, K.G.M., Groot, J.A.H. de, Bouwmeester, W., Vergouwe, Y., Mallett, S., Altman, D.G., Reitsma, J.B., and Collins, G.S. (2014). Critical Appraisal and Data Extraction for Systematic Reviews of Prediction Modelling Studies: The CHARMS Checklist. PLoS Med. *11*, e1001744. https://doi.org/10.1371/journal.pmed.1001744.

12. Moons, K.G.M., Altman, D.G., Reitsma, J.B., Ioannidis, J.P.A., Macaskill, P., Steyerberg, E.W., Vickers, A.J., Ransohoff, D.F., and Collins, G.S. (2015). Transparent Reporting of a multivariable prediction model for Individual Prognosis Or Diagnosis (TRIPOD): Explanation and Elaboration. Ann. Intern. Med. *162*, W1–W73. https://doi.org/10.7326/m14-0698.

13. Pavlou, M., Ambler, G., Seaman, S.R., Guttmann, O., Elliott, P., King, M., and Omar, R.Z. (2015). How to develop a more accurate risk prediction model when there are few events. BMJ : Br. Méd. J. *351*, h3868. https://doi.org/10.1136/bmj.h3868.

14. Investigators, A. of U.R.P., Denny, J.C., Rutter, J.L., Goldstein, D.B., Philippakis, A., Smoller, J.W., Jenkins, G., and Dishman, E. (2019). The “All of Us” Research Program. N. Engl. J. Med. *381*, 668–676. https://doi.org/10.1056/nejmsr1809937.

15. Lamy, J.-B. (2017). Owlready: Ontology-oriented programming in Python with automatic classification and high level constructs for biomedical ontologies. Artif. Intell. Med. *80*, 11–28. https://doi.org/10.1016/j.artmed.2017.07.002.

16. Hannan, F.M., Nesbit, M.A., Zhang, C., Cranston, T., Curley, A.J., Harding, B., Fratter, C., Rust, N., Christie, P.T., Turner, J.J.O., et al. (2012). Identification of 70 calcium-sensing receptor mutations in hyper- and hypo-calcaemic patients: evidence for clustering of extracellular domain mutations at calcium-binding sites. Hum. Mol. Genet. *21*, 2768–2778. https://doi.org/10.1093/hmg/dds105.

17. Hu, J., Mora, S., Colussi, G., Proverbio, M.C., Jones, K.A., Bolzoni, L., Ferrari, M.E.D., Civati, G., and Spiegel, A.M. (2009). Autosomal Dominant Hypocalcemia Caused by a Novel Mutation in the Loop 2 Region of the Human Calcium Receptor Extracellular Domain*. J. Bone Miner. Res. *17*, 1461–1469. https://doi.org/10.1359/jbmr.2002.17.8.1461.

18. Schouten, B.J., Raizis, A.M., Soule, S.G., Cole, D.R., Frengley, P.A., George, P.M., and Florkowski, C.M. (2010). Four cases of autosomal dominant hypocalcaemia with hypercalciuria including two with novel mutations in the calcium-sensing receptor gene. Ann. Clin. Biochem. *48*, 286–290. https://doi.org/10.1258/acb.2010.010139.

19. Lienhardt, A., Bai, M., Lagarde, J.-P., Rigaud, M., Zhang, Z., Jiang, Y., Kottler, M.-L., Brown, E.M., and Garabédian, M. (2001). Activating Mutations of the Calcium-Sensing Receptor: Management of Hypocalcemia. J. Clin. Endocrinol. Metab. *86*, 5313–5323. https://doi.org/10.1210/jcem.86.11.8016.

20. Hawkes, C.P., Shulman, D.I., and Levine, M.A. (2020). Recombinant human parathyroid hormone (1–84) is effective in CASR-associated hypoparathyroidism. Eur. J. Endocrinol. *183*, K13–K21. https://doi.org/10.1530/eje-20-0710.

21. Pearce, S.H.S., Williamson, C., Kifor, O., Bai, M., Coulthard, M.G., Davies, M., Lewis-Barned, N., McCredie, D., Powell, H., Kendall-Taylor, P., et al. (1996). A Familial Syndrome of Hypocalcemia with Hypercalciuria Due to Mutations in the Calcium-Sensing Receptor. N. Engl. J. Med. *335*, 1115–1122. https://doi.org/10.1056/nejm199610103351505.

22. Sørheim, J.I., Husebye, E.S., Nedrebø, B.G., Svarstad, E., Lind, J., Boman, H., and Løvås, K. (2010). Phenotypic Variation in a Large Family with Autosomal Dominant Hypocalcaemia. Horm. Res. Paediatr. *74*, 399–405. https://doi.org/10.1159/000303188.

23. Løvlie, R., Eiken, H.G., Sørheim, J.I., and Boman, H. (1996). The Ca2+-sensing receptor gene (PCAR1) mutation T151M in isolated autosomal dominant hypoparathyroidism. Hum. Genet. *98*, 129–133. https://doi.org/10.1007/s004390050174.

24. Ji, Y., Kang, C., Chen, J., and Zhang, L. (2021). Identification of p.Arg205Cys in CASR in an autosomal dominant hypocalcaemia type 1 pedigree. Medicine *100*, e26443. https://doi.org/10.1097/md.0000000000026443.

25. Lazarus, S., Pretorius, C.J., Khafagi, F., Campion, K.L., Brennan, S.C., Conigrave, A.D., Brown, E.M., and Ward, D.T. (2011). A novel mutation of the primary protein kinase C phosphorylation site in the calcium-sensing receptor causes autosomal dominant hypocalcemia. Eur. J. Endocrinol. *164*, 429–435. https://doi.org/10.1530/eje-10-0907.

26. Cavaco, B.M., Canaff, L., Nolin-Lapalme, A., Vieira, M., Silva, T.N., Saramago, A., Domingues, R., Rutter, M.M., Hudon, J., Gleason, J.L., et al. (2018). Homozygous Calcium-Sensing Receptor Polymorphism R544Q Presents as Hypocalcemic Hypoparathyroidism. J. Clin. Endocrinol. Metab. *103*, 2879–2888. https://doi.org/10.1210/jc.2017-02407.

27. Landrum, M.J., Lee, J.M., Riley, G.R., Jang, W., Rubinstein, W.S., Church, D.M., and Maglott, D.R. (2014). ClinVar: public archive of relationships among sequence variation and human phenotype. Nucleic Acids Res. *42*, D980–D985. https://doi.org/10.1093/nar/gkt1113.

28. Nissen, P.H., Christensen, S.E., Ladefoged, S.A., Brixen, K., Heickendorff, L., and Mosekilde, L. (2012). Identification of rare and frequent variants of the CASR gene by high-resolution melting. Clin. Chim. Acta *413*, 605–611. https://doi.org/10.1016/j.cca.2011.12.004.

29. Bai, M., Trivedi, S., Lane, C.R., Yang, Y., Quinn, S.J., and Brown, E.M. (1998). Protein Kinase C Phosphorylation of Threonine at Position 888 in Ca2+ o -Sensing Receptor (CaR) Inhibits Coupling to Ca2+ Store Release*. J. Biol. Chem. *273*, 21267–21275. https://doi.org/10.1074/jbc.273.33.21267.

30. Pollak, M.R., Brown, E.M., Estep, H.L., McLaine, P.N., Kifor, O., Park, J., Hebert, S.C., Seidman, C.E., and Seidman, J.G. (1994). Autosomal dominant hypocalcaemia caused by a Ca2+-sensing receptor gene mutation. Nat. Genet. *8*, 303–307. https://doi.org/10.1038/ng1194-303.

31. Alvarez-Hernandez, D., Santamaria, I., Rodriguez-Garcia, M., Iglesias, P., Delgado-Lillo, R., and Cannata-Andia, J. (2003). A novel mutation in the calcium-sensing receptor responsible for autosomal dominant hypocalcemia in a family with two uncommon parathyroid hormone polymorphisms. J. Mol. Endocrinol. *31*, 255–262. https://doi.org/10.1677/jme.0.0310255.

32. Iglesias, P., and Díez, J.J. (2006). Intracranial calcifications and activating mutation of the calcium-sensing receptor. J. Neurol., Neurosurg. Psychiatry *77*, 1243. https://doi.org/10.1136/jnnp.2006.097162.

33. García-Castaño, A., Madariaga, L., Nanclares, G.P. de, Ariceta, G., Gaztambide, S., and Castaño, L. (2019). Novel mutations associated with inherited human calcium-sensing receptor disorders: A clinical genetic study. Eur. J. Endocrinol. *180*, 59–70. https://doi.org/10.1530/eje-18-0129.

34. Roszko, K.L., Smith, L.M.S., Sridhar, A.V., Roberts, M.S., Hartley, I.R., Gafni, R.I., Collins, M.T., Fox, J.C., and Nemeth, E.F. (2022). Autosomal Dominant Hypocalcemia Type 1: A Systematic Review. J. Bone Miner. Res. *37*, 1926–1935. https://doi.org/10.1002/jbmr.4659.

35. Maruca, K., Brambilla, I., Mingione, A., Bassi, L., Capelli, S., Brasacchio, C., Soldati, L., Cisternino, M., and Mora, S. (2017). Autosomal dominant hypocalcemia due to a truncation in the C-tail of the calcium-sensing receptor. Mol. Cell. Endocrinol. *439*, 187–193. https://doi.org/10.1016/j.mce.2016.08.032.

36. Lienhardt, A., Garabédian, M., Bai, M., Sinding, C., Zhang, Z., Lagarde, J.-P., Boulesteix, J., Rigaud, M., Brown, E.M., and Kottler, M.-L. (2000). A Large Homozygous or Heterozygous In-Frame Deletion within the Calcium-Sensing Receptor's Carboxylterminal Cytoplasmic Tail That Causes Autosomal Dominant Hypocalcemia1. J. Clin. Endocrinol. Metab. *85*, 1695–1702. https://doi.org/10.1210/jcem.85.4.6570.

37. Huang, Y., Niwa, J., Sobue, G., and Breitwieser, G.E. (2006). Calcium-sensing Receptor Ubiquitination and Degradation Mediated by the E3 Ubiquitin Ligase Dorfin*. J. Biol. Chem. *281*, 11610–11617. https://doi.org/10.1074/jbc.m513552200.

38. Pi, M., Oakley, R.H., Gesty-Palmer, D., Cruickshank, R.D., Spurney, R.F., Luttrell, L.M., and Quarles, L.D. (2005). β-Arrestin- and G Protein Receptor Kinase-Mediated Calcium-Sensing Receptor Desensitization. Mol. Endocrinol. *19*, 1078–1087. https://doi.org/10.1210/me.2004-0450.

39. Nesbit, M.A., Hannan, F.M., Howles, S.A., Reed, A.A.C., Cranston, T., Thakker, C.E., Gregory, L., Rimmer, A.J., Rust, N., Graham, U., et al. (2013). Mutations in AP2S1 cause familial hypocalciuric hypercalcemia type 3. Nat. Genet. *45*, 93–97. https://doi.org/10.1038/ng.2492.

40. Wu, Y., Zhang, C., Huang, X., Cao, L., Liu, S., and Zhong, P. (2022). Autosomal dominant hypocalcemia with a novel CASR mutation: a case study and literature review. J. Int. Méd. Res. *50*, 03000605221110489. https://doi.org/10.1177/03000605221110489.

41. Hu, J., McLarnon, S.J., Mora, S., Jiang, J., Thomas, C., Jacobson, K.A., and Spiegel, A.M. (2005). A Region in the Seven-transmembrane Domain of the Human Ca2+ Receptor Critical for Response to Ca2+ *. J. Biol. Chem. *280*, 5113–5120. https://doi.org/10.1074/jbc.m413403200.

42. Kopanos, C., Tsiolkas, V., Kouris, A., Chapple, C.E., Aguilera, M.A., Meyer, R., and Massouras, A. (2018). VarSome: the human genomic variant search engine. Bioinformatics *35*, 1978–1980. https://doi.org/10.1093/bioinformatics/bty897.

43. Kopanos, C., Tsiolkas, V., Kouris, A., Chapple, C.E., Aguilera, M.A., Meyer, R., and Massouras, A. (2018). VarSome: the human genomic variant search engine. Bioinformatics *35*, 1978–1980. https://doi.org/10.1093/bioinformatics/bty897.

44. Roszko, K.L., Smith, L.M.S., Sridhar, A.V., Roberts, M.S., Hartley, I.R., Gafni, R.I., Collins, M.T., Fox, J.C., and Nemeth, E.F. (2022). Autosomal Dominant Hypocalcemia Type 1: A Systematic Review. J. Bone Miner. Res. *37*, 1926–1935. https://doi.org/10.1002/jbmr.4659.

45. Josephs, T.M., Keller, A.N., Khajehali, E., DeBono, A., Langmead, C.J., Conigrave, A.D., Capuano, B., Kufareva, I., Gregory, K.J., and Leach, K. (2020). Negative allosteric modulators of the human calcium-sensing receptor bind to overlapping and distinct sites within the 7-transmembrane domain. Br. J. Pharmacol. *177*, 1917–1930. https://doi.org/10.1111/bph.14961.

46. Lee, S., Emond, M.J., Bamshad, M.J., Barnes, K.C., Rieder, M.J., Nickerson, D.A., Team, N.G.E.S.P.L.P., Christiani, D.C., Wurfel, M.M., and Lin, X. (2012). Optimal Unified Approach for Rare-Variant Association Testing with Application to Small-Sample Case-Control Whole-Exome Sequencing Studies. Am. J. Hum. Genet. *91*, 224–237. https://doi.org/10.1016/j.ajhg.2012.06.007.

47. Pándy-Szekeres, G., Munk, C., Tsonkov, T.M., Mordalski, S., Harpsøe, K., Hauser, A.S., Bojarski, A.J., and Gloriam, D.E. (2017). GPCRdb in 2018: adding GPCR structure models and ligands. Nucleic Acids Res. *46*, gkx1109-. https://doi.org/10.1093/nar/gkx1109.

48. He, F., Wu, C.-G., Gao, Y., Rahman, S.N., Zaoralová, M., Papasergi-Scott, M.M., Gu, T.-J., Robertson, M.J., Seven, A.B., Li, L., et al. (2024). Allosteric modulation and G-protein selectivity of the Ca2+-sensing receptor. Nature *626*, 1141–1148. https://doi.org/10.1038/s41586-024-07055-2.

49. Gao, Y., Robertson, M.J., Rahman, S.N., Seven, A.B., Zhang, C., Meyerowitz, J.G., Panova, O., Hannan, F.M., Thakker, R.V., Bräuner-Osborne, H., et al. (2021). Asymmetric activation of the calcium-sensing receptor homodimer. Nature *595*, 455–459. https://doi.org/10.1038/s41586-021-03691-0.

50. Park, J., Zuo, H., Frangaj, A., Fu, Z., Yen, L.Y., Zhang, Z., Mosyak, L., Slavkovich, V.N., Liu, J., Ray, K.M., et al. (2021). Symmetric activation and modulation of the human calcium-sensing receptor. Proc. Natl. Acad. Sci. *118*, e2115849118. https://doi.org/10.1073/pnas.2115849118.

51. Zhang, C., Zhang, T., Zou, J., Miller, C.L., Gorkhali, R., Yang, J.-Y., Schilmiller, A., Wang, S., Huang, K., Brown, E.M., et al. (2016). Structural basis for regulation of human calcium-sensing receptor by magnesium ions and an unexpected tryptophan derivative co-agonist. Sci. Adv. *2*, e1600241. https://doi.org/10.1126/sciadv.1600241.

52. Boutin, N.T., Schecter, S.B., Perez, E.F., Tchamitchian, N.S., Cerretani, X.R., Gainer, V.S., Lebo, M.S., Mahanta, L.M., Karlson, E.W., and Smoller, J.W. (2022). The Evolution of a Large Biobank at Mass General Brigham. J. Pers. Med. *12*, 1323. https://doi.org/10.3390/jpm12081323.

53. Wallace, C. (2021). A more accurate method for colocalisation analysis allowing for multiple causal variants. PLoS Genet. *17*, e1009440. https://doi.org/10.1371/journal.pgen.1009440.

54. Cerezo, M., Sollis, E., Ji, Y., Lewis, E., Abid, A., Bircan, K.O., Hall, P., Hayhurst, J., John, S., Mosaku, A., et al. (2024). The NHGRI-EBI GWAS Catalog: standards for reusability, sustainability and diversity. Nucleic Acids Res. *53*, D998–D1005. https://doi.org/10.1093/nar/gkae1070.

55. Buniello, A., Suveges, D., Cruz-Castillo, C., Llinares, M.B., Cornu, H., Lopez, I., Tsukanov, K., Roldán-Romero, J.M., Mehta, C., Fumis, L., et al. (2024). Open Targets Platform: facilitating therapeutic hypotheses building in drug discovery. Nucleic Acids Res. *53*, D1467–D1475. https://doi.org/10.1093/nar/gkae1128.

56. Sakaue, S., Kanai, M., Tanigawa, Y., Karjalainen, J., Kurki, M., Koshiba, S., Narita, A., Konuma, T., Yamamoto, K., Akiyama, M., et al. (2021). A cross-population atlas of genetic associations for 220 human phenotypes. Nat. Genet. *53*, 1415–1424. https://doi.org/10.1038/s41588-021-00931-x.

57. Lonsdale, J., Thomas, J., Salvatore, M., Phillips, R., Lo, E., Shad, S., Hasz, R., Walters, G., Garcia, F., Young, N., et al. (2013). The Genotype-Tissue Expression (GTEx) project. Nat. Genet. *45*, 580–585. https://doi.org/10.1038/ng.2653.
